# Rapid dissemination of taxonomic discoveries based on DNA barcoding and morphology

**DOI:** 10.1038/srep37066

**Published:** 2016-12-19

**Authors:** Xiaowei Cao, Jie Liu, Jian Chen, Guo Zheng, Matjaž Kuntner, Ingi Agnarsson

**Affiliations:** 1 Hubei Collaborative Innovation Center for Green Transformation of Bio-Resources, Centre for Behavioural Ecology and Evolution, College of Life Sciences, Hubei University, Wuhan 430062, Hubei, China; 2 College of Life Sciences, Shenyang Normal University, Shenyang 110034, Liaoning, China; 3 Institute of Biology, Scientific Research Centre of the Slovenian Academy of Sciences and Arts, Novi Trg 2, 1000 Ljubljana, Slovenia; 4 Department of Entomology, National Museum of Natural History, Smithsonian Institution, Washington, DC, USA; 5 Department of Biology, University of Vermont, Burlington, VT, USA

## Abstract

The taxonomic impediment is characterized by dwindling classical taxonomic expertise, and slow pace of revisionary work, thus more rapid taxonomic assessments are needed. Here we pair rapid DNA barcoding methods with swift assessment of morphology in an effort to gauge diversity, establish species limits, and rapidly disseminate taxonomic information prior to completion of formal taxonomic revisions. We focus on a poorly studied, but diverse spider genus, *Pseudopoda,* from East Asia. We augmented the standard barcoding locus (COI) with nuclear DNA sequence data (ITS2) and analyzed congruence among datasets and species delimitation methods for a total of 572 individuals representing 23 described species and many potentially new species. Our results suggest that a combination of CO1 + ITS2 fragments identify and diagnose species better than the mitochondrial barcodes alone, and that certain tree based methods yield considerably higher diversity estimates than the distance-based approaches and morphology. Combined, through an extensive field survey, we detect a twofold increase in species diversity in the surveyed area, at 42–45, with most species representing short range endemics. Our study demonstrates the power of biodiversity assessments and swift dissemination of taxonomic data through rapid inventory, and through a combination of morphological and multi-locus DNA barcoding diagnoses of diverse arthropod lineages.

The turn of the millennium saw a reinforced emphasis on the “taxonomic impediment”^1^,^2^, characterized by dwindling classical taxonomic expertise. The typical slow pace of formal taxonomic revisions, combined with limited funding and lack of taxonomic jobs, translates to a significant lag between species discovery and taxonomic dissemination^3^. While notable effort and funds have been devoted to overcome this problem^2^, the taxonomic impediment persists^4^ in spite of recent deluge of modern approaches to taxonomy that relate to DNA barcoding^5^,^6^,^7^,^8^ and cyber-dissemination^9^,^10^. The problem of formal taxonomic speed of progress is furthermore exacerbated by increasing global climate changes and habitat destruction, both amplifying extinction speed^11^,^12^,^13^. Species-level taxa are the basic currency for most biological research and underlie conservation decisions, but for these purposes taxonomic information, no matter how great, is useless until it is published. The field urgently needs rapid taxonomic inventories that allow dissemination of taxonomic information—species delimitation, DNA barcodes, and morphological diagnosis—and avoid the typical taxonomic time-lag.

For hyperdiverse organisms such as many arthropods, the number of species described to date represents only a fraction of their estimated actual diversity^10^,^14^. The rate of discovery and taxonomic description of species is typically constrained by limited expertise and relatively slow pace of classical monographic research. For example, collection, examination, and description of specimens is usually challenged by deciphering taxonomic legacy including type searching, examination and interpretation, and reviewing scattered and often obscure literature. Thus, available information is withheld for years and sometimes decades before publication, a time during which this information is of little or no use to taxonomy’s end users. Truly integrative taxonomic revisions that combine genetic/genomic data with classical full blown taxonomic treatments continue to be published, but usually at slow pace, and thus more rapid taxonomic approaches may also be useful^15^. Some studies thus rely heavily on DNA barcodes for species discovery omitting formal taxonomy (paragraph below), and others emphasize rapid dissemination of informal or formal taxonomic information^16^,^17^. We explore a combination of such approaches by surveying a diverse lineage of huntsman spiders from East Asia (Fig. 1), the genus *Pseudopoda* Jäger, 2000 (Fig. 2). Our approach does not attempt in any way to replace formal taxonomic revisions, merely to speed up transmission of information.

Hebert and colleagues^6^,^7^ proposed that DNA barcoding based on a 650 bp fragment of the mitochondrial gene cytochrome oxidase subunit I (COI) can be used for species identification and delimitation across diverse animal phyla, and since then the field has seen dramatic development and explosive use^18^,^19^. Despite some early opposition^20^,^21^, DNA barcoding is now routinely used in spider taxonomy^22^,^23^,^24^,^25^,^26^,^27^, although literature continues to disagree whether COI data may suffice^19^ or not^28^ to accurately delimit species. In addition to the classical mtDNA barcoding fragment, authors are increasingly analyzing additional nuclear markers^29^,^30^ and genomic data. Genomic approaches are certainly powerful, but may be unnecessarily cumbersome (and costly) if the goal is rapid taxonomic dissemination; the data may be prohibitively burdensome to analyze while not adding critical elements to taxonomic decisions and may furthermore increase, rather than decrease, the time lag between discovery and description. However, a combination of barcodes from the biparentally inherited nuclear genome and the uniparentally inherited mitochondrial genome offers a simple and rapid way of improving the efficacy of DNA barcoding. Among nuclear DNA loci in both plants and animals, the internal transcribed spacers (ITS, ITS2) are among the most popular markers for reliable species discovery. The reasons include ease of amplification, and a combination of conserved and variable regions^31^,^32^. ITS2 has been used in spider phylogenetic^14^,^33^,^34^ and phylogeographic^29^,^30^ analyses. However, it has not to our knowledge been utilized as the second, nuclear barcoding locus in taxonomic species delimitation, as successfully done e.g. in Collembola^35^.

In our study, we aimed to: (i) assemble a DNA reference library from described and, judging from our morphological identifications, putatively new *Pseudopoda* species; (ii) test the efficacy of DNA barcodes for species discovery, delimitation, and identification; (iii) evaluate the congruence among COI and ITS2 barcodes and morphology in regards to *Pseudopoda* taxonomy; (iv) provide morphological characters to directly support each inferred *Pseudopoda* species identification; and (v) rapidly disseminate taxonomic information alleviating the ‘taxonomic lag’ of standard approaches, with the explicit aim to provide a formal revision of these in due time. Using a combination of DNA barcoding with rapid morphological assessment, we find 19–22 new species in addition to 23 previously described ones. We provide evidence and taxonomically diagnostic information for each species and through rapid dissemination make these taxa —some as yet not formally named—immediately available for end users.

## Results

### Morphology

**Figure 1:**
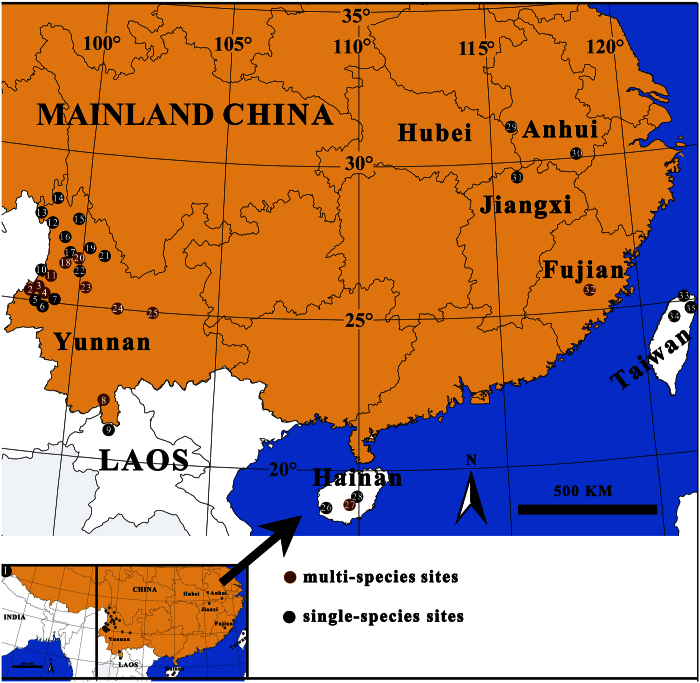
Map with sampling localities for each *Pseudopoda* species. 1. JSM (Joshimath, India): *P. prompta*; 2. LYS (Langyashan Mountain, Yunnan): *P. sp19, P. semiannulata, P. sp12, P. sp9*; 3. YFS (Yunfengshan Mountain, Yunnan): *P. sp19, P. sp12, P. sp1, P. sp6*; 4. LJPZ (Linjiapuzi Protection Station, Yunnan): *P. digitata, P. sp6, P. sinapophysis*; 5. LFS (Laifengshan Mountain, Yunnan): *P. namkhan*; 6. HKBG (Haokang Botanical Garden, Yunnan): *P. sp10*; 7. TB (Taibao Park, Yunnan): *P. namkhan*; 8. XSBN (Xishuangbanna, Yunnan): *P. confusa, P. namkhan*; 9. BTM (Ban TavanMai, Luang Nam Tha district, Laos): *P. confusa*; 10. ABM (Anti-British Monument in pianma, Yunnan): *P. sp2*; 11. YJP (Yaojiaping Protection Station, Yunnan): *P. gibberosa, P. interposita, P. sp15*; 12. PLD (Puladi Village, Yunnan): *P. sp13*; 13. KD (Kongdang Village, Yunnan): *P. sp14*; 14. FLS (Feilaisi Temple, Yunnan): *P. sp18*; 15. WFS (Wufengshan Mountain, Yunnan): *P. yunnanensis wfs*; 16. WCT (Wenchangta Tower, Yunnan): *P. bibulba we*; 17. MLP (Maliping Village, Yunnan): *P. sp11*; 18. EWS (Erwushan Mountain, Yunnan): *P. yunnanensis ews, P. bibulba we*; 19. HLT (Heilongtan Park, Yunnan): *P. sp18*; 20. QSS (Qianshishan Mountain, Yunnan): *P. sp11, P. yunnanensis qss*; 21. LYST (Lingyuanshi Temple, Yunnan): *P. sp17*; 22. YLS (Yuelingshan Mountain, Yunnan): *P. sp8*; 23. CS (Cangshan Mountain, Yunnan): *P. cangschana, P. daliensis, P. rivicola, P. signata*; 24. ZXS (Zixishan Mountain, Yunnan): *P. bibulba xz, P. sp7, P. sp16, P. signata*; 25. XS (Xishan Mountain, Yunnan): *P. bibulba xz, P. kunmingensis, P. roganda, P. spiculata*; 26. JFL (Jianfengling Mountain, Hainan): *P. bicruris*; 27. WZS (Wuzhishan Mountain, Hainan): *P. bicruris, P. mediana*; 28. BHL (Baihualing Mountain, Hainan): *P. bicruris*; 29. TTZ (Tiantangzhai National Forest Park, Hubei): *P. tiantangensis*; 30. HS (Huangshan Mountain, Anhui): *P. sp3*; 31. LS (Lushan Mountain, Jiangxi): *P. lushanensis*; 32. DYS (Daiyunshan Mountain, Fujian): *P. sp4, P. sp5*; 33. YMS (Yangmingshan Mountain, Taiwan): *P. recta*; 34. GWS (Guanwushan Mountain, Taiwan): *P. recta*; 35. LL (Loulan forest Park, Taiwan): *P. serrata*. The map was generated by ArcView GIS 3.2 (http://www.esri.com/software/arcgis/arcview).

Based on traditional diagnostic features of genitalia a total of 572 individuals belong to 42–45 species including 19–22 new species, here referred to as *Pseudopoda* sp. Females of two species previously only known from males (*P. roganda* and *P. interposita*) are reported for the first time. We provide basic morphological information including diagnostic photographs of habitus (Figure S1), palp (Figure S2) and epigyne (Figure S3), with full taxonomic treatments to follow (Cao *et al*. prep.). Most of the species are comparably small and possess similar carapace patterns, making species identification based on somatic features challenging (Figure S1). In contrast, most species are readily diagnosable using genital characters although with the following exceptions: populations of *P. bibulba (P. bibulba xz* collected from XS, ZXS and *P. bibulba we* from EWS, WCT, see Table S1) and three populations of *P. yunnanensis (P. yunnanensis wfs* from WFS*, P. yunnanensis qss* from QSS*, and P. yunnanensis ews* from EWS, see Table S1) are genetically distinct but morphologically similar. In contrast, species within the *P. sinapophysis* group including *P. sinapophysis, P. interposita, P. sp15*, and those within *P. sp2* group including *P. sp2* and *P. sp12* (Figures S2 and S3) are genetically distinct, but not readily morphologically diagnosable species. For *P. bibulba,* widespread in Yunnan Province, we collected specimens from four sites (XS, ZXS, EWS, WCT) (Fig. 1), and identified two morphs based on minor details of male palps and epigyna (Figures S2H2, S2H3, S3L2, S3L3, S3M2, S3M3): *P. bibulba we* from WCT and EWS, *P. bibulba xz* from XS and ZXS. These can be distinguished by the sharp, curved embolic end, slightly different retrolateral tibial aphophysis in males (Figures S2H2, S2H3), and the slightly broad anterior margins of the lateral lobes in females (Figures S3L2, S3L3, S3M2, S3M3). Similarly, details of genitalia can diagnose species of the *P. sinapophysis* group and *P. sp2* group, including the shape of retrolateral tibial aphophysis in males for both groups (Figures S2C3, S2C4, S2D1 for *sinapophysis* group; S2B3, S2B4 for *P. sp2* group), the anterior margins of the lateral lobes in females for *sinapophysis* group (Figures S3E1, S3E2, S3E3), and the posterior margins of the lateral lobes in females for *P. sp2* group (Figures S3C3, S3D1). For *P. yunnanensis*, also widespread in Yunnan Province, we collected many specimens from three sites (QSS, EWS, WFS) (Figure 1) belonging to three morphs respectively: *P. yunnanensis qss, P. yunnanensis ews, P. yunnanensis wfs* based on the tegular apophysis of the male palp (Figures S2J1, S2J2, S2J3), while the epigyna are not diagnostic (Figures S3O1-P3).

### Phylogenetic inference

From the total of 42–45 species analyzed here, 36–39 were represented by multiple individuals and 6 by a single individual (Table S1). All phylogenetic analyses (BI and ML for COI, ITS2, COI + ITS2) provided high resolution broadly agreeing on species limits, but with some notable differences in topology (Figures 3 and S4–8, Table 1). The BI and ML trees based on COI + ITS2 unambiguously place all individuals in cohesive, morphologically diagnostic groups, most of them strongly supported (100% bootstrap and posterior possibilities) (Figures 3 and S8, Table 1). The BI and ML trees based on COI showed that described species represented by multiple individuals were monophyletic with high support except *P. yunnanensis* that is paraphyletic (Figures S4–5, Table 1). The BI tree based on ITS2 alone also supported the monophyly of 34 of 35 putative species with the only exception of *P. serrata* nesting within *P. recta* (ML tree is nearly identical, Figures S6–7, Table 1). The other five species represented by a single-specimen each formed an isolated branch outside other species clades (Figures 3 and S4–S8).

### Species delimitation

#### Barcoding gap

COI barcodes for all 572 individuals were generated belonging to 42–45 putative species (Table S1). A subset of 140 individuals representing all but one of the putative species were amplified for ITS2. We found non-normally distributed K2P data (Kolmogorov–Smirnov test for all groups P < 0.001) for all groups, and statistically significant differences in K2P values between all intraspecific and interspecific comparisons (Mann–Whitney test P < 0.001) (Table S2). Single marker barcoding gap did not delimit the entire set of 42–45 putative species (Figure 4). However, most of putative species represented by multiple individuals had pairwise barcoding gaps. The exceptions include a couple of species with high intraspecific variation, that may represent additional cryptic diversity. Thus *P. bibulba, P. namkhan, sp18, P. yunnanensis* do not have pairwise barcoding gaps for COI (Figure S4, Table S2), and *P. daliensis* and *P. sp15* do not have pairwise barcoding gaps for ITS2 (Figure S6, Table S2). Only *P. yunnanensis* can not be separated from close relatives with a pairwise barcoding gap in the combined COI + ITS2 dataset (Figure 3, Table S2). For the “global” barcoding gaps, the concatenated COI + ITS2 dataset supported 44 species fully congruent with morphology, while single gene datasets defined 41–44 species (Figure 4, Table S2).

### Species delimitation metrics from Geneious

In general, Rosenberg’s P_AB_ statistic and P_ID_ (liberal) yielded higher number of putative species than did P_ID_ (strict) and P_RD_ (randomly distinct). For the COI dataset, all putative species were monophyletic except *P. yunnanensis*, but both P_ID_ (Strict) = 0.88 and P_ID_ (Liberal) = 0.97 supported *P. yunnanensis* (Table S3). For P_ID_ (Strict), putative species were supported by values of over 0.7, except *P. confusa* (0.47), *P. sp14* (0.58), *P. sp4* (0.58), *P. sp7* (0.58). However, at least two of the four metrics supported all species (Table S3), and for example, all clades had P_ID_ (Liberal) values above 0.9 except *P. confusa* (0.84) (Table S3). The P_RD_ (Randomly Distinct) and Rosenberg’s P_AB_ statistics also supported most species (for detail see Table S3). For the ITS2 dataset alone, and for the concatenated COI + ITS2 dataset, at least two metrics support each species hypothesis (Table S3).

#### ABGD

For the COI dataset, ABGD analyses using different parameter combinations produced non-identical results (Table S4). The analyses based on JC or K2P distances yielded 48 species under each parameter setting broadly agreeing with the above results, but further splitting up *P. bibulba, P. namkhan, P. sp18, P. yunnanensis* into *P. bibulba xz, P. bibulba* WCT, *P. bibulba* EWS, *P. namkhan* FLS, *P. namkhan* XSBN, *sp18* FLS, *sp18* HLT, *P. yunnanensis qss, P. yunnanensis ews, P. yunnanensis wfs* (Figure S4). Analyses based on p-distance yielded even more putative species (Table S4). For the ITS2 dataset the ABGD results differed under different parameter settings (Table S4), however, they broadly agreed with other methods. The analyses based on JC or K2P distance yielded 46 species (not supporting *P. prompta*) supporting the core set of 41 putative species plus additional splitting: *P. daliensis* and *P. recta* were split to two and *P. sp15* into three putative species, respectively. *P. yunnanensis* was split into *P. yunnanensis qss, P. yunnanensis ews, P. yunnanensis wfs* and *P. sp7* and *P. spiculata* were merged (Figure S6). Again, analyses based on p-distances yielded more species. The analysis of the concatenated matrix yielded broadly congruent results (Figure 3, Table S4).

#### GMYC

In general, the single-threshold GMYC model produced more species hypotheses (46–62 species), likely overly splitting genetically structured populations (Figures 3, S4 and S6). The GMYC results of COI, COI + ITS2 appear biologically unrealistic, fragmenting several morphological species into several geographic clusters (Figures 3 and S4). However, analyses of the ITS2 nuclear fragment yielded 46 putative species, which is broadly congruent with other analyses and with morphology (Figure S6).

#### bPTP

Similar to GMYC, the bPTP results imply up to 102 species, but with the analysis of the concatenated data yielding somewhat less extreme values (59 species, Fig. 3).

#### BPP

The Bayesian phylogenetics and phylogeography method was congruent with most above hypotheses showing the highest support for the 44 species hypothesis (P = 1) and only slightly lower support for the 41 species hypothesis (P = 0.983).

### Biogeographical patterns

Our results suggest that small range endemism characterizes *Pseudopoda* species, yet with several species being sympatric (Table S1, Figs 1 and 5). The phylogenetic analyses revealed strong geographic structure, as exemplified by two clades: (Eastern China; Tropical mainland China, Taiwan and Hainan Islands, Laos) (Fig. 5).

## Discussion

Accurate identification of species is a crucial step in many areas of biological research. This step can be challenging due to lack of taxonomic expertise and the long lag between species discovery and formal taxonomic revisions. Thus information, even if it is available, is inaccessible to end users before taxonomic revisions are published. On the other hand, a serious limitation to the utility of DNA barcoding as a practical taxonomic resource is the lack of accompanying morphological data, and potential misidentification of voucher specimens^36^. The importance of accurate identification is obvious and providing a detailed voucher information and morphological character evidence used for identification vastly adds to the utility of DNA barcoding^37^. Our approach here aims to mitigate these problems by rapidly disseminating taxonomic information thus allowing immediate access to reliably identified specimens that are linked to DNA barcodes and morphological voucher specimens. In addition, we provide diagnostic illustrations of key characters of each putative species.

DNA barcoding routinely uses a single mitochondrial marker to establish species limits^6^,^7^, and this approach has recently been shown to work well in spider species identification^19^ and delimitation^27^. However, some authors question the usefulness of COI data alone^28^ and rather suggest the use of multiple markers, especially for the delimitation problem. Indeed, our results show that a single mitochondrial marker in isolation provides species delimitations that differ from those when the two are combined (Tables S2–S4), and the combination of two markers more closely matches morphological and geographical data. Of the tested approaches to species delimitation, GMYC and bPTP suggested considerably higher diversity estimates compared with the more congruent, and consistent estimates employing the barcoding gap analyses, PID, BPP, and morphology. Richness seemed overestimated using GMYC when analyzing COI in isolation and the combined dataset, but not when analyzing ITS2 alone. Thus the structuring of the mtDNA data at population level seems to mislead the method which may be more appropriately used with nuclear markers.

Despite some disparity among methods, the majority of the analyses agree about a twofold increase in known *Pseudopoda* species diversity in our sample, estimated now to be between 42 and 45. The correspondence between standard morphological diagnostic features (male palps) and molecular species delimitations is very high, other than that some barcoding methods suggest additional division of morphological species.

We included only a fraction of *Pseudopoda* diversity (23/121 known species) and hence biogeographical analyses are premature, however, some clear patterns emerge from our results. Although certain species are somewhat widespread, the striking pattern we observe is that the majority of species are short range endemics, restricted to one mountain, some restricted to one natural forest park, or even a single locality (Fig. 5). In one case, for example, two closely related species (*P. interposita* and *P. sp. 15*) both are short range endemics in the same small area, yet show no overlap being separated only by a small river (Fig. 5). According to Jäger^38^ and Jäger *et al*.^39^ *Pseudopoda* species sometimes show high local species diversity because of a strong altitudinal differentiation and an inability to balloon. This may explain many small range endemics within the genus, and other spider groups, e.g. Coelotinae, in the mountainous parts of Southeast Asia^39^,^40^. Furthermore, our phylogenetic analyses indicate correspondence between geography and phylogeny, possibly hinting at independent colonization events of eastern China versus tropical mainland China, Hainan, Taiwan and Laos. We emphasize that our sample is far too limited for detailed biogeographical analyses and that more geographical sampling and further markers are necessary to fully resolve relationships, and to test biogeographical scenarios in the genus *Pseudopoda*. However, the emerging patterns of short-range endemics and geographically structured phylogeny should stimulate further research on the biogeography of the group.

DNA barcoding continues to be an important tool to aid taxonomic discovery and identification both at the species and at higher taxonomic levels^7^,^36^. The single short COI region of classical barcodes, however, can sometimes be insufficient for accurate identification, and alternative markers have been used, e.g. ITS2 in plants. In agreement with our results other recent studies have also found that mitochondrial and nuclear markers combined can outperform standard barcodes, without placing too much burden on data cost and data analysis. For example, the combination of COI and ITS2 proved useful in barcoding of Collembola^35^. DNA barcoding can become more powerful when combined with classical morphological diagnoses and dissemination of standard taxonomical information, using a variety of analytical approaches^41^,^42^,^43^,^44^,^45^.

## Conclusions

Our study demonstrates the power of rapid taxonomically driven inventories combined with swift dissemination of morphological and DNA data to rapidly disseminate taxonomic data of diverse arthropod lineages, making information available long before exhaustive revisionary work can be completed. Such approach may be especially suitable for taxonomically neglected lineages that contain considerable undiscovered diversity. With the vast majority of species remaining to be discovered and described, we advocate minimizing the lag between discovery and dissemination to facilitate biological research and conservation planning.

## Materials and Methods

### Taxonomic focus

**Figure 2:**
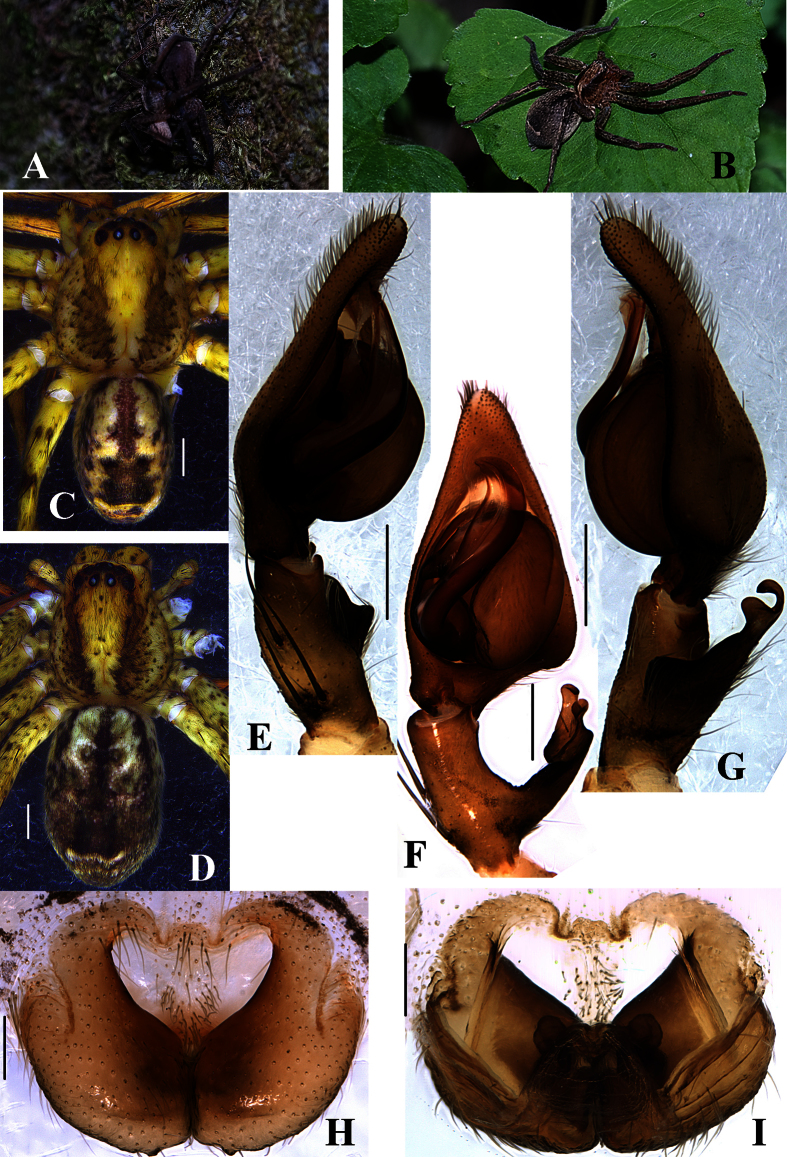
*P. yunnanensis qss* in field and its morphological details. (**A**) *P. yunnanensis qss* mating; (**B**) *P. yunnanensis qss* on leaf; (**C**) Male habitus, dorsal view; (**D**) Female habitus, dorsal view; (**E**) Male palp, prolateral view; (**F**) Male palp, ventral view; (**G**) Male palp, retrolateral view; (**H**) Epigyne, ventral view; (**I**) Vulva, dorsal view. Scale bars: (**C**, **D**) = 1 mm; E-I = 0.2 mm.

**Figure 3:**
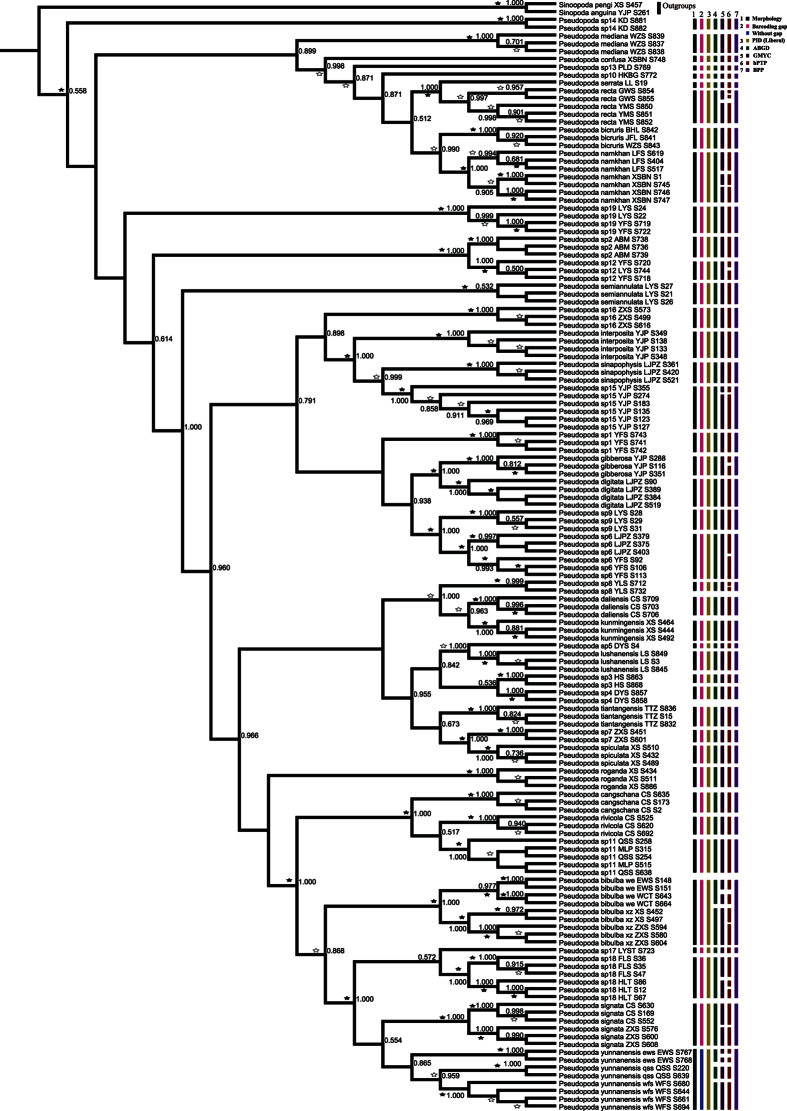
Bayesian tree based on the COI + ITS2 dataset including 140 individuals, with the results of six different species delimitation approaches in addition to morphology (see legend). Blue bar indicates barcoding overlap for focal species. Numbers on nodes are posterior probabilities; bootstrap support from ML analyses is indicated as solid stars for values >95%, open stars >50–95%.

**Figure 4:**
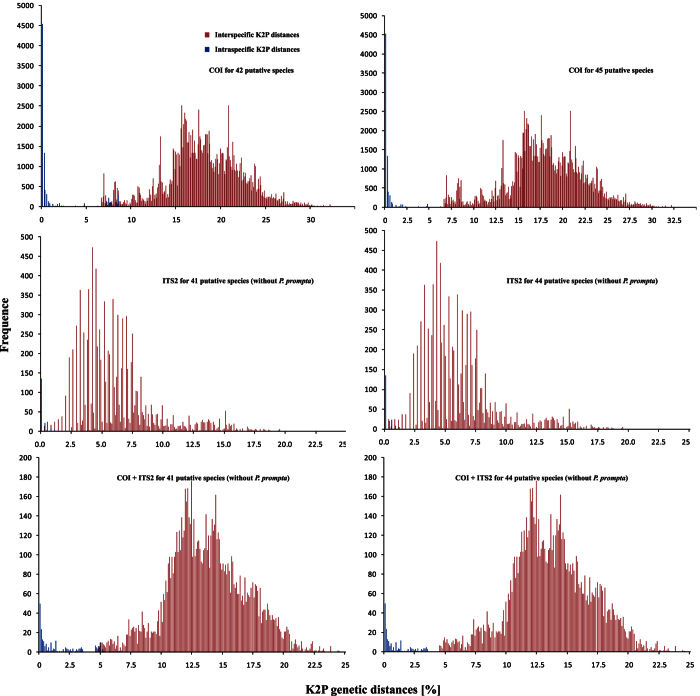
A test of the barcoding gap in *Pseudopoda* spiders based on COI, ITS2 individually and combined, with two species hypothesis (42 or 45 species for COI dataset, 41 or 44 for ITS2, COI + ITS2 datasets). Frequency distributions of intraspecific and interspecific (congeneric) genetic divergences calculated using Kimura 2-parameter (K2P) model in *Pseudopoda* spiders.

**Figure 5:**
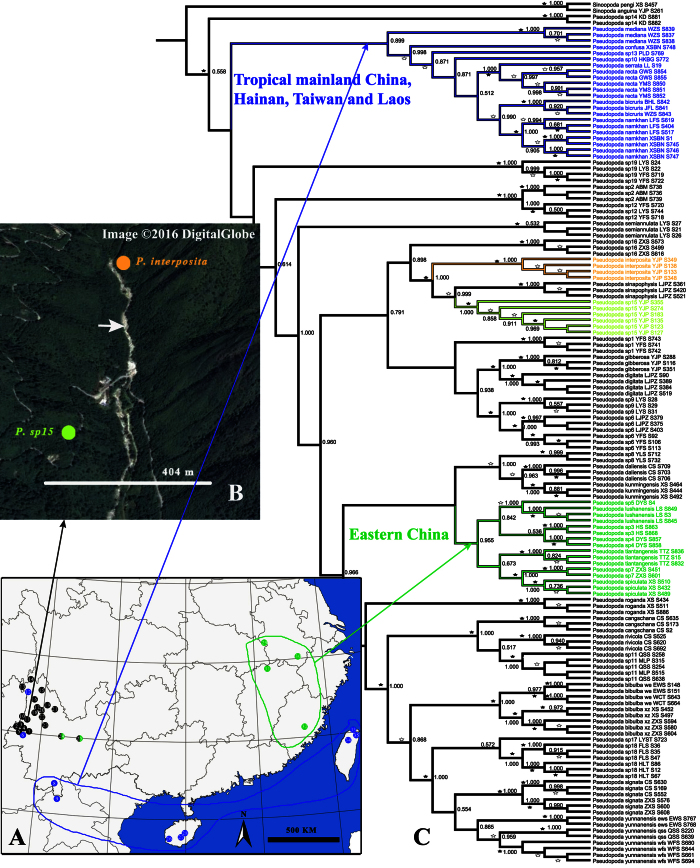
Bayesian analysis based on the COI + ITS2 dataset and geographical distributions of focal *Pseudopoda* spiders. (**A**) Map with sampling localities except JSM (Joshimath, Uttarakhand, India), generated by ArcView GIS 3.2 (http://www.esri.com/software/arcgis/arcview); (**B**) Map of sampling locality YJP (Yaojiaping Protection Station, Yunnan Province) where two closely related species (*P. interposita* and *P. sp15*) were collected, separated by a small stream arrowed (Map data: Google, DigitalGlobe); (**C**) Bayesian analysis based on the COI + ITS2 dataset, support indicated as in Figure 3. Colors represent specific clades.

With 121 described species, the genus *Pseudopoda* Jäger, 2000 (Fig. 2) is the third largest genus of the family Sparassidae^39^,^40^, although its actual species diversity is insufficiently known. Its species inhabit mountainous forests of southern, eastern and southeastern Asia^46^,^47^, and are mostly confined to distinct altitudinal zones between 300 and 3800 m^38^,^39^. About half of the known diversity, 51 species, are known from China. Most species have extremely confined distributions (e.g. *P. mediana* Quan *et al*.^46^ and *P. bicruris* Quan *et al*.^46^) but their ranges also often overlap (Figs 1 and 5). In *Pseudopoda*, morphological species delimitation is challenging due to species sympatry, habitus resemblance, and potential abundance of cryptic species. Specialists thus agree that in order to discover and identify species and to accurately resolve the taxonomy in this genus, a combination of morphological and molecular approaches is required^39^.

### Specimen collection

We sampled 572 *Pseudopoda* and 2 *Sinopoda* individuals from 35 localities in eastern China (Hubei, Jiangxi, Anhui and Fujian Provinces), southwest China (Yunnan Province), and two large islands in the south and east China sea (Hainan and Taiwan). We further obtained nucleotide data from GenBank (*P. prompta*, from India, as well as *P. confusa* and *P. namkhan*, both from Laos) (Fig. 1, Table S1). The individual name includes species name followed by the locality code and specimen code. The locality codes are the locality abbreviations using capital letters (Table S1). We used 2 *Sinopoda* species (*S. anguina, S. pengi*) as outgroups. Field collected adult specimens were fixed in absolute ethanol, and their right legs were removed to be stored at −80 °C for subsequent DNA extraction. All vouchers that we transferred to 75% ethanol for identification and morphological examination are deposited at the Centre for Behavioural Ecology and Evolution (CBEE), Hubei University, China.

### Morphology

Genitalic structures (epigyna, male palps) and habitus for each putative morphological species were digitally imaged. Male palps and epigyna were examined and photographed with Leica M205C stereomicroscope and Olympus BX51 compound microscope after being dissected from the spider bodies. The digital images depicting the habitus and genital morphology were a composite of multiple images taken at different focal planes along the Z axis and assembled using the software package Helicon Focus 3.10. Left palps were photographed unless otherwise stated. Genitalia were cleared in boiling 10% KOH for a few minutes to dissolve soft tissues.

### DNA extraction, PCR amplification and sequencing

DNA was extracted from two to four legs of each specimen using Universal Gen DNA Kit (CWBIO, Beijing, China). We used the universal primers LCOI1490 (GGTCAACAAATCATAAAGATATTGG) and HCOI2198 (TAAACTTCAGGGTGACCAAAAAATCA) for COI, as well as ITS5.8 (GGGACGATGAAGAACGCAGC) and ITS4 (TCCTCCGCTTATTGATATGC) for ITS2 to obtain PCR products following standard protocols^33^,^48^. Amplified fragments were sequenced in both directions by the Tsingke Biological Technology (Wuhan, China) and then assembled and proofread using the Chromaseq module in Mesquite employing Phred and Phrap. We generated COI barcodes for all specimens and ITS2 sequences for 140 specimens; these were chosen to represent unique COI haplotypes of all putative species, and all localities. Sequences were submitted to Genbank (accession numbers KY095934–KY096645, see Table S1).

### Phylogenetic analysis

We aligned all sequences using MAFFT^49^ through the EMBL-EBI online portal with 100 tree rebuilding replications and 100 max iterations for a thorough search otherwise using default settings. Protein coding gene sequences were translated to amino acids and confirmed to contain no stop codons. Because the 5′ and 3′ ends of some COI sequences were of poor quality, all COI sequences were trimmed to 621 bp. ITS2 sequences were also manually trimmed to 398 bp. All individual sequences were verified to belong to Sparassidae using BLASTn in GenBank. The two genes were concatenated in Mesquite. We conducted Bayesian and maximum-likelihood analyses to estimate phylogenetic relationships among species needed for species delimitation. In all analyses, gaps and ambiguous bases were treated as missing data. We created four data partitions for sensitivity analyses to explore potential data conflict: ITS2, COI1st, COI2nd, COI3rd. The appropriate models for the Bayesian analysis, selected with jModelTest2 on XSEDE (2.1.6)^50^ using the Akaike information criterion (AIC), were: GTR + I + G for COI1st and COI2nd, GTR + G for COI3rd and ITS2. Data matrices were analyzed using Bayesian inference with MrBayes 3.2.3 on XSEDE^51^ analyzing both individual genes as well as concatenated matrices. The Markov chain Monte Carlo search for each matrix ran with four chains for 50,000,000 generations sampling the Markov chain every 1,000 generations, and the sample points of the first 12,500,000 generations were discarded as ‘burnin’, after which the chains had reached approximate stationarity as determined by analysis in Tracer. Maximum likelihood analysis was done with RAxML Black Box on XSEDE^52^ on the focal matrix with same partitions as implemented in the Bayesian analysis, but using a GTR + I + G model for all partitions, keeping other parameters default. All large analyses including jModelTest2, MrBayes 3.2.3 and RAxML 8.0 were run in parallel on the CIPRES cluster at the San Diego Supercomputing Center.

### Species delimitation

We analysed individual and concatenated matrices using six species delimitation methods. As DNA barcoding gap^7^ and species delineation metrics (SDM) from Geneious require a priori species designation, we assigned the 572 individuals to 42–45 putative species according to combined analysis of phylogenetic topology from gene trees, morphological and geographic information.

We performed two DNA barcoding gap analyses, one looking at all data simultaneously (global) and the other making pairwise comparisons among species (focal)^36^. In the DNA barcoding gaps analysis, we computed genetic distances using Kimura 2-parameter (K2P) for each candidate species in Mega6^53^. We looked for statistically significant differences between intra- and interspecific K2P distances using means or medians, depending on data distribution^19^. We employed Kolmogorov–Smirnov test of normality, then performed parametric (one-way ANOVA) or nonparametric (Mann–Whitney test) statistics in SPSS.

The species delimitation plugin in Geneious v8.1.6 was utilized to obtain species delineation metrics including Rosenberg’s PAB statistic^54^, PID statistics containing PID (Strict) and PID (Liberal) and PRD. Rosenberg’s PAB statistic is the probability that a putative taxon will be monophyletic with respect to a sister clade containing “b” taxa under the null model of random coalescence^54^. The PID statistics provide the frequency with which a member of a putative species can be correctly identified given a specific alignment of sequences. The PID (Strict) requires the query sequence to fall within a monophyletic clade for an identification to be made. The PID (Liberal) requires the query sequence to fall within or to be sister to a monophyletic clade. PRD (probability randomly distinct) is the probability that a clade has the observed distinctiveness due to random coalescence. A probability value less than 0.05 rejects the null hypothesis of random coalescence and suggests that the members of a clade can be classified as species.

The automatic barcode gap discovery procedure (ABGD)^55^ sorts the terminals into hypothetical species with calculated p-values based on the barcode gap. We carried out ABGD analyses online (http://wwwabi.snv.jussieu.fr/public/abgd/), employing three different distance metrics: Jukes-Cantor (JC69), Kimura 2-parameter (K2P), and simple distance (p-distance). We analyzed the data using two different values for the parameters Pmin (0.0001 and 0.001), Pmax (0.1 and 0.2), and relative gap width (X = 0.5, 1 or 1.5), with the other parameters at default values.

The generalized mixed Yule-coalescent (GMYC) methodology^56^ uses likelihood to test for species boundaries by detecting the transition point of interspecific versus intraspecific rates of lineage coalescence. We performed GMYC analyses in the “splits” package for R. Following Xu *et al*.^27^, we used the single threshold model. We employed BEAST v. 1.8.0^57^ to obtain an ultrametric gene tree as a GMYC guide tree, under a strict molecular clock model. We used standard arthropod substitution rates for COI employing a normal prior with a mean value of 0.0115, under Yule speciation model prior and ran 50 million generations, sampling every 5000 generations. We used TRACER v 1.6^58^ to determine burn-in and to check for stationarity, then discarded as ‘burnin’ 10% of the trees in each chain to settle on an ultrametric tree using TreeAnnotator.

We carried out a Bayesian Poisson Tree Processes (bPTP) analysis employing the BI tree as input tree, as implemented online (http://species.h-its.org/ptp/)^59^. PTP is a single-locus species delimitation method using only nucleotide substitution information, implementing a model assuming gene tree branch lengths generated by two independent Poisson process classes (within- and among-species substitution events). The bPTP analysis was run using 100 000 MCMC generations, with a thinning of 100 and burn-in of 0.1, with removing the outgroups.

Finally, species delimitation on a guide tree (A10) was conducted using the program BPP 3.1^60^. This allows the use of the combined COI and ITS2 datasets to calculate the posterior probability support for each species delimitation hypothesis. In this analysis (with speciesdelimitation = 1, speciestree = 0), a reversible-jump MCMC (rjMCMC) algorithm is used to move between different species-delimitation models that are compatible with a fixed guide tree. We used the default prior for the different species tree models (speciesmodelprior = 1), which assigns equal probabilities for the rooted trees. The population size parameters (theta, θ) were assigned the gamma prior G (2, 1000), with mean 2/2000 = 0.001. The divergence time at the root of the species tree (tau, τ) was assigned the gamma prior G (2, 1000), while the other divergence time parameters are assigned the Dirichlet prior (Yang & Rannala, 2010: equation 2)^60^. These parameters were obtained and optimized by the Analysis A00 (Parameter estimation under the multispecies coalescent, with speciesdelimitation = 0, speciestree = 0) which generates the posterior distribution of species divergence times (tau, τ) and population sizes (theta, θ) under the MSC model when the species phylogeny is fixed. Each analysis was run at least twice to confirm consistency between runs. We used the BI tree as guide tree using the 41 or 44 putative species.

## Additional Information

**How to cite this article**: Cao, X. *et al*. Rapid dissemination of taxonomic discoveries based on DNA barcoding and morphology. *Sci. Rep.* **6**, 37066; doi: 10.1038/srep37066 (2016).

**Publisher's note:** Springer Nature remains neutral with regard to jurisdictional claims in published maps and institutional affiliations.

## Supplementary Material

Supplementary Information

## Figures and Tables

**Table 1:** Summary of congruence among partitions (COI, ITS2, COI + ITS2) and methods (Bayes, ML).

Species	COI		ITS2		COI + ITS2	
	BI	ML	BI	ML	BI	ML
*P. bibulba*	√(1)	√(100)	√(0.32)	√(68)	√(1)	√(99)
*P. bicruris*	√(1)	√(100)	√(0.30)	√(47)	√(1)	√(100)
*P. cangschana*	√(1)	√(100)	√(0.95)	√(96)	√(1)	√(100)
*P. confusa*	√(1)	√(100)	n/a	n/a	n/a	n/a
*P. daliensis*	√(1)	√(100)	√(0.35)	√(53)	√(1)	√(100)
*P. digitata*	√(1)	√(100)	√(0.78)	√(88)	√(1)	√(100)
*P. gibberosa*	√(1)	√(99)	√(1)	√(99)	√(1)	√(100)
*P. interposita*	√(1)	√(96)	√(0.95)	√(96)	√(1)	√(96)
*P. kunmingensis*	√(1)	√(100)	√(0.93)	√(81)	√(1)	√(100)
*P. lushanensis*	√(1)	√(100)	√(1)	√(100)	√(1)	√(100)
*P. mediana*	√(1)	√(100)	√(1)	√(100)	√(1)	√(100)
*P. namkhan*	√(1)	√(1)	√(0.98)	√(96)	√(1)	√(100)
*P. recta*	√(1)	√(99)			√(1)	√(88)
*P. rivicola*	√(1)	√(100)	√(0.83)	√(96)	√(1)	√(100)
*P. roganda*	√(1)	√(100)	√(1)	√(100)	√(1)	√(100)
*P. semiannulata*	√(0.75)	√(100)	√(0.57)	√(62)	√(0.53)	√(100)
*P. signata*	√(1)	√(100)	√(1)	√(100)	√(1)	√(100)
*P. sinapophysis*	√(1)	√(99)	√(1)	√(100)	√(1)	√(100)
*P. sp1*	√(1)	√(100)	√(0.97)	√(98)	√(1)	√(100)
*P. sp2*	√(1)	√(100)	√(1)	√(98)	√(1)	√(100)
*P. sp3*	√(1)	√(100)	√(1)	√(98)	√(1)	√(100)
*P. sp4*	√(1)	√(100)	√(1)	√(95)	√(1)	√(100)
*P. sp6*	√(1)	√(100)	√(1)	√(99)	√(1)	√(100)
*P. sp7*	√(1)	√(100)	√(0.86)	√(84)	√(1)	√(100)
*P. sp8*	√(1)	√(94)	√(0.68)	√(88)	√(1)	√(97)
*P. sp9*	√(1)	√(95)	√(1)	√(98)	√(1)	√(99)
*P. sp11*	√(1)	√(89)	√(1)	√(99)	√(1)	√(100)
*P. sp12*	√(1)	√(99)	√(1)	√(100)	√(1)	√(100)
*P. sp14*	√(1)	√(100)	√(1)	√(100)	√(1)	√(100)
*P. sp15*	√(1)	√(99)	√(1)	√(85)	√(1)	√(100)
*P. sp16*	√(1)	√(100)	√(1)	√(100)	√(1)	√(100)
*P. sp18*	√(0.9)	√(81)	√(1)	√(100)	√(1)	√(99)
*P. sp19*	√(1)	√(100)	√(0.71)		√(1)	√(95)
*P. spiculata*	√(1)	√(97)	√(0.33)	√(74)	√(1)	√(100)
*P. tiantangensis*	√(1)	√(100)	√(1)	√(90)	√(1)	√(100)
*P. yunnanensis*			√(0.99)	√(96)	√(0.87)	√(37)

Check marks indicate support for monophyly of indicated clades, blanks indicate lack of support, n/a signifies that the clade was not tested in the given analysis, due to taxon sampling. Values in parentheses indicate posterior probabilities of Bayes or bootstrap values of ML.

## References

[b1] Giangrande A. Biodiversity, conservation, and the ‘Taxonomic impediment’. Aquat. Conserv.-Mar. Freshw. Ecosyst. 13, 451–459, doi: 10.1002/aqc.584 (2003).

[b2] Rodman J. E. & Cody J. H. The taxonomic impediment overcome: NSF’s partnerships for enhancing expertise in taxonomy (PEET) as a model. Syst. Biol. 52, 428–435, doi: 10.1080/10635150309326 (2003). 12775530

[b3] Agnarsson I. & Kuntner M. Taxonomy in a changing world: Seeking solutions for a science in crisis. Syst. Biol. 56, 531–539, doi: 10.1088/10635150701424546 (2007). 17562477

[b4] Sluys R. The unappreciated, fundamentally analytical nature of taxonomy and the implications for the inventory of biodiversity. Biodiversity and Conservation 22, 1095–1105 (2013).

[b5] Hebert P. D. N. & Gregory T. R. The promise of DNA barcoding for taxonomy. Syst. Biol. 54, 852–859, doi: 10.1080/10635150500354886 (2005). 16243770

[b6] Hebert P. D., Cywinska A. & Ball S. L. Biological identifications through DNA barcodes. Proceedings of the Royal Society of London B: Biological Sciences 270, 313–321 (2003). 10.1098/rspb.2002.2218 PMC1691236 12614582

[b7] Hebert P. D., Ratnasingham S. & de Waard J. R. Barcoding animal life: cytochrome c oxidase subunit 1 divergences among closely related species. Proceedings of the Royal Society of London B: Biological Sciences 270, S96–S99 (2003). 10.1098/rsbl.2003.0025 PMC1698023 12952648

[b8] Hebert P. D. & Landry J.-F. DNA barcodes for 1/1000 of the animal kingdom. Biology letters, rsbl20090848 (2009). 10.1098/rsbl.2009.0848 PMC2880045 20015856

[b9] de Carvalho M. R. . Taxonomic impediment or impediment to taxonomy? A commentary on systematics and the cybertaxonomic-automation paradigm. Evol. Biol. 34, 140–143, doi: 10.1007/s11692-007-9011-6 (2007).

[b10] Miller J. A., Miller J. H., Dinh-Sac P. & Beentjes K. K. Cyberdiversity: Improving the Informatic Value of Diverse Tropical Arthropod Inventories. Plos One 9, doi: e11575010.1371/journal.pone.0115750 (2014). 10.1371/journal.pone.0115750 PMC4277369 25541974

[b11] Araujo M. B. & Rahbek C. How does climate change affect biodiversity? Science 313, 1396–1397, doi: 10.1126/science.1131758 (2006). 16959994

[b12] Kuntner M., Naparus M., Li D. Q. & Coddington J. A. Phylogeny predicts future habitat shifts due to climate change. Plos One 9, doi: e9890710.1371/journal.pone.0098907 (2014). 10.1371/journal.pone.0098907 PMC4044009 24892737

[b13] Sekercioglu C. H., Schneider S. H., Fay J. P. & Loarie S. R. Climate change, elevational range shifts, and bird extinctions. Conserv. Biol. 22, 140–150, doi: 10.1111/j.1523-1739.2007.00852.x (2008). 18254859

[b14] Agnarsson I., Coddington J. A. & Kuntner M. In Spider Research in the 21st Century: Trends and Perspectives (ed Penney D.) 58–111 (Siri Scientific Press, 2013).

[b15] Godfray H. C. J., Mayo S. J. & Scoble M. J. Pragmatism and Rigour can Coexist in Taxonomy. Evol. Biol. 35, 309–311, doi: 10.1007/s11692-008-9041-8 (2008).

[b16] Godfray H. C. J., Clark B. R., Kitching I. J., Mayo S. J. & Scoble M. J. The Web and the structure of taxonomy. Syst. Biol. 56, 943–955, doi: 10.1080/10635150701777521 (2007). 18066929

[b17] Clark B. R., Godfray H. C. J., Kitching I. J., Mayo S. J. & Scoble M. J. Taxonomy as an eScience. Philos. Trans. R. Soc. A-Math. Phys. Eng. Sci. 367, 953–966, doi: 10.1098/rsta.2008.0190 (2009). 19087937

[b18] Taylor H. R. & Harris W. E. An emergent science on the brink of irrelevance: a review of the past 8 years of DNA barcoding. Mol. Ecol. Resour. 12, 377–388, doi: 10.1111/j.1755-0998.2012.03119.x (2012). 22356472

[b19] Čandek K. & Kuntner M. DNA barcoding gap: reliable species identification over morphological and geographical scales. Molecular ecology resources 15, 268–277 (2015). 25042335 10.1111/1755-0998.12304

[b20] Prendini L. Comment on “Identifying spiders through DNA barcodes”. Canadian Journal of Zoology-Revue Canadienne De Zoologie 83, 498–504, doi: 10.1139/z05-025 (2005).

[b21] Hebert P. D. N. & Barrett R. D. H. Reply to the comment by L. Prendini on “Identifying spiders through DNA barcodes”. Canadian Journal of Zoology-Revue Canadienne De Zoologie 83, 505–506, doi: 10.1139/z05-026 (2005).

[b22] Barrett R. D. H. & Hebert P. D. N. Identifying spiders through DNA barcodes. Canadian Journal of Zoology-Revue Canadienne De Zoologie 83, 481–491, doi: 10.1139/z05-024 (2005).

[b23] Blagoev G. A., deWaard J. & Hebert P. D. N. Building a DNA barcode reference library for Canadian spiders (Araneae). Genome 58, 197–197 (2015).

[b24] Blagoev G. A. & Dondale C. D. A new species of Alopecosa (Araneae: Lycosidae) from Canada: a morphological description supported by DNA barcoding of 19 congeners. Zootaxa 3894, 152–160 (2014). 25544627 10.11646/zootaxa.3894.1.12

[b25] Blagoev G. A., Nikolova N. I., Sobel C. N., Hebert P. D. N. & Adamowicz S. J. Spiders (Araneae) of Churchill, Manitoba: DNA barcodes and morphology reveal high species diversity and new Canadian records. BMC Ecol. 13, doi: 4410.1186/1472-6785-13-44 (2013). 10.1186/1472-6785-13-44 PMC4222278 24279427

[b26] Hendrixson B. E., DeRussy B. M., Hamilton C. A. & Bond J. E. An exploration of species boundaries in turret-building tarantulas of the Mojave Desert (Araneae, Mygalomorphae, Theraphosidae, Aphonopelma). Mol. Phylogenet. Evol. 66, 327–340, doi: 10.1016/j.ympev.2012.10.004 (2013). 23092751

[b27] Xu X., Liu F., Chen J., Li D. & Kuntner M. Integrative taxonomy of the primitively segmented spider genus Ganthela (Araneae: Mesothelae: Liphistiidae): DNA barcoding gap agrees with morphology. Zoological Journal of the Linnean Society 175, 288–306 (2015).

[b28] Lopardo L. & Uhl G. Testing mitochondrial marker efficacy for DNA barcoding in spiders: a test case using the dwarf spider genus Oedothorax (Araneae: Linyphiidae: Erigoninae). Invert. Syst. 28, 501–521, doi: 10.1071/is14017 (2014).

[b29] Kuntner M. & Agnarsson I. Phylogeography of a successful aerial disperser: the golden orb spider *Nephila* on Indian Ocean islands. BMC Evol. Biol. 11, doi: 11910.1186/1471-2148-11-119 (2011). 10.1186/1471-2148-11-119 PMC3098804 21554687

[b30] Kuntner M. & Agnarsson I. Biogeography and diversification of hermit spiders on Indian Ocean islands (Nephilidae: *Nephilengys*). Mol. Phylogenet. Evol. 59, 477–488, doi: 10.1016/j.ympev.2011.02.002 (2011). 21316478

[b31] Chase M. W. & Fay M. F. Barcoding of Plants and Fungi. Science 325, 682–683, doi: 10.1126/science.1176906 (2009). 19644072

[b32] Fazekas A. J. . Are plant species inherently harder to discriminate than animal species using DNA barcoding markers? Mol. Ecol. Resour. 9, 130–139, doi: 10.1111/j.1755-0998.2009.02652.x (2009). 21564972

[b33] Agnarsson I. The utility of ITS2 in spider phylogenetics: notes on prior work and an example from Anelosimus. J. Arachnol. 38, 377–382 (2010).

[b34] Gregorič M., Agnarsson I., Blackledge T. A. & Kuntner M. Phylogenetic position and composition of Zygiellinae and Caerostris, with new insight into orb-web evolution and gigantism. Zoological Journal of the Linnean Society 175, 225–243 (2015).

[b35] Anslan S. & Tedersoo L. Performance of cytochrome c oxidase subunit I (COI), ribosomal DNA Large Subunit (LSU) and Internal Transcribed Spacer 2 (ITS2) in DNA barcoding of Collembola. European Journal of Soil Biology 69, 1–7 (2015).

[b36] Collins R. & Cruickshank R. The seven deadly sins of DNA barcoding. Molecular ecology resources 13, 969–975 (2013). 23280099 10.1111/1755-0998.12046

[b37] Vink C. J., Paquin P. & Cruickshank R. H. Taxonomy and Irreproducible Biological Science. Bioscience 62, 451–452, doi: 10.1525/bio.2012.62.5.3 (2012).

[b38] Jäger P. Diversität der Riesenkrabbenspinnen im Himalaya: die Radiation zweier Gattungen in den Schneetropen (Araneae: Sparassidae: Heteropodinae). (Senckenbergische Naturforschende Gesellschaft, 2001).

[b39] Jaeger P., Li S. & Krehenwinkel H. Morphological and molecular taxonomic analysis of Pseudopoda Jäger, 2000 (Araneae: Sparassidae: Heteropodinae) in Sichuan Province, China. Zootaxa 3999, 363–392 (2015). 26623582 10.11646/zootaxa.3999.3.3

[b40] Catalog W. S. World Spider Catalog. Natural History Museum Bern, online at http://wsc.nmbe.ch, version 17.0, accessed on {12, July, 2016}. (2016).

[b41] Fujita M. K., Leaché A. D., Burbrink F. T., McGuire J. A. & Moritz C. Coalescent-based species delimitation in an integrative taxonomy. Trends in ecology & evolution 27, 480–488 (2012). 22633974 10.1016/j.tree.2012.04.012

[b42] Carstens B. C., Pelletier T. A., Reid N. M. & Satler J. D. How to fail at species delimitation. Molecular ecology 22, 4369–4383 (2013). 23855767 10.1111/mec.12413

[b43] Satler J. D., Carstens B. C. & Hedin M. Multilocus species delimitation in a complex of morphologically conserved trapdoor spiders (Mygalomorphae, Antrodiaetidae, Aliatypus). Systematic Biology 62, 805–823 (2013). 23771888 10.1093/sysbio/syt041

[b44] Derkarabetian S. & Hedin M. Integrative taxonomy and species delimitation in harvestmen: a revision of the western North American genus Sclerobunus (Opiliones: Laniatores: Travunioidea). PloS one 9, e104982 (2014). 25144370 10.1371/journal.pone.0104982 PMC4140732

[b45] Hedin M. High - stakes species delimitation in eyeless cave spiders (Cicurina, Dictynidae, Araneae) from central Texas. Molecular ecology 24, 346–361 (2015). 25492722 10.1111/mec.13036

[b46] Quan D., Zhong Y. & Liu J. Four Pseudopoda species (Araneae: Sparassidae) from southern China. Zootaxa 3754, 555–571 (2014). 24869707 10.11646/zootaxa.3754.5.2

[b47] Jäger P. & Vedel V. Sparassidae of China 4. The genus Pseudopoda (Araneae: Sparassidae) in Yunnan Province. Zootaxa 1623, 1–38 (2007).

[b48] Agnarsson I., Gregorič M., Blackledge T. A. & Kuntner M. The phylogenetic placement of Psechridae within Entelegynae and the convergent origin of orb - like spider webs. Journal of Zoological Systematics and Evolutionary Research 51, 100–106 (2013).

[b49] Katoh K., Kuma K.-i., Toh H. & Miyata T. MAFFT version 5: improvement in accuracy of multiple sequence alignment. Nucleic acids research 33, 511–518 (2005). 15661851 10.1093/nar/gki198 PMC548345

[b50] Darriba D., Taboada G. L., Doallo R. & Posada D. jModelTest 2: more models, new heuristics and parallel computing. Nature methods 9, 772–772 (2012). 10.1038/nmeth.2109 PMC4594756 22847109

[b51] Huelsenbeck J. P. & Ronquist F. MRBAYES: Bayesian inference of phylogenetic trees. Bioinformatics 17, 754–755 (2001). 11524383 10.1093/bioinformatics/17.8.754

[b52] Stamatakis A. RAxML version 8: a tool for phylogenetic analysis and post-analysis of large phylogenies. Bioinformatics 30, 1312–1313 (2014). 24451623 10.1093/bioinformatics/btu033 PMC3998144

[b53] Tamura K. . MEGA5: molecular evolutionary genetics analysis using maximum likelihood, evolutionary distance, and maximum parsimony methods. Molecular biology and evolution 28, 2731–2739 (2011). 21546353 10.1093/molbev/msr121 PMC3203626

[b54] Rosenberg N. A. Statistical tests for taxonomic distinctiveness from observations of monophyly. Evolution 61, 317–323 (2007). 17348942 10.1111/j.1558-5646.2007.00023.x

[b55] Puillandre N., Lambert A., Brouillet S. & Achaz G. ABGD, Automatic Barcode Gap Discovery for primary species delimitation. Molecular ecology 21, 1864–1877 (2012). 21883587 10.1111/j.1365-294X.2011.05239.x

[b56] Pons J. . Sequence-based species delimitation for the DNA taxonomy of undescribed insects. Systematic biology 55, 595–609 (2006). 16967577 10.1080/10635150600852011

[b57] Drummond A. J., Suchard M. A., Xie D. & Rambaut A. Bayesian phylogenetics with BEAUti and the BEAST 1.7. Molecular biology and evolution 29, 1969–1973 (2012). 22367748 10.1093/molbev/mss075 PMC3408070

[b58] Rambaut A., Suchard M., Xie D. & Drummond A. Tracer v1. 6. *Computer program and documentation distributed by the author, website* http://beast.bio.ed.ac.uk/Tracer *[accessed 27 July 2014]* (2014).

[b59] Zhang J., Kapli P., Pavlidis P. & Stamatakis A. A general species delimitation method with applications to phylogenetic placements. Bioinformatics 29, 2869–2876 (2013). 23990417 10.1093/bioinformatics/btt499 PMC3810850

[b60] Yang Z. & Rannala B. Bayesian species delimitation using multilocus sequence data. Proceedings of the National Academy of Sciences 107, 9264–9269 (2010). 10.1073/pnas.0913022107 PMC2889046 20439743

