# Supplementary material for: Rapid dissemination of taxonomic discoveries based on DNA barcoding and morphology
Source: Sci Rep. 2016 Dec 19;6:37066. doi: 10.1038/srep37066 (PMC5171852; doi:10.1038/srep37066)
Supplement: Supplementary Information [file srep37066-s1.pdf]

Rapid dissemination of taxonomic discoveries based on DNA barcoding and morphology

Xiaowei Cao[1,+], Jie Liu[1,+,*], Jian Chen[1], Guo Zheng[3], Matjaž Kuntner[1,4,5], Ingi Agnarsson[2,5,*]

[1]Hubei Collaborative Innovation Center for Green Transformation of Bio-Resources, Centre for Behavioural Ecology and Evolution, College of Life Sciences, Hubei University, Wuhan 430062, Hubei, China, [2]Department of Biology, University of Vermont, Burlington, VT, USA, [3]College of Life Sciences, Shenyang Normal University, Shenyang 110034, Liaoning, China, [4]Institute of Biology, Scientific Research Centre of the Slovenian Academy of Sciences and Arts, Novi Trg 2, 1000 Ljubljana, Slovenia, [5]Department of Entomology, National Museum of Natural History, Smithsonian Institution, Washington, DC, USA

[*]Corresponding. Jie Liu, E-mail: sparassidae@aliyun.com and Ingi Agnarsson, E-mail: iagnarsson@gmail.com.

[+]These authors contributed equally to this work.

Supporting Information

Additional Supporting Information may be found in the online version of this article:

Figure S1. Habitus of Chinese *P.* spp used to test DNA barcoding, dorsal view: A1, *P. sp14*, male; A2, *P.mediana*, female; A3, *P.mediana*, male; A4, P. *confusa*, female; A5, P. *sp13*, female; A6, P. *sp10*, male; A7, *P. serrata*, female; A8, *P. recta*, female; B1, *P. bicruris*, female; B2, *P. bicruris*, male; B3, *P. namkhan*, female; B4, *P. namkhan*, male; B5, *P. sp19*, female; B6, *P. sp19*, male; B7, *P. sp2*, female; B8, *P. sp2*, male; C1, *P. sp12*, female; C2, *P. sp12*, male; C3, *P. semiannulata*, female; C4, *P. semiannulata*, male; C5, *P. sp16*, female; C6, *P. sp16*, male; C7, *P. interposita*, female; C8, *P. interposita*, male; D1, *P. sinapophysis*, female; D2, *P. sinapophysis*, male; D3, *P. sp15*, female; D4, *P. sp15*, male; D5, *P. sp1*, female; D6, *P. sp1*, male; D7, *P. gibberosa*, female; D8, *P. gibberosa*, male; E1, *P. digitata*, female; E2, *P. sp9*, female; E3, *P. sp9*, male; E4, *P. sp6*, female; E5, *P. sp6*, male; E6, *P. sp8*, female; E7, *P. sp8*, male; E8, *P. daliensis*, female; F1, *P. daliensis*, male; F2, *P. kunmingensis*, female; F3, *P. kunmingensis*, male; F4, *P. sp5*, female; F5, *P. lushanensis*, female; F6, *P. lushanensis*, male; F7, *P. sp3*, female; F8, *P. sp3*, male; G1, *P. sp4*, female; G2, *P. tiantangensis*, female; G3, *P. tiantangensis*, male; G4, *P. sp7*, female; G5, *P. sp7*, male; G6, *P. spiculata*, female; G7, *P. spiculata*, male; G8, *P. roganda*, female; H1, *P.*

roganda, male; H2, *P. cangschana*, female; H3, *P. cangschana*, male; H4, *P. rivicola*, female; H5, *P. rivicola*, male; H6, *P. sp11*, female; H7, *P. sp11*, male; H8, *P. bibulba we*, female; I1, *P. bibulba we*, male; I2, *P. bibulba xz*, female; I3, *P. bibulba xz*, male; I4, *P. sp17*, female; I5, *P. sp18*, female; I6, *P. sp18*, male; I7, *P. signata*, female; I8, *P. signata*, male; J1, *P. yunnanensis ews*, female; J2, *P. yunnanensis ews*, male; J3, *P. yunnanensis qss*, female; J4, *P. yunnanensis qss*, male; J5, *P. yunnanensis wfs*, female; J6, *P. yunnanensis wfs*, male. Scale bars: 1 mm.

Figure S2. Male palps of Chinese *P.* spp used to test DNA barcoding, ventral view: A1, *P. sp14;*A2, *P. mediana;* A3, *P. sp10;* A4, *P. bicruris;* B1, *P. namkhan;* B2, *P. sp19;* B3, *P. sp2;* B4, *P. sp12*; C1, *P. semiannulata;* C2, *P.sp16;* C3, *P. interposita;* C4, *P. sinapophysis;* D1, *P. sp15;* D2, *P. sp1;* D3, *P. gibberosa;* D4, *P. sp9*; E1, *P. sp6;* E2, *P. sp8;* E3, *P. daliensis;* E4, *P. kunmingensis;* F1, *P. lushanensis;* F2, *P. sp3;* F3, *P. tiantangensis;* F4, *P. sp7*; G1, *P. spiculata;* G2, *P. roganda;* G3, *P. cangschana;* G4, *P. rivicola;* H1, *P. sp11;* H2, *P. bibulba we*, arrow to the variation of embolic end; H3, *P. bibulba xz,* arrow to the variation of embolic end; I1, *P. sp18;* I2, *P. signata;* J1, *P. yunnanensis ews*, arrow to the variation of tegular apophysis; J2, *P. yunnanensis qqs*, arrow to the variation of tegular apophysis; J3, *P. yunnanensis wfs*, arrow to the variation of tegular apophysis. Scale bars: 0.2 mm.

Figure S3. Epigynes of Chinese *P*. spp used to test DNA barcoding, ventral view: A1, *P. mediana;* A2, *P. confusa;* A3, *P. sp13;* B1, *P. serrata;* B2, *P. recta;* B3, *P. bicruris;* C1, *P. namkhan;* C2, *P. sp19;* C3, *P. sp2;* D1, *P. sp12;* D2, *P.semiannulata;* D3, *P. sp16;* E1, *P. interposita;* E2, *P. sinapophysis;* E3, *P. sp15;* F1, *P. sp1*; F2, *P. gibberosa*; F3, *P. digitata*; G1, *P. sp9*; G2, *P. sp6*; G3, *P. sp8*; H1, *P. daliensis*; H2, *P. kunmingensis*; H3, *P. sp5*; I1, *P. lushanensis*; I2, *P. sp3*; I3, *P. sp4*; J1, *P. tiantangensis*; J2, *P. sp7*; J3, *P. spiculat*a; K1, *P. roganda;* K2, *P. cangschana;* K3, *P. rivicola;* L1, *P. sp11;* L2, *P. bibulba we,* arrow to the variation of anterior margins of the lateral lobes*;* L3, *P. bibulba xz,* arrow to the variation of anterior margins of the lateral lobes*;* M1, *P. sp17;* M2, *P. bibulba we,* arrow to the variation of first winding of the internal ducts system*;* M3, *P. bibulba xz,* arrow to the variation of first winding of the internal ducts system*;* N1, *P. sp18;* N2, *P. signata;* O1, *P. yunnanensis ews;* O2, *P. yunnanensis qqs;* O3, *P. yunnanensis wfs;* P1, *P. yunnanensis ews;* P2, *P. yunnanensis qqs;* P3, *P. yunnanensis wfs.* Scale bars: 0.2 mm.

Figure S4. Bayesian analysis based on the COI dataset including 573 individuals, with the results of five different species delimitation approaches in addition to morphology (see legend). Blue bar of barcoding gap indicated the presence of barcoding overlap for focal species.

Numbers on nodes are posterior probabilities; bootstrap support from ML analyses is indicated: solid stars indicate bootstrap support values>95%, open stars>50–95%, and nodes with less than 50% support lack stars.

Figure S5. Results of ML analysis based on the COI dataset including 573 individuals, numbers on nodes are bootstrap values.

Figure S6. Bayesian analysis based on the ITS2 dataset including 140 individuals, with the results of four different species delimitation approaches in addition to morphology (see legend). Blue bar of barcoding gap indicated the presence of barcoding overlap for focal species. Numbers on nodes are posterior probabilities; bootstrap support from ML analyses is indicated: solid stars indicate bootstrap support values>95%, open stars>50–95%, and nodes with less than 50% support lack stars.

Figure S7. Results of ML analysis based on the ITS2 dataset including 140 individuals, numbers on nodes are bootstrap values.

Figure S8. Results of ML analysis based on the COI + ITS2 dataset including 140 individuals, numbers on nodes are bootstrap values.

Table S1. List of voucher specimens sequenced for this study, collection

data and Genbank accession numbers. All species belongs to family Sparassidae. Sparassidae genera and species are listed in alphabetical order.

Table S2. Descriptive statistics for intraspecific and interspecific K2P (Kimura 2-parameter) distances, number of individuals and comparisons for each focal *Pseudopoda* species based on COI, ITS2 and COI+ITS2 datasets.

Table S3. Comparison of species delineation metrics from Geneious using data from COI, ITS2 and COI+ITS2.

Table S4. Results of the Automatic Barcode Gap Discovery (ABGD) analyses using data from COI, ITS2 and COI+ITS2.

Fig. S1

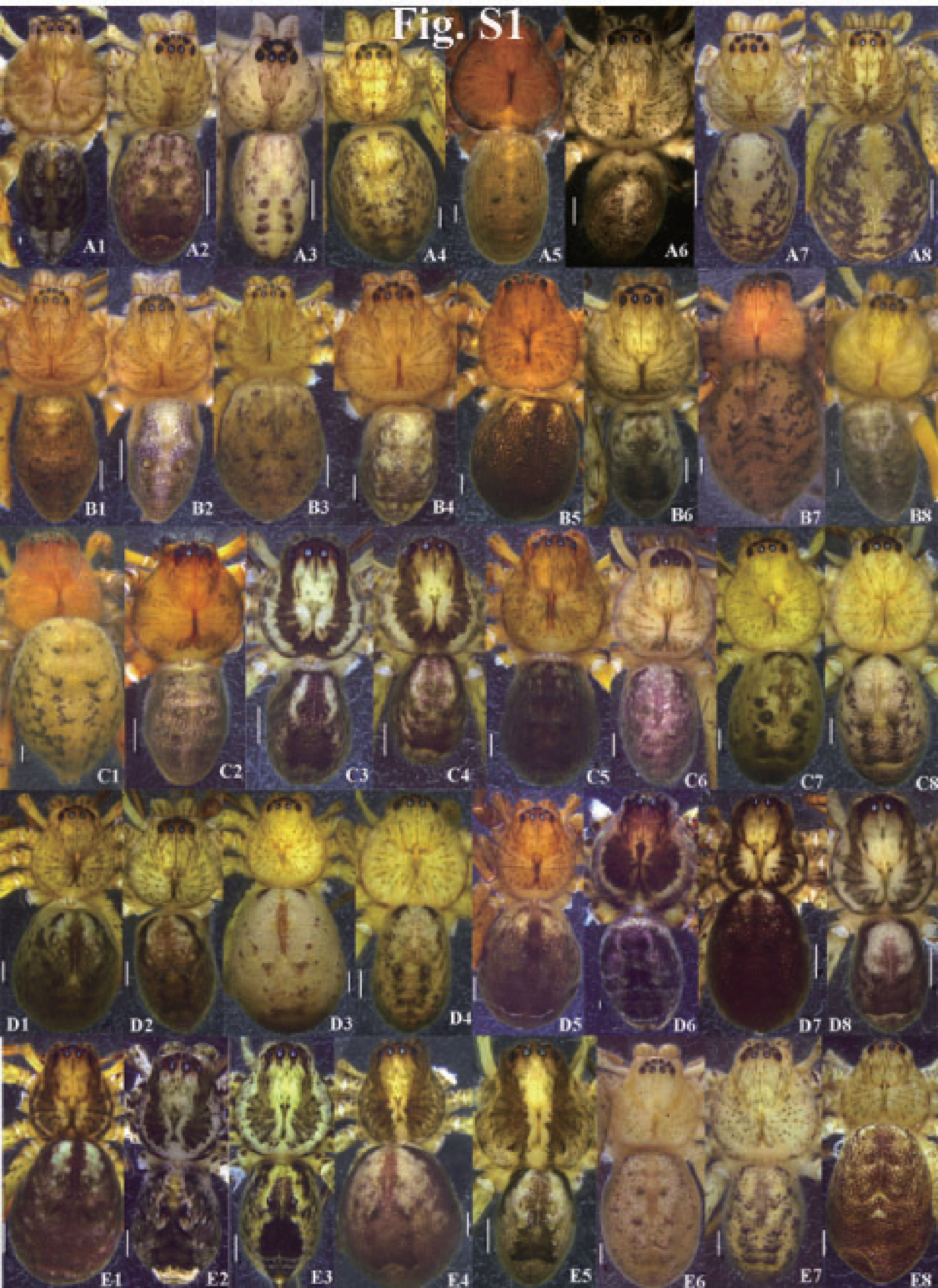
A1
A2
A3
A4
A5
A6
A7
A8
B1
B2
B3
B4
B5
B6
B7
B8
C1
C2
C3
C4
C5
C6
C7
C8
D1
D2
D3
D4
D5
D6
D7
D8
E1
E2
E3
E4
E5
E6
E7
E8

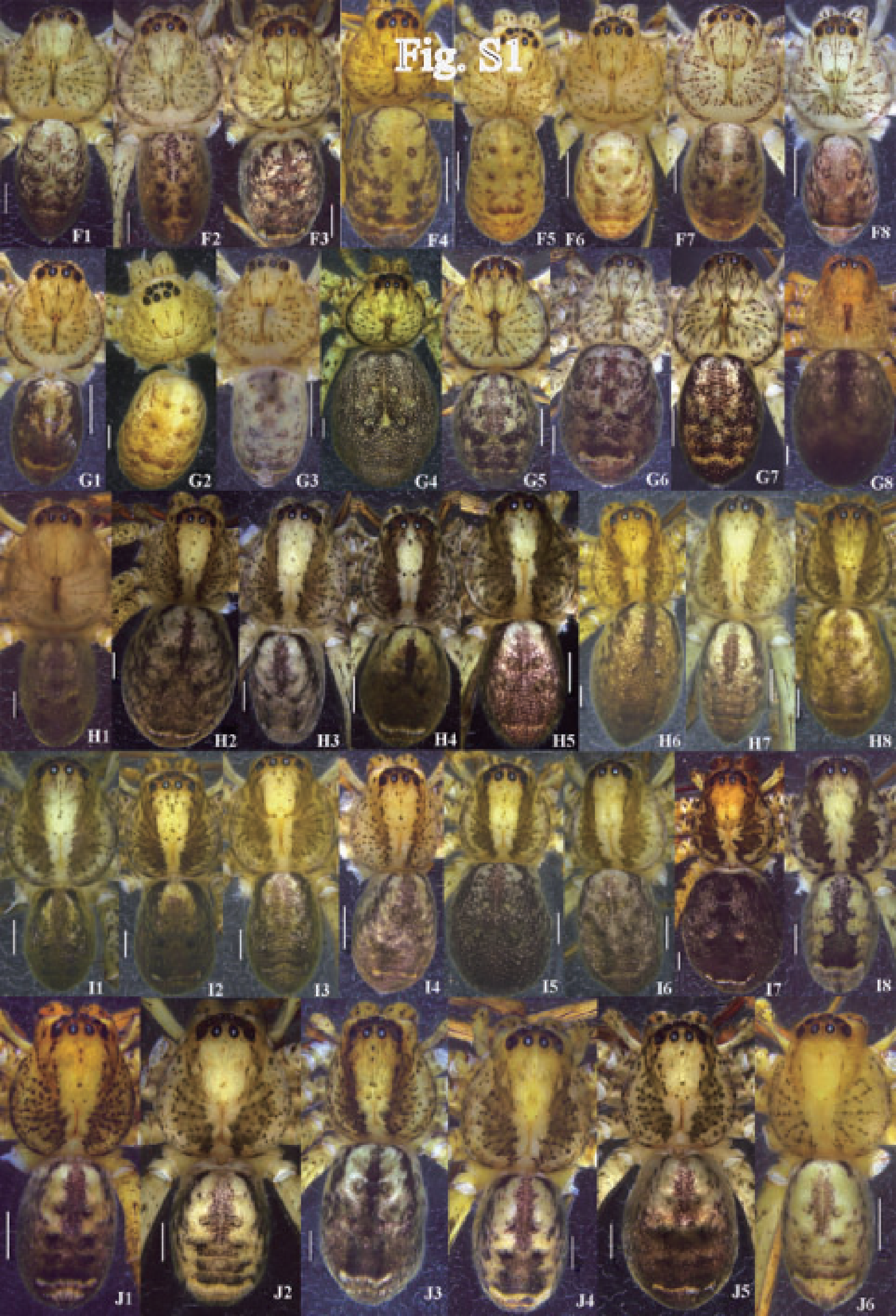
Fig. S1
F1
F2
F3
F4
F5
F6
F7
F8
G1
G2
G3
G4
G5
G6
G7
G8
H1
H2
H3
H4
H5
H6
H7
H8
I1
I2
I3
I4
I5
I6
I7
I8
J1
J2
J3
J4
J5
J6

Fig. S2

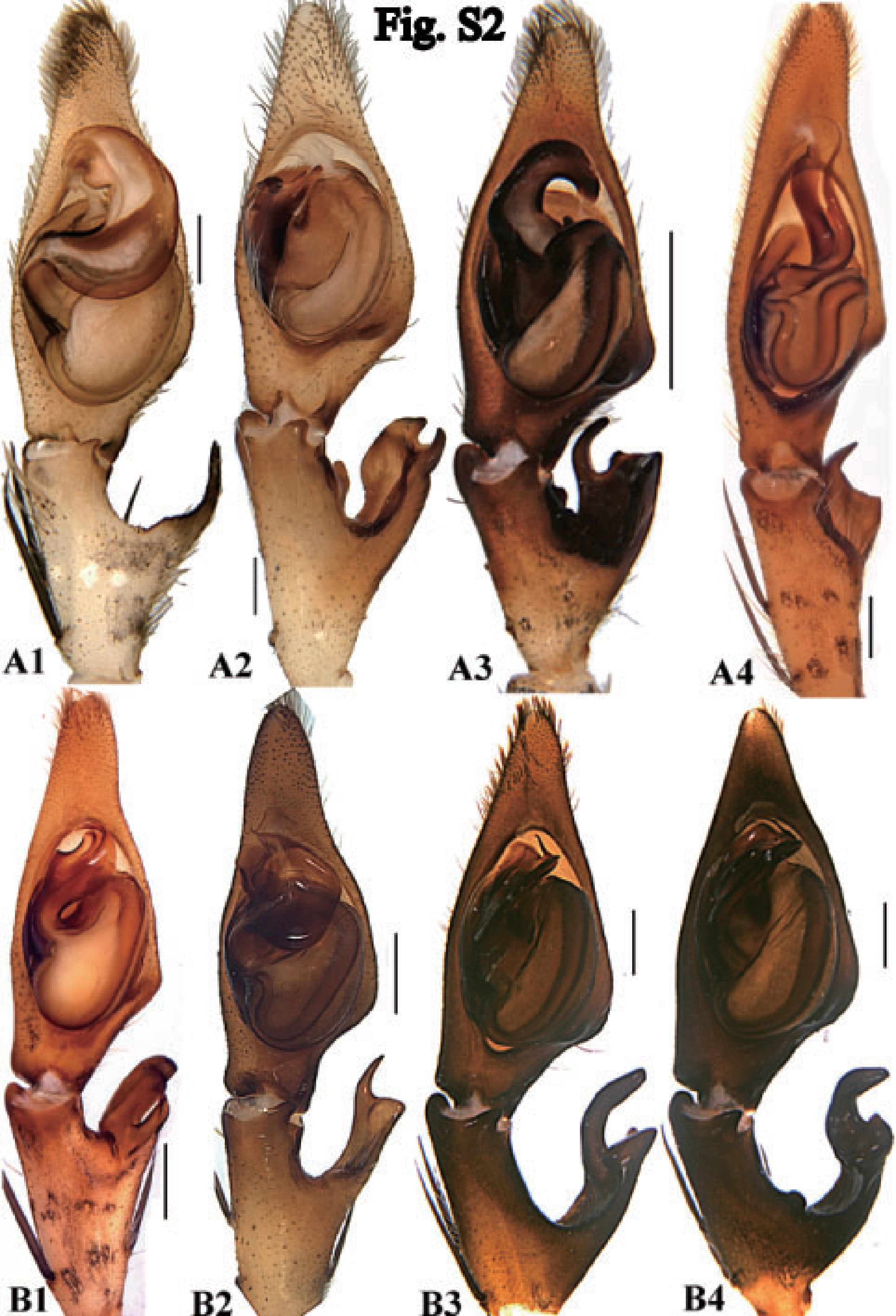

Fig. S2

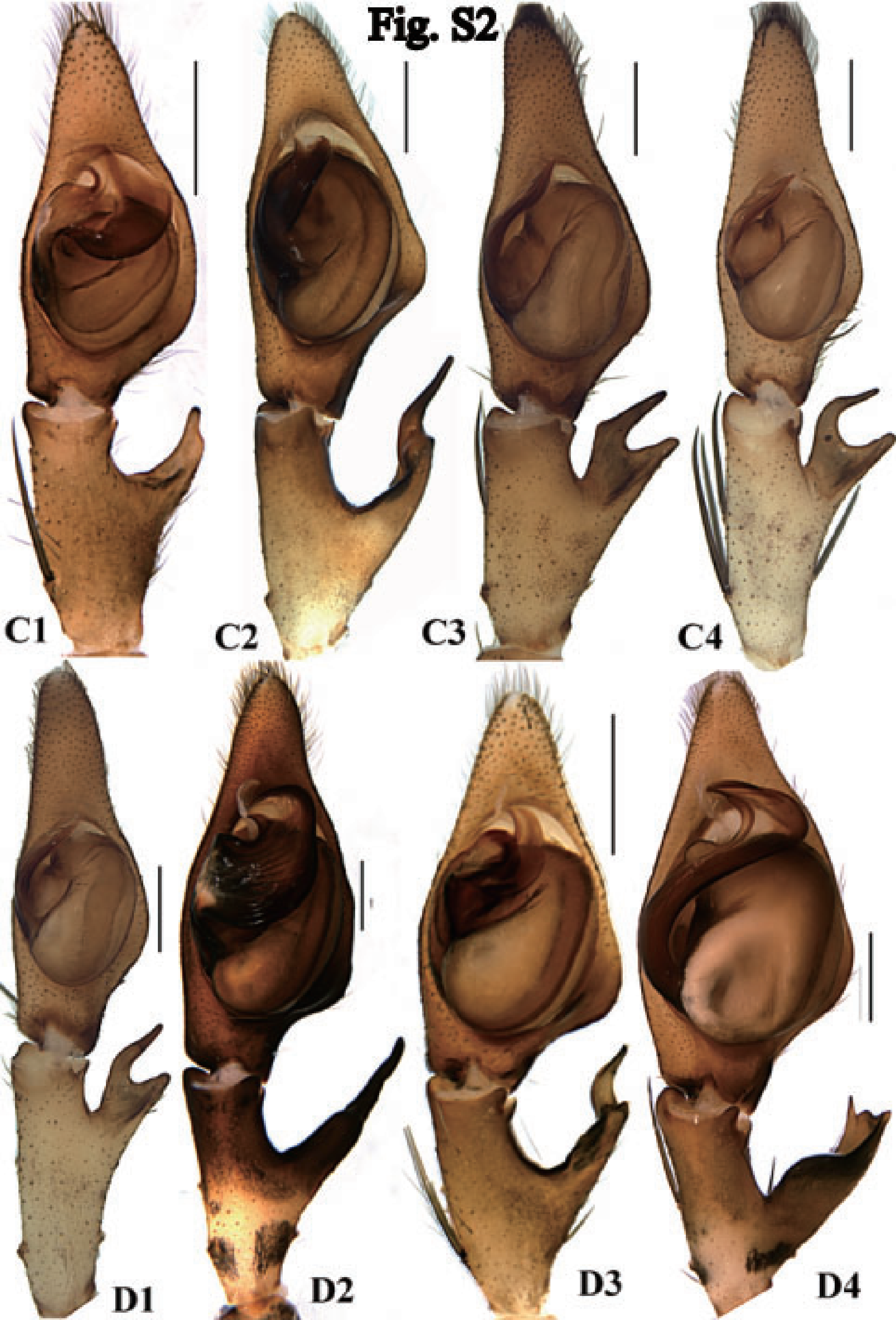

Fig. S2

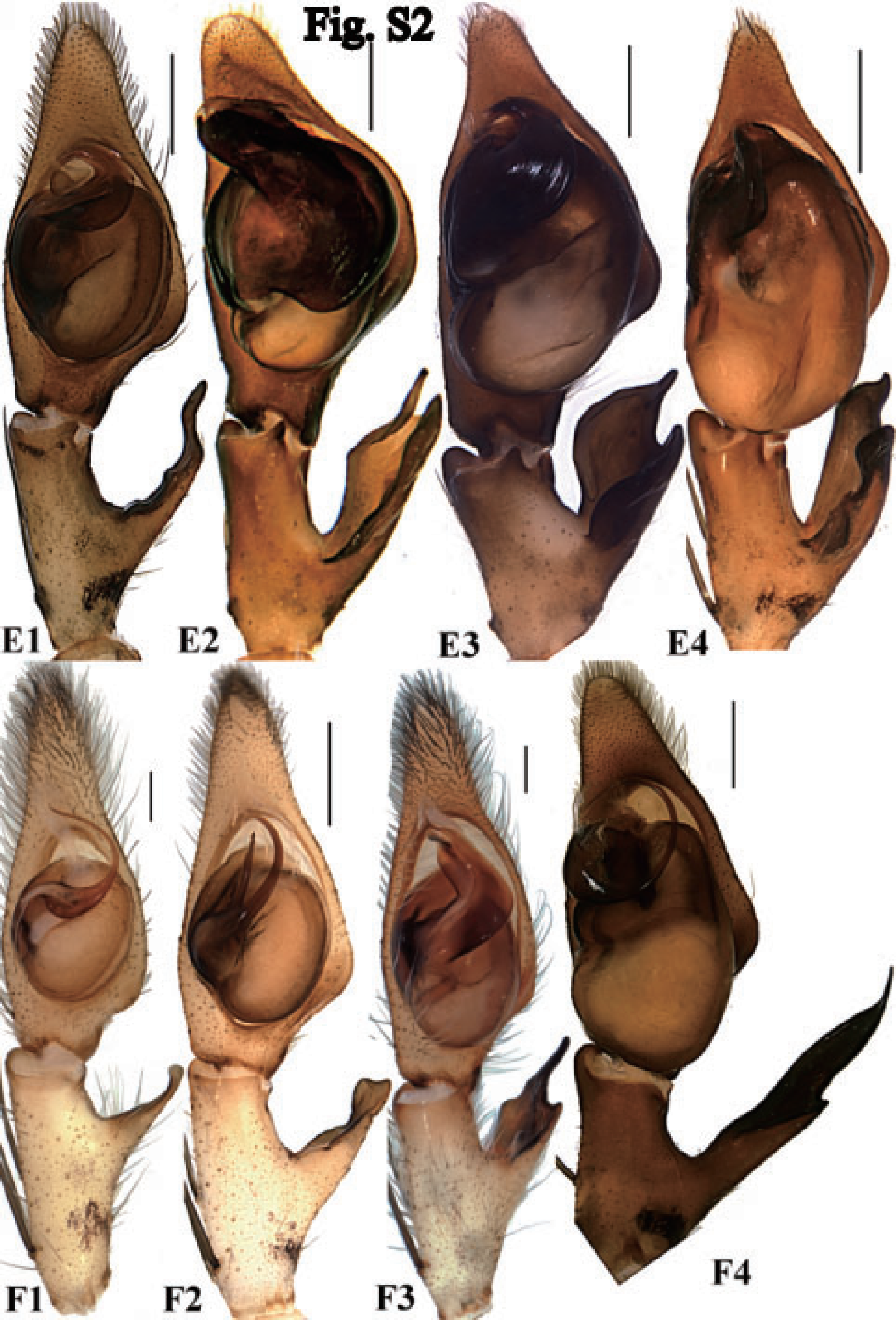

Fig. S2

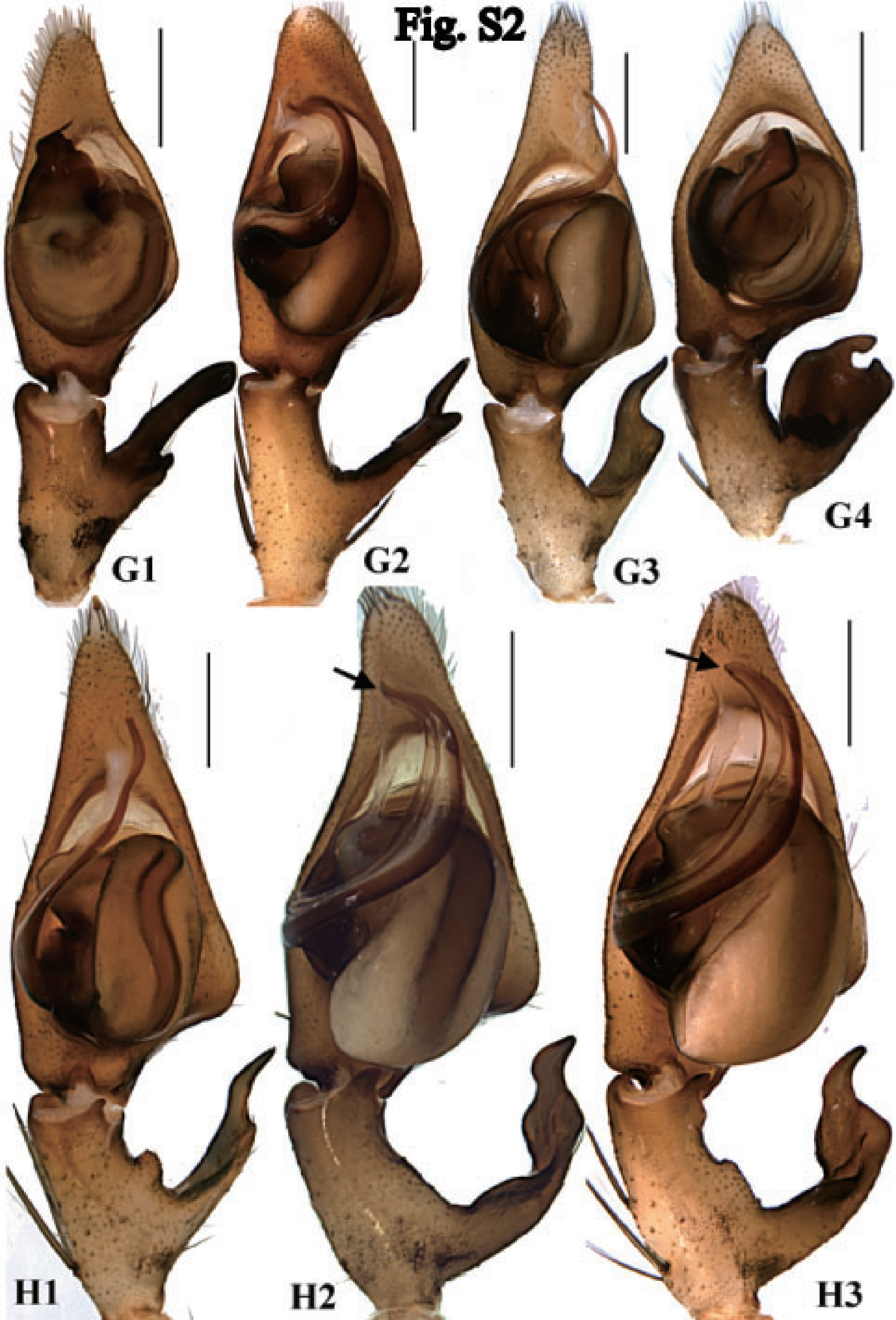

Fig. S2

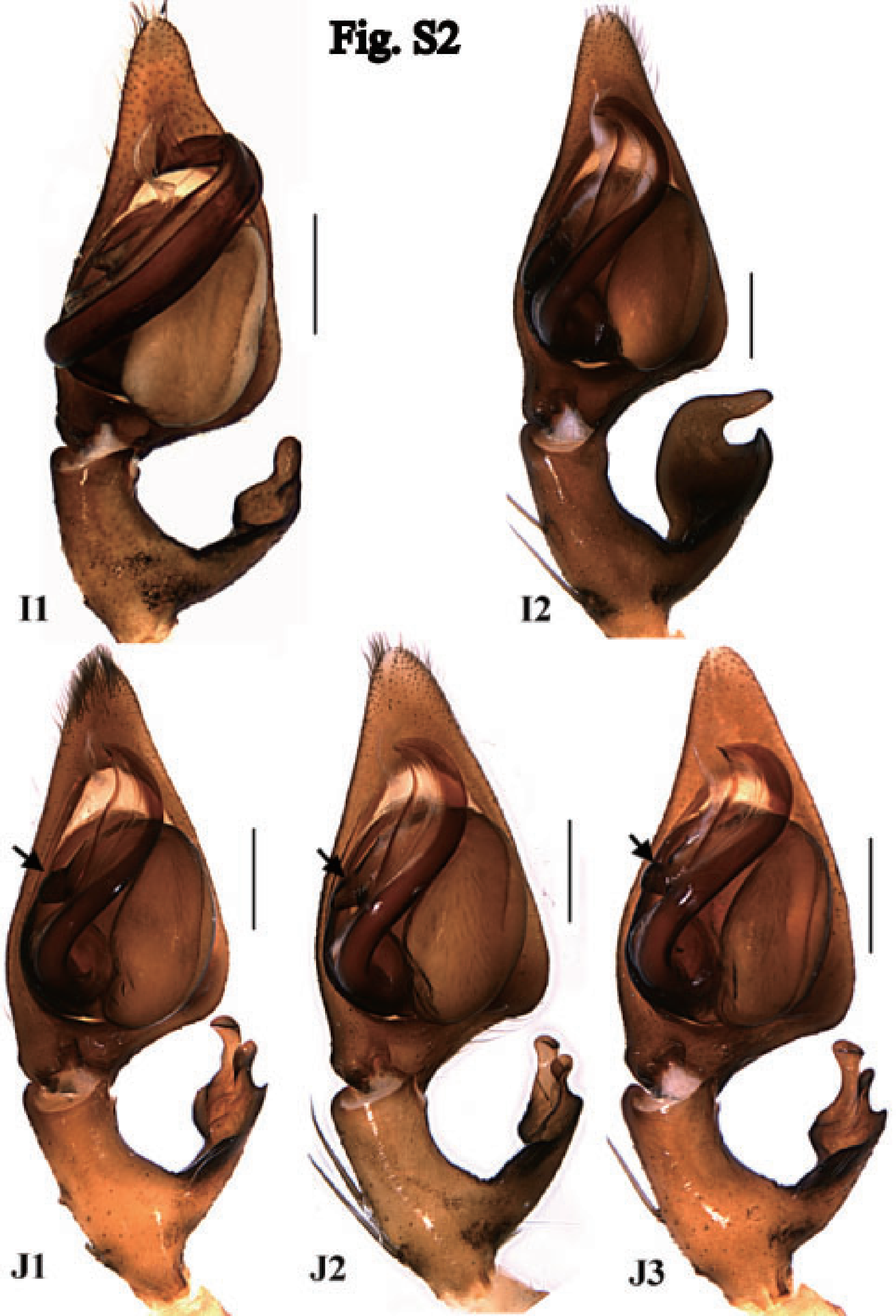

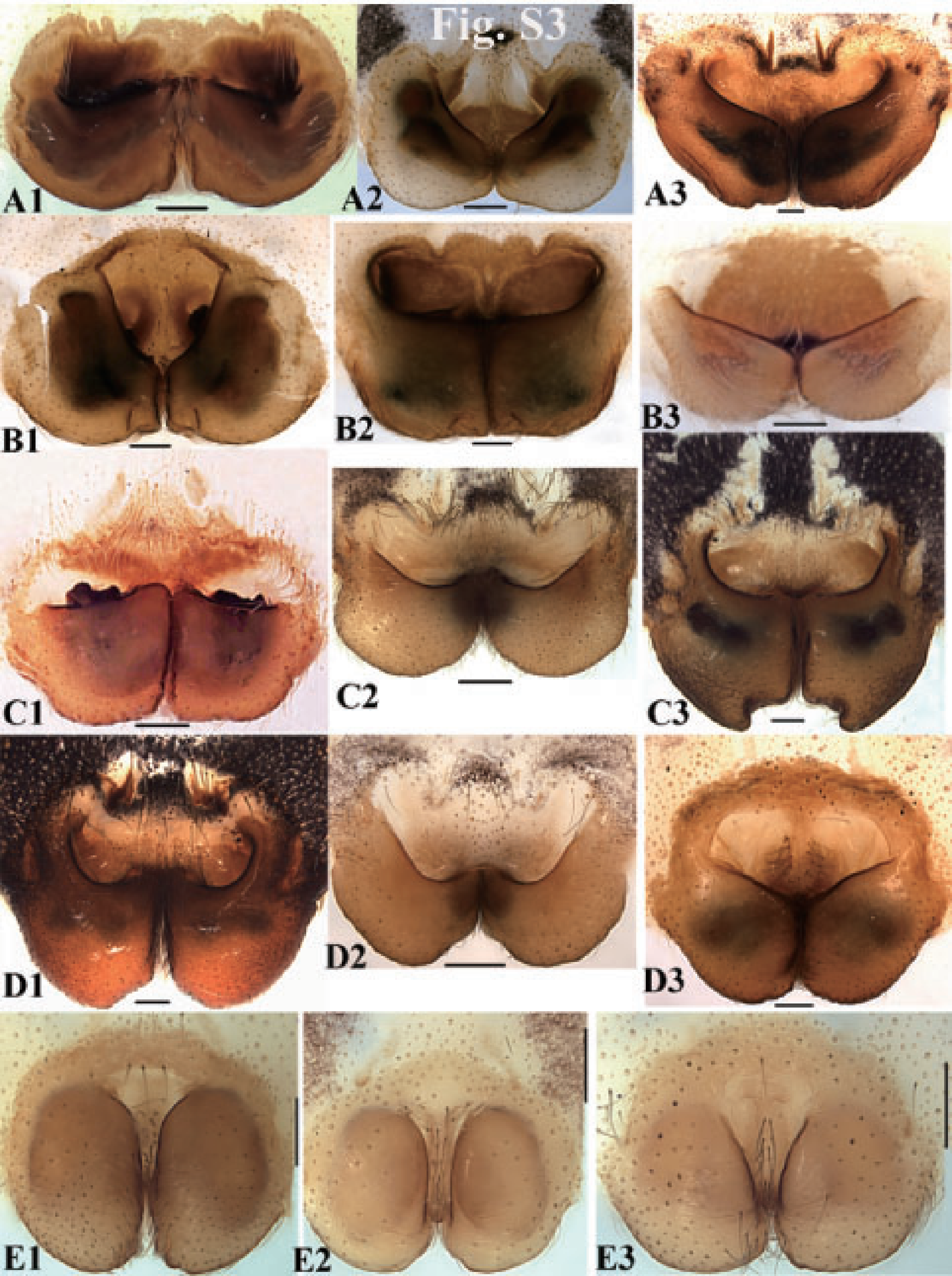
Fig. S3
A1
A2
A3
B1
B2
B3
C1
C2
C3
D1
D2
D3
E1
E2
E3

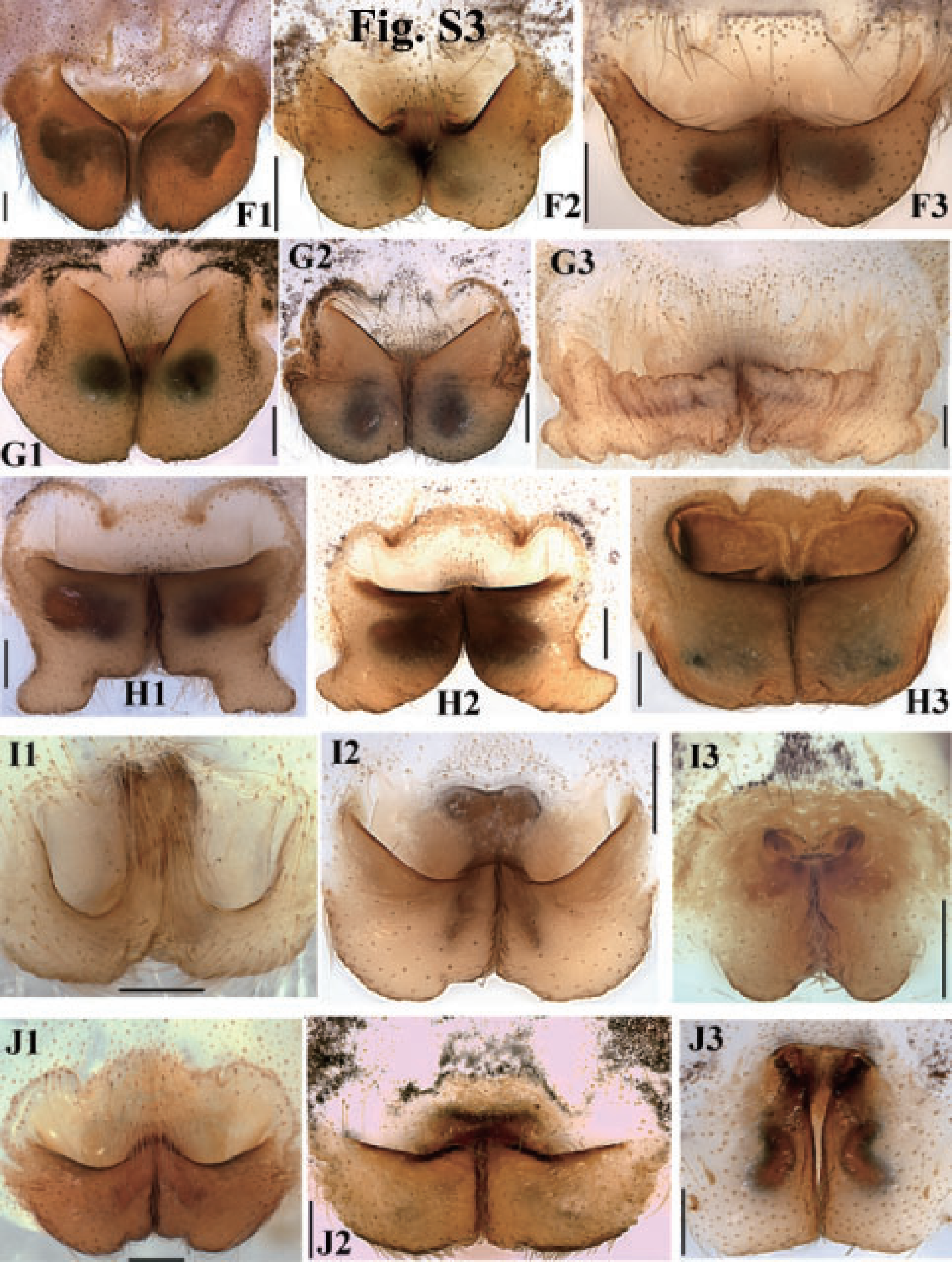
Fig. S3
F1
F2
F3
G1
G2
G3
H1
H2
H3
I1
I2
I3
J1
J2
J3

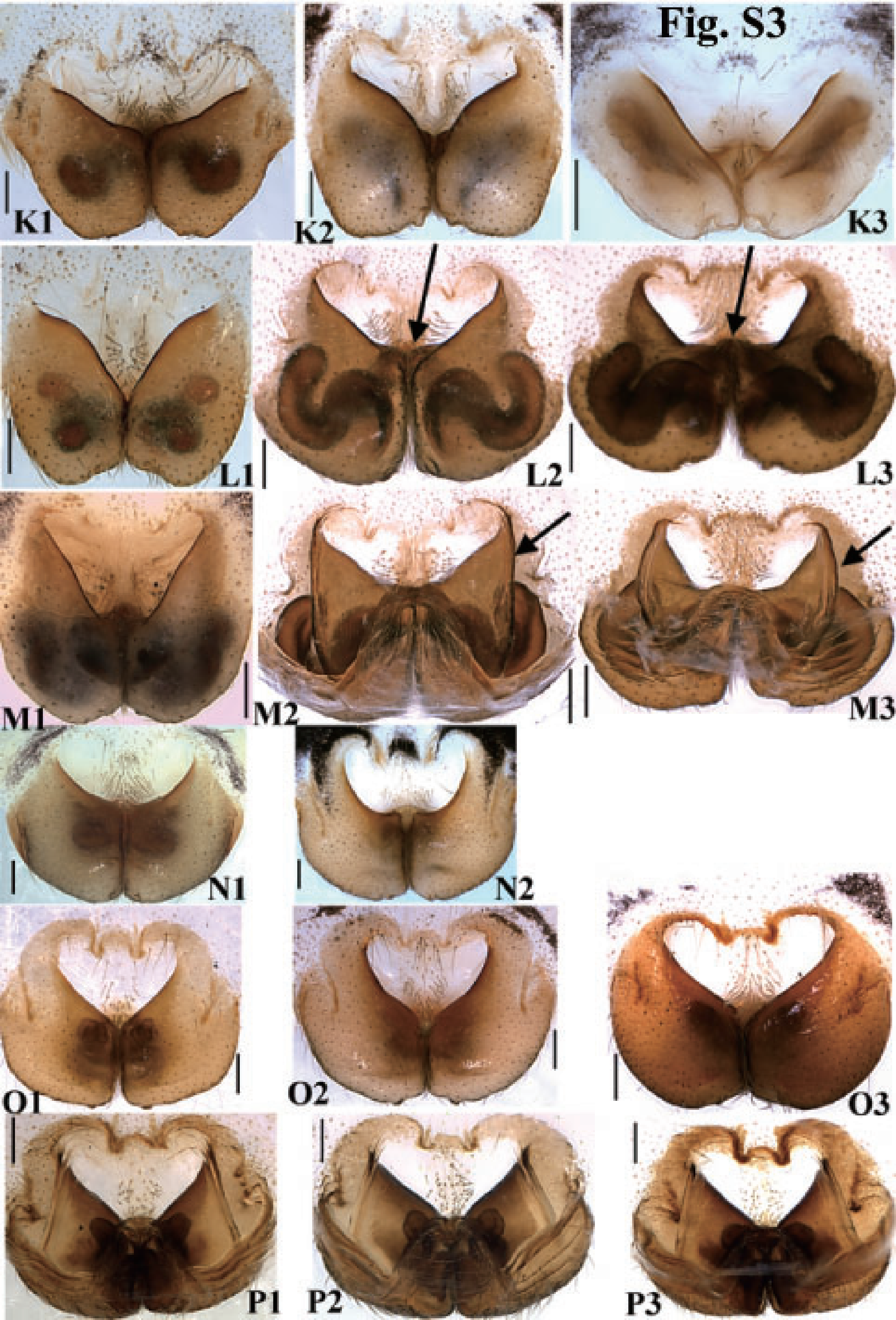
Fig. S3
K1
K2
K3
L1
L2
L3
M1
M2
M3
N1
N2
O1
O2
O3
P1
P2
P3

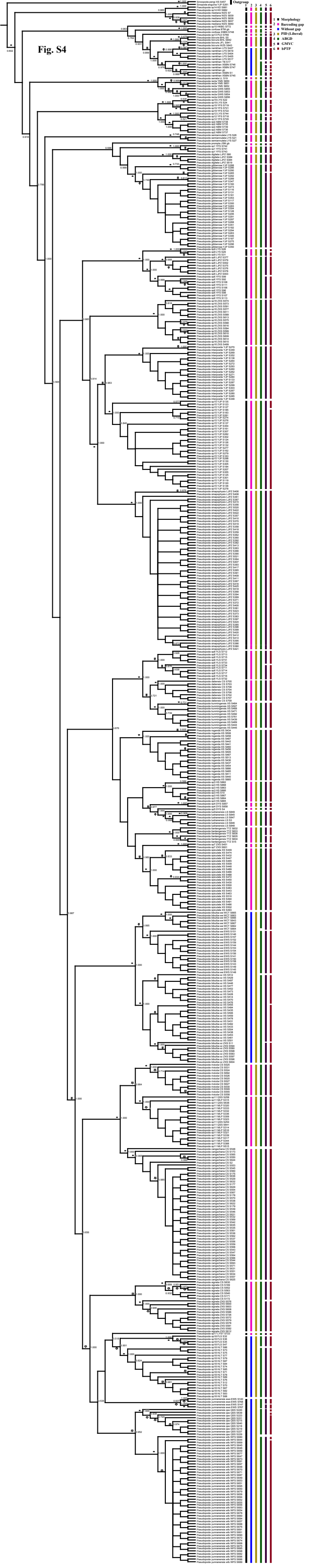

Fig. S4
Outgroup
1 2 3 4 5 6
1 Morphology
2 Barcoding gap
Without gap
3 PID (Liberal)
4 ABGD
5 GMYC
6 bPTP

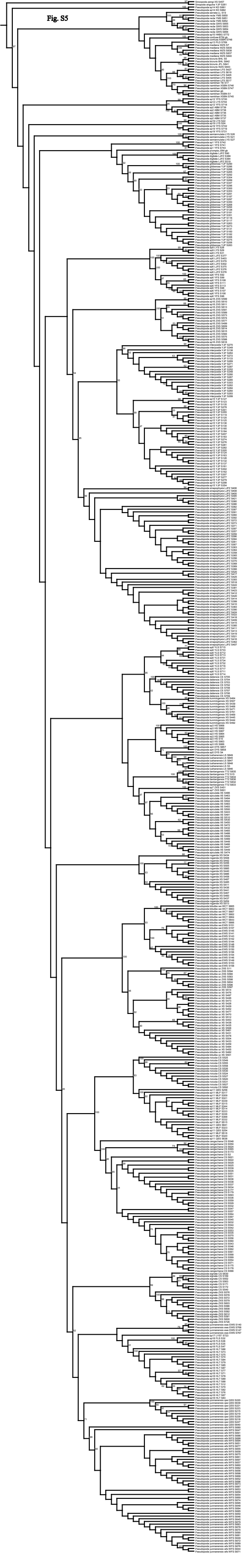

Fig. S5

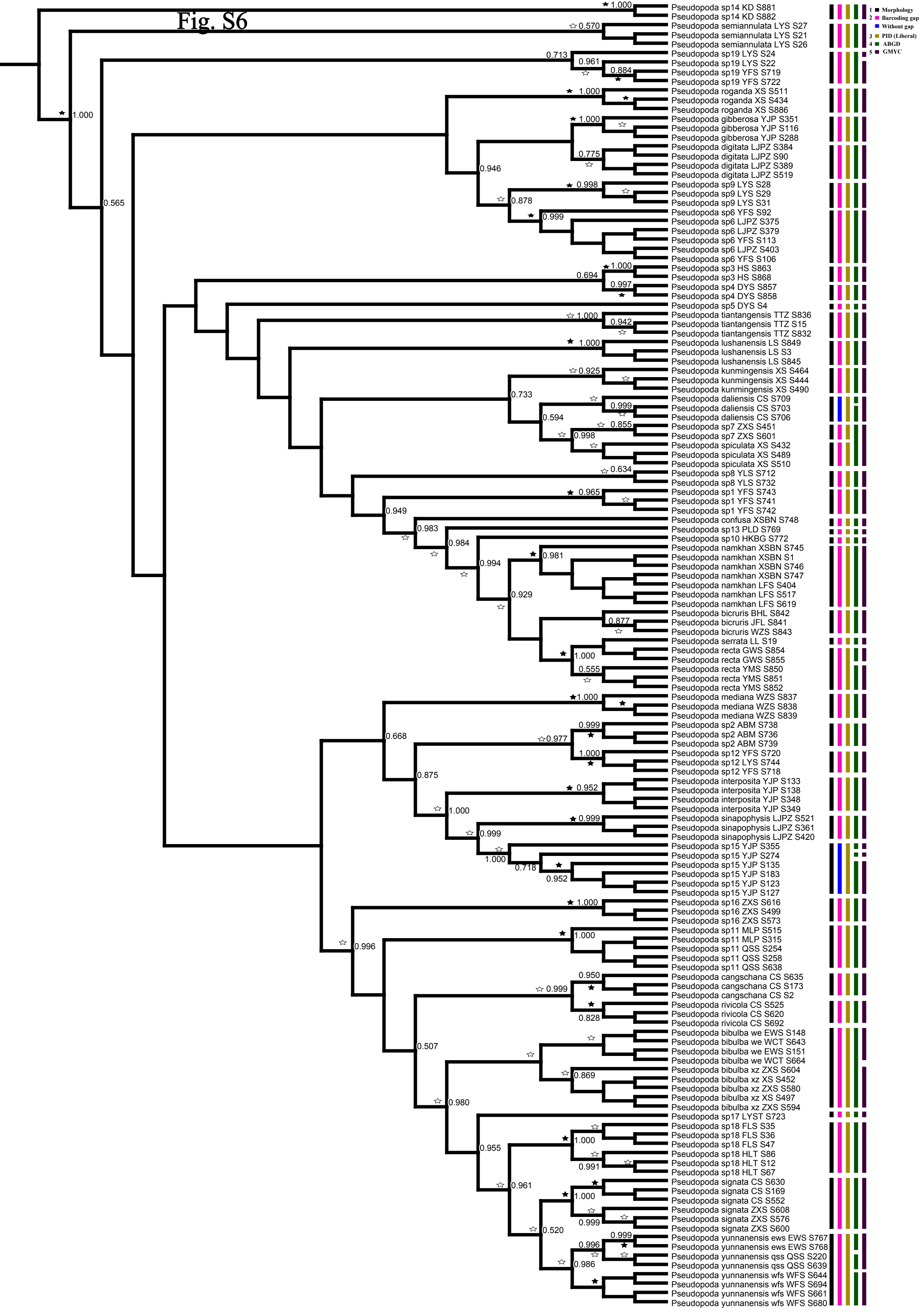
1 Morphology
2 Barcoding gap
Without gap
3 PID (Liberal)
4 ABGD
5 GMYC
Pseudopoda sp14 KD S881
Pseudopoda sp14 KD S882
Pseudopoda semiannulata LYS S27
Pseudopoda semiannulata LYS S21
Pseudopoda semiannulata LYS S26
Pseudopoda sp19 LYS S24
Pseudopoda sp19 LYS S22
Pseudopoda sp19 YFS S719
Pseudopoda sp19 YFS S722
Pseudopoda roganda XS S511
Pseudopoda roganda XS S434
Pseudopoda roganda XS S886
Pseudopoda gibberosa YJP S351
Pseudopoda gibberosa YJP S116
Pseudopoda gibberosa YJP S288
Pseudopoda digitata LJPZ S384
Pseudopoda digitata LJPZ S90
Pseudopoda digitata LJPZ S389
Pseudopoda digitata LJPZ S519
Pseudopoda sp9 LYS S28
Pseudopoda sp9 LYS S29
Pseudopoda sp9 LYS S31
Pseudopoda sp6 YFS S92
Pseudopoda sp6 LJPZ S375
Pseudopoda sp6 LJPZ S379
Pseudopoda sp6 YFS S113
Pseudopoda sp6 LJPZ S403
Pseudopoda sp6 YFS S106
Pseudopoda sp3 HS S863
Pseudopoda sp3 HS S868
Pseudopoda sp4 DYS S857
Pseudopoda sp4 DYS S858
Pseudopoda sp5 DYS S4
Pseudopoda tiantangensis TTZ S836
Pseudopoda tiantangensis TTZ S15
Pseudopoda tiantangensis TTZ S832
Pseudopoda lushanensis LS S849
Pseudopoda lushanensis LS S3
Pseudopoda lushanensis LS S845
Pseudopoda kunmingensis XS S464
Pseudopoda kunmingensis XS S444
Pseudopoda kunmingensis XS S490
Pseudopoda daliensis CS S709
Pseudopoda daliensis CS S703
Pseudopoda daliensis CS S706
Pseudopoda sp7 ZXS S451
Pseudopoda sp7 ZXS S601
Pseudopoda spiculata XS S432
Pseudopoda spiculata XS S489
Pseudopoda spiculata XS S510
Pseudopoda sp8 YLS S712
Pseudopoda sp8 YLS S732
Pseudopoda sp1 YFS S743
Pseudopoda sp1 YFS S741
Pseudopoda sp1 YFS S742
Pseudopoda confusa XSBN S748
Pseudopoda sp13 PLD S769
Pseudopoda sp10 HKBG S772
Pseudopoda namkhan XSBN S745
Pseudopoda namkhan XSBN S1
Pseudopoda namkhan XSBN S746
Pseudopoda namkhan XSBN S747
Pseudopoda namkhan LFS S404
Pseudopoda namkhan LFS S517
Pseudopoda namkhan LFS S619
Pseudopoda bicruris BHL S842
Pseudopoda bicruris JFL S841
Pseudopoda bicruris WZS S843
Pseudopoda serrata LL S19
Pseudopoda recta GWS S854
Pseudopoda recta GWS S855
Pseudopoda recta YMS S850
Pseudopoda recta YMS S851
Pseudopoda recta YMS S852
Pseudopoda mediana WZS S837
Pseudopoda mediana WZS S838
Pseudopoda mediana WZS S839
Pseudopoda sp2 ABM S738
Pseudopoda sp2 ABM S736
Pseudopoda sp2 ABM S739
Pseudopoda sp12 YFS S720
Pseudopoda sp12 LYS S744
Pseudopoda sp12 YFS S718
Pseudopoda interposita YJP S133
Pseudopoda interposita YJP S138
Pseudopoda interposita YJP S348
Pseudopoda interposita YJP S349
Pseudopoda sinapophysis LJPZ S521
Pseudopoda sinapophysis LJPZ S361
Pseudopoda sinapophysis LJPZ S420
Pseudopoda sp15 YJP S355
Pseudopoda sp15 YJP S274
Pseudopoda sp15 YJP S135
Pseudopoda sp15 YJP S183
Pseudopoda sp15 YJP S123
Pseudopoda sp15 YJP S127
Pseudopoda sp16 ZXS S616
Pseudopoda sp16 ZXS S499
Pseudopoda sp16 ZXS S573
Pseudopoda sp11 MLP S515
Pseudopoda sp11 MLP S315
Pseudopoda sp11 QSS S254
Pseudopoda sp11 QSS S258
Pseudopoda sp11 QSS S638
Pseudopoda cangschana CS S635
Pseudopoda cangschana CS S173
Pseudopoda cangschana CS S2
Pseudopoda rivicola CS S525
Pseudopoda rivicola CS S620
Pseudopoda rivicola CS S692
Pseudopoda bibulba we EWS S148
Pseudopoda bibulba we WCT S643
Pseudopoda bibulba we EWS S151
Pseudopoda bibulba we WCT S664
Pseudopoda bibulba xz ZXS S604
Pseudopoda bibulba xz XS S452
Pseudopoda bibulba xz ZXS S580
Pseudopoda bibulba xz XS S497
Pseudopoda bibulba xz ZXS S594
Pseudopoda sp17 LYST S723
Pseudopoda sp18 FLS S35
Pseudopoda sp18 FLS S36
Pseudopoda sp18 FLS S47
Pseudopoda sp18 HLT S86
Pseudopoda sp18 HLT S12
Pseudopoda sp18 HLT S67
Pseudopoda signata CS S630
Pseudopoda signata CS S169
Pseudopoda signata CS S552
Pseudopoda signata ZXS S608
Pseudopoda signata ZXS S576
Pseudopoda signata ZXS S600
Pseudopoda yunnanensis ews EWS S767
Pseudopoda yunnanensis ews EWS S768
Pseudopoda yunnanensis qss QSS S220
Pseudopoda yunnanensis qss QSS S639
Pseudopoda yunnanensis wfs WFS S644
Pseudopoda yunnanensis wfs WFS S694
Pseudopoda yunnanensis wfs WFS S661
Pseudopoda yunnanensis wfs WFS S680

Fig. S6

Fig. S7

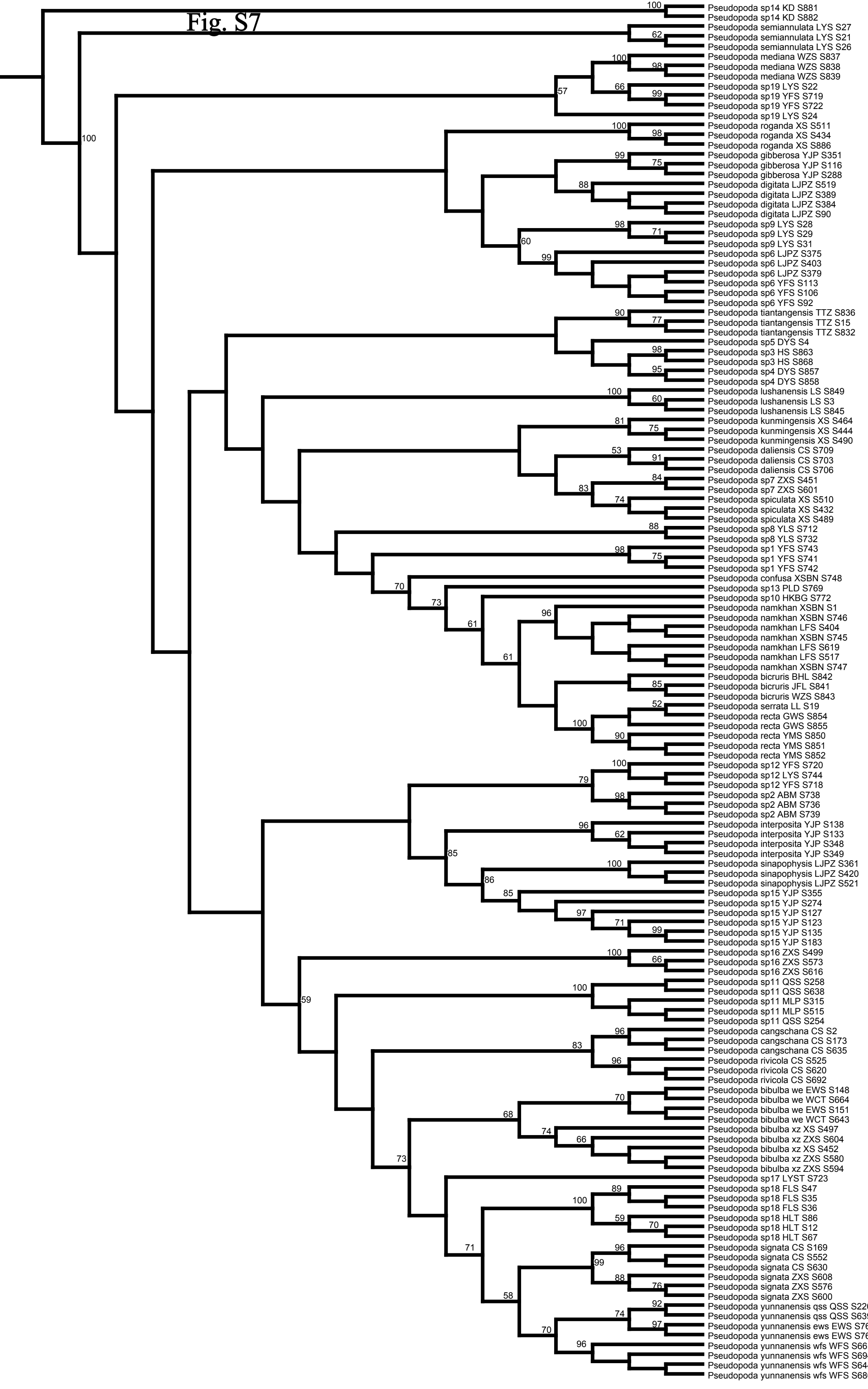
Pseudopoda sp14 KD S881
Pseudopoda sp14 KD S882
Pseudopoda semiannulata LYS S27
Pseudopoda semiannulata LYS S21
Pseudopoda semiannulata LYS S26
Pseudopoda mediana WZS S837
Pseudopoda mediana WZS S838
Pseudopoda mediana WZS S839
Pseudopoda sp19 LYS S22
Pseudopoda sp19 YFS S719
Pseudopoda sp19 YFS S722
Pseudopoda sp19 LYS S24
Pseudopoda roganda XS S511
Pseudopoda roganda XS S434
Pseudopoda roganda XS S886
Pseudopoda gibberosa YJP S351
Pseudopoda gibberosa YJP S116
Pseudopoda gibberosa YJP S288
Pseudopoda digitata LJPZ S519
Pseudopoda digitata LJPZ S389
Pseudopoda digitata LJPZ S384
Pseudopoda digitata LJPZ S90
Pseudopoda sp9 LYS S28
Pseudopoda sp9 LYS S29
Pseudopoda sp9 LYS S31
Pseudopoda sp6 LJPZ S375
Pseudopoda sp6 LJPZ S403
Pseudopoda sp6 LJPZ S379
Pseudopoda sp6 YFS S113
Pseudopoda sp6 YFS S106
Pseudopoda sp6 YFS S92
Pseudopoda tiantangensis TTZ S836
Pseudopoda tiantangensis TTZ S15
Pseudopoda tiantangensis TTZ S832
Pseudopoda sp5 DYS S4
Pseudopoda sp3 HS S863
Pseudopoda sp3 HS S868
Pseudopoda sp4 DYS S857
Pseudopoda sp4 DYS S858
Pseudopoda lushanensis LS S849
Pseudopoda lushanensis LS S3
Pseudopoda lushanensis LS S845
Pseudopoda kunmingensis XS S464
Pseudopoda kunmingensis XS S444
Pseudopoda kunmingensis XS S490
Pseudopoda daliensis CS S709
Pseudopoda daliensis CS S703
Pseudopoda daliensis CS S706
Pseudopoda sp7 ZXS S451
Pseudopoda sp7 ZXS S601
Pseudopoda spiculata XS S510
Pseudopoda spiculata XS S432
Pseudopoda spiculata XS S489
Pseudopoda sp8 YLS S712
Pseudopoda sp8 YLS S732
Pseudopoda sp1 YFS S743
Pseudopoda sp1 YFS S741
Pseudopoda sp1 YFS S742
Pseudopoda confusa XSBN S748
Pseudopoda sp13 PLD S769
Pseudopoda sp10 HKBG S772
Pseudopoda namkhan XSBN S1
Pseudopoda namkhan XSBN S746
Pseudopoda namkhan LFS S404
Pseudopoda namkhan XSBN S745
Pseudopoda namkhan LFS S619
Pseudopoda namkhan LFS S517
Pseudopoda namkhan XSBN S747
Pseudopoda bicruris BHL S842
Pseudopoda bicruris JFL S841
Pseudopoda bicruris WZS S843
Pseudopoda serrata LL S19
Pseudopoda recta GWS S854
Pseudopoda recta GWS S855
Pseudopoda recta YMS S850
Pseudopoda recta YMS S851
Pseudopoda recta YMS S852
Pseudopoda sp12 YFS S720
Pseudopoda sp12 LYS S744
Pseudopoda sp12 YFS S718
Pseudopoda sp2 ABM S738
Pseudopoda sp2 ABM S736
Pseudopoda sp2 ABM S739
Pseudopoda interposita YJP S138
Pseudopoda interposita YJP S133
Pseudopoda interposita YJP S348
Pseudopoda interposita YJP S349
Pseudopoda sinapophysis LJPZ S361
Pseudopoda sinapophysis LJPZ S420
Pseudopoda sinapophysis LJPZ S521
Pseudopoda sp15 YJP S355
Pseudopoda sp15 YJP S274
Pseudopoda sp15 YJP S127
Pseudopoda sp15 YJP S123
Pseudopoda sp15 YJP S135
Pseudopoda sp15 YJP S183
Pseudopoda sp16 ZXS S499
Pseudopoda sp16 ZXS S573
Pseudopoda sp16 ZXS S616
Pseudopoda sp11 QSS S258
Pseudopoda sp11 QSS S638
Pseudopoda sp11 MLP S315
Pseudopoda sp11 MLP S515
Pseudopoda sp11 QSS S254
Pseudopoda cangschana CS S2
Pseudopoda cangschana CS S173
Pseudopoda cangschana CS S635
Pseudopoda rivicola CS S525
Pseudopoda rivicola CS S620
Pseudopoda rivicola CS S692
Pseudopoda bibulba we EWS S148
Pseudopoda bibulba we WCT S664
Pseudopoda bibulba we EWS S151
Pseudopoda bibulba we WCT S643
Pseudopoda bibulba xz XS S497
Pseudopoda bibulba xz ZXS S604
Pseudopoda bibulba xz XS S452
Pseudopoda bibulba xz ZXS S580
Pseudopoda bibulba xz ZXS S594
Pseudopoda sp17 LYST S723
Pseudopoda sp18 FLS S47
Pseudopoda sp18 FLS S35
Pseudopoda sp18 FLS S36
Pseudopoda sp18 HLT S86
Pseudopoda sp18 HLT S12
Pseudopoda sp18 HLT S67
Pseudopoda signata CS S169
Pseudopoda signata CS S552
Pseudopoda signata CS S630
Pseudopoda signata ZXS S608
Pseudopoda signata ZXS S576
Pseudopoda signata ZXS S600
Pseudopoda yunnanensis qss QSS S22
Pseudopoda yunnanensis qss QSS S63
Pseudopoda yunnanensis ews EWS S7
Pseudopoda yunnanensis ews EWS S7
Pseudopoda yunnanensis wfs WFS S66
Pseudopoda yunnanensis wfs WFS S69
Pseudopoda yunnanensis wfs WFS S64
Pseudopoda yunnanensis wfs WFS S68

Fig. S8

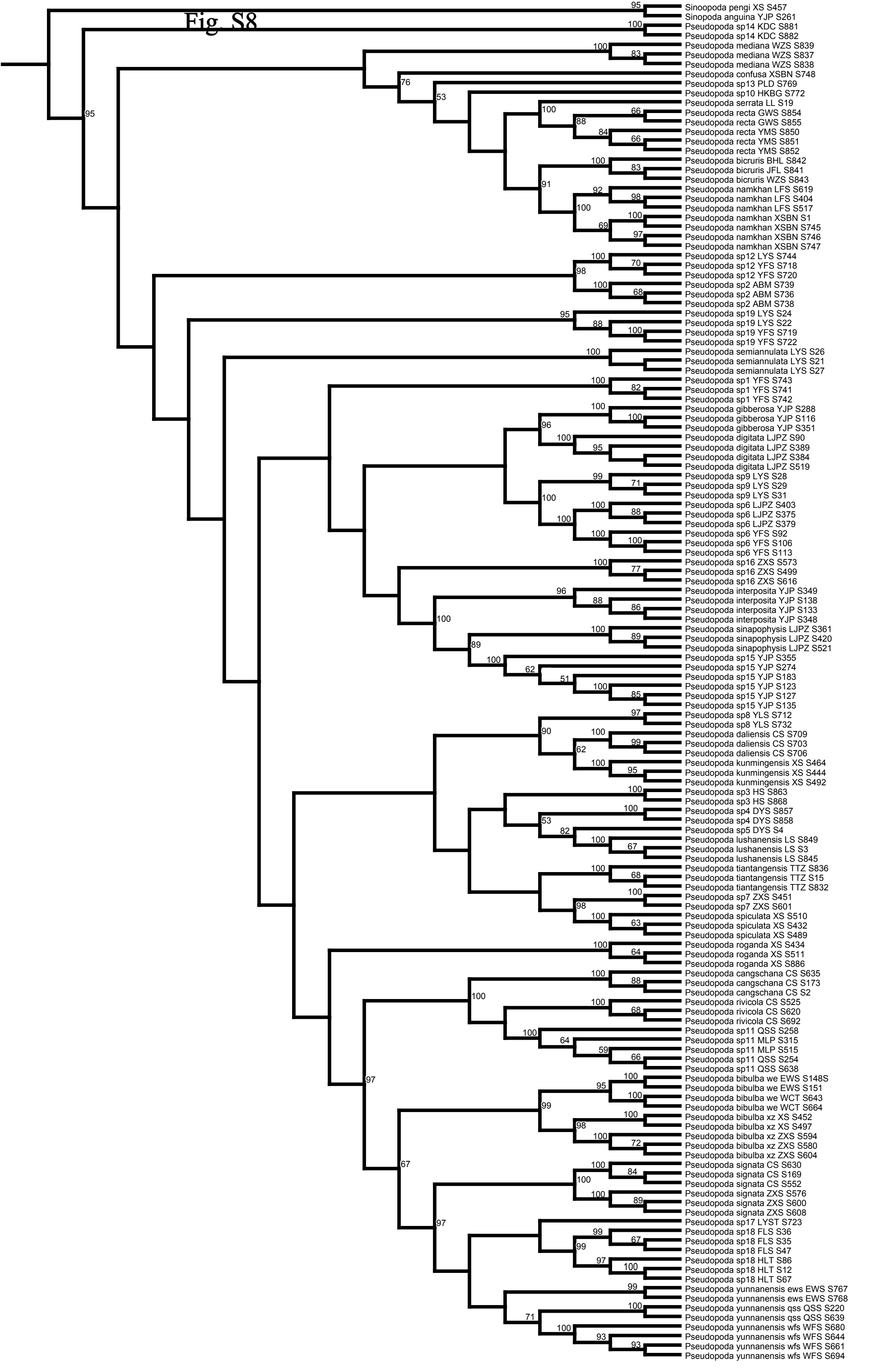
Sinoopoda pengi XS S457
Sinopoda anguina YJP S261
Pseudopoda sp14 KDC S881
Pseudopoda sp14 KDC S882
Pseudopoda mediana WZS S839
Pseudopoda mediana WZS S837
Pseudopoda mediana WZS S838
Pseudopoda confusa XSBN S748
Pseudopoda sp13 PLD S769
Pseudopoda sp10 HKBG S772
Pseudopoda serrata LL S19
Pseudopoda recta GWS S854
Pseudopoda recta GWS S855
Pseudopoda recta YMS S850
Pseudopoda recta YMS S851
Pseudopoda recta YMS S852
Pseudopoda bicruris BHL S842
Pseudopoda bicruris JFL S841
Pseudopoda bicruris WZS S843
Pseudopoda namkhan LFS S619
Pseudopoda namkhan LFS S404
Pseudopoda namkhan LFS S517
Pseudopoda namkhan XSBN S1
Pseudopoda namkhan XSBN S745
Pseudopoda namkhan XSBN S746
Pseudopoda namkhan XSBN S747
Pseudopoda sp12 LYS S744
Pseudopoda sp12 YFS S718
Pseudopoda sp12 YFS S720
Pseudopoda sp2 ABM S739
Pseudopoda sp2 ABM S736
Pseudopoda sp2 ABM S738
Pseudopoda sp19 LYS S24
Pseudopoda sp19 LYS S22
Pseudopoda sp19 YFS S719
Pseudopoda sp19 YFS S722
Pseudopoda semiannulata LYS S26
Pseudopoda semiannulata LYS S21
Pseudopoda semiannulata LYS S27
Pseudopoda sp1 YFS S743
Pseudopoda sp1 YFS S741
Pseudopoda sp1 YFS S742
Pseudopoda gibberosa YJP S288
Pseudopoda gibberosa YJP S116
Pseudopoda gibberosa YJP S351
Pseudopoda digitata LJPZ S90
Pseudopoda digitata LJPZ S389
Pseudopoda digitata LJPZ S384
Pseudopoda digitata LJPZ S519
Pseudopoda sp9 LYS S28
Pseudopoda sp9 LYS S29
Pseudopoda sp9 LYS S31
Pseudopoda sp6 LJPZ S403
Pseudopoda sp6 LJPZ S375
Pseudopoda sp6 LJPZ S379
Pseudopoda sp6 YFS S92
Pseudopoda sp6 YFS S106
Pseudopoda sp6 YFS S113
Pseudopoda sp16 ZXS S573
Pseudopoda sp16 ZXS S499
Pseudopoda sp16 ZXS S616
Pseudopoda interposita YJP S349
Pseudopoda interposita YJP S138
Pseudopoda interposita YJP S133
Pseudopoda interposita YJP S348
Pseudopoda sinapophysis LJPZ S361
Pseudopoda sinapophysis LJPZ S420
Pseudopoda sinapophysis LJPZ S521
Pseudopoda sp15 YJP S355
Pseudopoda sp15 YJP S274
Pseudopoda sp15 YJP S183
Pseudopoda sp15 YJP S123
Pseudopoda sp15 YJP S127
Pseudopoda sp15 YJP S135
Pseudopoda sp8 YLS S712
Pseudopoda sp8 YLS S732
Pseudopoda daliensis CS S709
Pseudopoda daliensis CS S703
Pseudopoda daliensis CS S706
Pseudopoda kunmingensis XS S464
Pseudopoda kunmingensis XS S444
Pseudopoda kunmingensis XS S492
Pseudopoda sp3 HS S863
Pseudopoda sp3 HS S868
Pseudopoda sp4 DYS S857
Pseudopoda sp4 DYS S858
Pseudopoda sp5 DYS S4
Pseudopoda lushanensis LS S849
Pseudopoda lushanensis LS S3
Pseudopoda lushanensis LS S845
Pseudopoda tiantangensis TTZ S836
Pseudopoda tiantangensis TTZ S15
Pseudopoda tiantangensis TTZ S832
Pseudopoda sp7 ZXS S451
Pseudopoda sp7 ZXS S601
Pseudopoda spiculata XS S510
Pseudopoda spiculata XS S432
Pseudopoda spiculata XS S489
Pseudopoda roganda XS S434
Pseudopoda roganda XS S511
Pseudopoda roganda XS S886
Pseudopoda cangschana CS S635
Pseudopoda cangschana CS S173
Pseudopoda cangschana CS S2
Pseudopoda rivicola CS S525
Pseudopoda rivicola CS S620
Pseudopoda rivicola CS S692
Pseudopoda sp11 QSS S258
Pseudopoda sp11 MLP S315
Pseudopoda sp11 MLP S515
Pseudopoda sp11 QSS S254
Pseudopoda sp11 QSS S638
Pseudopoda bibulba we EWS S148S
Pseudopoda bibulba we EWS S151
Pseudopoda bibulba we WCT S643
Pseudopoda bibulba we WCT S664
Pseudopoda bibulba xz XS S452
Pseudopoda bibulba xz XS S497
Pseudopoda bibulba xz ZXS S594
Pseudopoda bibulba xz ZXS S580
Pseudopoda bibulba xz ZXS S604
Pseudopoda signata CS S630
Pseudopoda signata CS S169
Pseudopoda signata CS S552
Pseudopoda signata ZXS S576
Pseudopoda signata ZXS S600
Pseudopoda signata ZXS S608
Pseudopoda sp17 LYST S723
Pseudopoda sp18 FLS S36
Pseudopoda sp18 FLS S35
Pseudopoda sp18 FLS S47
Pseudopoda sp18 HLT S86
Pseudopoda sp18 HLT S12
Pseudopoda sp18 HLT S67
Pseudopoda yunnanensis ews EWS S767
Pseudopoda yunnanensis ews EWS S768
Pseudopoda yunnanensis qss QSS S220
Pseudopoda yunnanensis qss QSS S639
Pseudopoda yunnanensis wfs WFS S680
Pseudopoda yunnanensis wfs WFS S644
Pseudopoda yunnanensis wfs WFS S661
Pseudopoda yunnanensis wfs WFS S694

Table S1. List of voucher specimens sequenced for this study, collection data and Genbank accession numbers. All species belongs to Sparassidae. Sparassidae genera and species are listed in alphabetical order.

Specimen code	Species	Site abbreviation	Province, Country	Locality	Elevati	Genbank COI	ITS2
S141	*Pseudopoda bibulba we*	EWS	Yunnan, China	Erwushan Mountain	2381m	KY096077	
S143	*Pseudopoda bibulba we*	EWS	Yunnan, China	Erwushan Mountain	2344m	KY096078	
S144	*Pseudopoda bibulba we*	EWS	Yunnan, China	Erwushan Mountain	2377m	KY096079	
S145	*Pseudopoda bibulba we*	EWS	Yunnan, China	Erwushan Mountain	2377m	KY096080	
S146	*Pseudopoda bibulba we*	EWS	Yunnan, China	Erwushan Mountain	2377m	KY096081	
S148	*Pseudopoda bibulba we*	EWS	Yunnan, China	Erwushan Mountain	2377m	KY096082	KY095937
S149	*Pseudopoda bibulba we*	EWS	Yunnan, China	Erwushan Mountain	2377m	KY096083	
S150	*Pseudopoda bibulba we*	EWS	Yunnan, China	Erwushan Mountain	2377m	KY096084	
S151	*Pseudopoda bibulba we*	EWS	Yunnan, China	Erwushan Mountain	2369m	KY096085	KY095938
S152	*Pseudopoda bibulba we*	EWS	Yunnan, China	Erwushan Mountain	2369m	KY096086	
S153	*Pseudopoda bibulba we*	EWS	Yunnan, China	Erwushan Mountain	2369m	KY096087	
S154	*Pseudopoda bibulba we*	EWS	Yunnan, China	Erwushan Mountain	2369m	KY096088	
S156	*Pseudopoda bibulba we*	EWS	Yunnan, China	Erwushan Mountain	2376m	KY096089	
S157	*Pseudopoda bibulba we*	EWS	Yunnan, China	Erwushan Mountain	2376m	KY096090	
S158	*Pseudopoda bibulba we*	EWS	Yunnan, China	Erwushan Mountain	2376m	KY096091	
S159	*Pseudopoda bibulba we*	EWS	Yunnan, China	Erwushan Mountain	2376m	KY096092	
S643	*Pseudopoda bibulba we*	WCT	Yunnan, China	Wenchangta Tower	2375m	KY096093	KY095939
S662	*Pseudopoda bibulba we*	WCT	Yunnan, China	Wenchangta Tower	2301m	KY096094	
S663	*Pseudopoda bibulba we*	WCT	Yunnan, China	Wenchangta Tower	2288m	KY096095	
S664	*Pseudopoda bibulba we*	WCT	Yunnan, China	Wenchangta Tower	2313m	KY096096	KY095940
S665	*Pseudopoda bibulba we*	WCT	Yunnan, China	Wenchangta Tower	2294m	KY096097	
S666	*Pseudopoda bibulba we*	WCT	Yunnan, China	Wenchangta Tower	2342m	KY096098	
S667	*Pseudopoda bibulba we*	WCT	Yunnan, China	Wenchangta Tower	2375m	KY096099	
S11	*Pseudopoda bibulba xz*	ZXS	Yunnan, China	Zixishan Mountain	2474m	KY096100	
S428	*Pseudopoda bibulba xz*	XS	Yunnan, China	Xishan Mountain	2189m	KY096101	
S429	*Pseudopoda bibulba xz*	XS	Yunnan, China	Xishan Mountain	2189m	KY096102	
S431	*Pseudopoda bibulba xz*	XS	Yunnan, China	Xishan Mountain	2112m	KY096103	
S433	*Pseudopoda bibulba xz*	XS	Yunnan, China	Xishan Mountain	1975m	KY096104	
S435	*Pseudopoda bibulba xz*	XS	Yunnan, China	Xishan Mountain	2008m	KY096105	
S438	*Pseudopoda bibulba xz*	XS	Yunnan, China	Xishan Mountain	2025m	KY096106	
S446	*Pseudopoda bibulba xz*	XS	Yunnan, China	Xishan Mountain	2192m	KY096107	
S452	*Pseudopoda bibulba xz*	XS	Yunnan, China	Xishan Mountain	2179m	KY096108	KY095941
S453	*Pseudopoda bibulba xz*	XS	Yunnan, China	Xishan Mountain	2154m	KY096109	
S455	*Pseudopoda bibulba xz*	XS	Yunnan, China	Xishan Mountain	2038m	KY096110	
S459	*Pseudopoda bibulba xz*	XS	Yunnan, China	Xishan Mountain	1976m	KY096111	
S461	*Pseudopoda bibulba xz*	XS	Yunnan, China	Xishan Mountain	2035m	KY096112	
S470	*Pseudopoda bibulba xz*	XS	Yunnan, China	Xishan Mountain	2151m	KY096113	
S473	*Pseudopoda bibulba xz*	XS	Yunnan, China	Xishan Mountain	2237m	KY096114	
S476	*Pseudopoda bibulba xz*	XS	Yunnan, China	Xishan Mountain	2225m	KY096115	
S477	*Pseudopoda bibulba xz*	XS	Yunnan, China	Xishan Mountain	2155m	KY096116	
S478	*Pseudopoda bibulba xz*	XS	Yunnan, China	Xishan Mountain	2121m	KY096117	
S482	*Pseudopoda bibulba xz*	XS	Yunnan, China	Xishan Mountain	1976m	KY096118	
S484	*Pseudopoda bibulba xz*	XS	Yunnan, China	Xishan Mountain	2009m	KY096119	
S497	*Pseudopoda bibulba xz*	XS	Yunnan, China	Xishan Mountain	2262m	KY096120	KY095942
S501	*Pseudopoda bibulba xz*	XS	Yunnan, China	Xishan Mountain	2057m	KY096121	
S504	*Pseudopoda bibulba xz*	XS	Yunnan, China	Xishan Mountain	1943m	KY096122	
S506	*Pseudopoda bibulba xz*	XS	Yunnan, China	Xishan Mountain	2009m	KY096123	

S512	*Pseudopoda bibulba xz*	XS	Yunnan, China	Xishan Mountain	2249m	KY096124	
S514	*Pseudopoda bibulba xz*	XS	Yunnan, China	Xishan Mountain	2292m	KY096125	
S580	*Pseudopoda bibulba xz*	ZXS	Yunnan, China	Zixishan Mountain	2527m	KY096126	KY095943
S583	*Pseudopoda bibulba xz*	ZXS	Yunnan, China	Zixishan Mountain	2474m	KY096127	
S594	*Pseudopoda bibulba xz*	ZXS	Yunnan, China	Zixishan Mountain	2457m	KY096128	KY095944
S596	*Pseudopoda bibulba xz*	ZXS	Yunnan, China	Zixishan Mountain	2436m	KY096129	
S597	*Pseudopoda bibulba xz*	ZXS	Yunnan, China	Zixishan Mountain	2435m	KY096130	
S598	*Pseudopoda bibulba xz*	ZXS	Yunnan, China	Zixishan Mountain	2454m	KY096131	
S604	*Pseudopoda bibulba xz*	ZXS	Yunnan, China	Zixishan Mountain	2461m	KY096132	KY095945
S5	*Pseudopoda bicruris*	BHL	Hainan, China	Baihualing Mountain		KY096133	
S841	*Pseudopoda bicruris*	JFL	Hainan, China	Jianfengling Mountain		KY096135	KY095947
S842	*Pseudopoda bicruris*	BHL	Hainan, China	Baihualing Mountain		KY096134	KY095946
S843	*Pseudopoda bicruris*	WZS	Hainan, China	Wuzhishan Mountain	750m	KY096136	KY095948
S2	*Pseudopoda cangschana*	CS	Yunnan, China	Cangshan Mountain		KY096137	KY095949
S173	*Pseudopoda cangschana*	CS	Yunnan, China	Cangshan Mountain	2645m	KY096138	
S175	*Pseudopoda cangschana*	CS	Yunnan, China	Cangshan Mountain	2618m	KY096139	
S177	*Pseudopoda cangschana*	CS	Yunnan, China	Cangshan Mountain	2538m	KY096140	
S178	*Pseudopoda cangschana*	CS	Yunnan, China	Cangshan Mountain	2538m	KY096141	
S179	*Pseudopoda cangschana*	CS	Yunnan, China	Cangshan Mountain	2225m	KY096142	KY095950
S529	*Pseudopoda cangschana*	CS	Yunnan, China	Cangshan Mountain	2517m	KY096143	
S532	*Pseudopoda cangschana*	CS	Yunnan, China	Cangshan Mountain	2466m	KY096144	
S535	*Pseudopoda cangschana*	CS	Yunnan, China	Cangshan Mountain	2513m	KY096145	
S536	*Pseudopoda cangschana*	CS	Yunnan, China	Cangshan Mountain	2492m	KY096146	
S537	*Pseudopoda cangschana*	CS	Yunnan, China	Cangshan Mountain	2492m	KY096147	
S538	*Pseudopoda cangschana*	CS	Yunnan, China	Cangshan Mountain	2495m	KY096148	
S539	*Pseudopoda cangschana*	CS	Yunnan, China	Cangshan Mountain	2490m	KY096149	
S542	*Pseudopoda cangschana*	CS	Yunnan, China	Cangshan Mountain	2489m	KY096150	
S543	*Pseudopoda cangschana*	CS	Yunnan, China	Cangshan Mountain	2485m	KY096151	
S544	*Pseudopoda cangschana*	CS	Yunnan, China	Cangshan Mountain	2465m	KY096152	
S545	*Pseudopoda cangschana*	CS	Yunnan, China	Cangshan Mountain	2415m	KY096153	
S546	*Pseudopoda cangschana*	CS	Yunnan, China	Cangshan Mountain	2246m	KY096154	
S547	*Pseudopoda cangschana*	CS	Yunnan, China	Cangshan Mountain	2249m	KY096155	
S548	*Pseudopoda cangschana*	CS	Yunnan, China	Cangshan Mountain	2523m	KY096156	
S550	*Pseudopoda cangschana*	CS	Yunnan, China	Cangshan Mountain	2554m	KY096157	
S551	*Pseudopoda cangschana*	CS	Yunnan, China	Cangshan Mountain	2541m	KY096158	
S553	*Pseudopoda cangschana*	CS	Yunnan, China	Cangshan Mountain	2508m	KY096159	
S554	*Pseudopoda cangschana*	CS	Yunnan, China	Cangshan Mountain	2508m	KY096160	
S556	*Pseudopoda cangschana*	CS	Yunnan, China	Cangshan Mountain	2519m	KY096161	
S557	*Pseudopoda cangschana*	CS	Yunnan, China	Cangshan Mountain	2462m	KY096162	
S559	*Pseudopoda cangschana*	CS	Yunnan, China	Cangshan Mountain	2513m	KY096163	
S560	*Pseudopoda cangschana*	CS	Yunnan, China	Cangshan Mountain	2511m	KY096164	
S561	*Pseudopoda cangschana*	CS	Yunnan, China	Cangshan Mountain	2478m	KY096165	
S562	*Pseudopoda cangschana*	CS	Yunnan, China	Cangshan Mountain	2495m	KY096166	
S564	*Pseudopoda cangschana*	CS	Yunnan, China	Cangshan Mountain	2437m	KY096167	
S565	*Pseudopoda cangschana*	CS	Yunnan, China	Cangshan Mountain	2477m	KY096168	
S566	*Pseudopoda cangschana*	CS	Yunnan, China	Cangshan Mountain	2472m	KY096169	
S567	*Pseudopoda cangschana*	CS	Yunnan, China	Cangshan Mountain	2415m	KY096170	
S568	*Pseudopoda cangschana*	CS	Yunnan, China	Cangshan Mountain	2457m	KY096171	
S569	*Pseudopoda cangschana*	CS	Yunnan, China	Cangshan Mountain	2452m	KY096172	
S570	*Pseudopoda cangschana*	CS	Yunnan, China	Cangshan Mountain	2405m	KY096173	
S571	*Pseudopoda cangschana*	CS	Yunnan, China	Cangshan Mountain	2193m	KY096174	

S621	*Pseudopoda cangschana*	CS	Yunnan, China	Cangshan Mountain	2554m	KY096175	
S622	*Pseudopoda cangschana*	CS	Yunnan, China	Cangshan Mountain	2543m	KY096176	
S624	*Pseudopoda cangschana*	CS	Yunnan, China	Cangshan Mountain	2524m	KY096177	
S625	*Pseudopoda cangschana*	CS	Yunnan, China	Cangshan Mountain	2524m	KY096178	
S629	*Pseudopoda cangschana*	CS	Yunnan, China	Cangshan Mountain	2492m	KY096179	
S631	*Pseudopoda cangschana*	CS	Yunnan, China	Cangshan Mountain	2492m	KY096180	
S632	*Pseudopoda cangschana*	CS	Yunnan, China	Cangshan Mountain	2428m	KY096181	
S634	*Pseudopoda cangschana*	CS	Yunnan, China	Cangshan Mountain	2347m	KY096182	
S635	*Pseudopoda cangschana*	CS	Yunnan, China	Cangshan Mountain	2331m	KY096183	KY095951
S636	*Pseudopoda cangschana*	CS	Yunnan, China	Cangshan Mountain	2292m	KY096184	
S693	*Pseudopoda cangschana*	CS	Yunnan, China	Cangshan Mountain	2446m	KY096185	
S748	*Pseudopoda confusa*	XSBN	Yunnan, China	Xishuangbanna	706m	KY096186	KY095952
S702	*Pseudopoda daliensis*	CS	Yunnan, China	Cangshan Mountain	2421m	KY096187	
S703	*Pseudopoda daliensis*	CS	Yunnan, China	Cangshan Mountain	2641m	KY096188	KY095953
S704	*Pseudopoda daliensis*	CS	Yunnan, China	Cangshan Mountain	2645m	KY096189	
S705	*Pseudopoda daliensis*	CS	Yunnan, China	Cangshan Mountain	2645m	KY096190	
S706	*Pseudopoda daliensis*	CS	Yunnan, China	Cangshan Mountain	2645m	KY096191	KY095954
S707	*Pseudopoda daliensis*	CS	Yunnan, China	Cangshan Mountain	2645m	KY096192	
S708	*Pseudopoda daliensis*	CS	Yunnan, China	Cangshan Mountain	2645m	KY096193	
S709	*Pseudopoda daliensis*	CS	Yunnan, China	Cangshan Mountain	2633m	KY096194	KY095955
S90	*Pseudopoda digitata*	LJPZ	Yunnan, China	Linjiapuzi Protection Station	2627m	KY096485	KY096027
S384	*Pseudopoda digitata*	LJPZ	Yunnan, China	Linjiapuzi Protection Station	2642m	KY096486	KY096028
S389	*Pseudopoda digitata*	LJPZ	Yunnan, China	Linjiapuzi Protection Station	2630m	KY096487	KY096029
S519	*Pseudopoda digitata*	LJPZ	Yunnan, China	Linjiapuzi Protection Station	2642m	KY096488	KY096030
S116	*Pseudopoda gibberosa*	YJP	Yunnan, China	Yaojiaping Protection Station	2586m	KY096195	KY095956
S117	*Pseudopoda gibberosa*	YJP	Yunnan, China	Yaojiaping Protection Station	2586m	KY096196	
S128	*Pseudopoda gibberosa*	YJP	Yunnan, China	Yaojiaping Protection Station	2543m	KY096197	
S131	*Pseudopoda gibberosa*	YJP	Yunnan, China	Yaojiaping Protection Station	2549m	KY096198	
S160	*Pseudopoda gibberosa*	YJP	Yunnan, China	Yaojiaping Protection Station	2437m	KY096199	
S181	*Pseudopoda gibberosa*	YJP	Yunnan, China	Yaojiaping Protection Station	2586m	KY096200	
S182	*Pseudopoda gibberosa*	YJP	Yunnan, China	Yaojiaping Protection Station	2586m	KY096201	
S187	*Pseudopoda gibberosa*	YJP	Yunnan, China	Yaojiaping Protection Station	2527m	KY096202	
S206	*Pseudopoda gibberosa*	YJP	Yunnan, China	Yaojiaping Protection Station	2497m	KY096203	
S263	*Pseudopoda gibberosa*	YJP	Yunnan, China	Yaojiaping Protection Station	2587m	KY096204	
S265	*Pseudopoda gibberosa*	YJP	Yunnan, China	Yaojiaping Protection Station	2566m	KY096205	
S268	*Pseudopoda gibberosa*	YJP	Yunnan, China	Yaojiaping Protection Station	2588m	KY096206	
S273	*Pseudopoda gibberosa*	YJP	Yunnan, China	Yaojiaping Protection Station	2569m	KY096207	
S275	*Pseudopoda gibberosa*	YJP	Yunnan, China	Yaojiaping Protection Station	2570m	KY096208	
S283	*Pseudopoda gibberosa*	YJP	Yunnan, China	Yaojiaping Protection Station	2509m	KY096209	
S286	*Pseudopoda gibberosa*	YJP	Yunnan, China	Yaojiaping Protection Station	2587m	KY096210	
S288	*Pseudopoda gibberosa*	YJP	Yunnan, China	Yaojiaping Protection Station	2587m	KY096211	KY095957
S289	*Pseudopoda gibberosa*	YJP	Yunnan, China	Yaojiaping Protection Station	2587m	KY096212	
S290	*Pseudopoda gibberosa*	YJP	Yunnan, China	Yaojiaping Protection Station	2587m	KY096213	
S291	*Pseudopoda gibberosa*	YJP	Yunnan, China	Yaojiaping Protection Station	2649m	KY096214	
S292	*Pseudopoda gibberosa*	YJP	Yunnan, China	Yaojiaping Protection Station	2649m	KY096215	
S294	*Pseudopoda gibberosa*	YJP	Yunnan, China	Yaojiaping Protection Station	2588m	KY096216	
S296	*Pseudopoda gibberosa*	YJP	Yunnan, China	Yaojiaping Protection Station	2588m	KY096217	
S297	*Pseudopoda gibberosa*	YJP	Yunnan, China	Yaojiaping Protection Station	2569m	KY096218	
S300	*Pseudopoda gibberosa*	YJP	Yunnan, China	Yaojiaping Protection Station	2569m	KY096219	
S304	*Pseudopoda gibberosa*	YJP	Yunnan, China	Yaojiaping Protection Station	2509m	KY096220	
S306	*Pseudopoda gibberosa*	YJP	Yunnan, China	Yaojiaping Protection Station	2509m	KY096221	

S347	*Pseudopoda gibberosa*	YJP	Yunnan, China	Yaojiaping Protection Station	2587m	KY096222	
S350	*Pseudopoda gibberosa*	YJP	Yunnan, China	Yaojiaping Protection Station	2587m	KY096223	
S351	*Pseudopoda gibberosa*	YJP	Yunnan, China	Yaojiaping Protection Station	2569m	KY096224	KY095958
S353	*Pseudopoda gibberosa*	YJP	Yunnan, China	Yaojiaping Protection Station	2543m	KY096225	
S133	*Pseudopoda interposita*	YJP	Yunnan, China	Yaojiaping Protection Station	2536m	KY096226	KY095959
S138	*Pseudopoda interposita*	YJP	Yunnan, China	Yaojiaping Protection Station	2504m	KY096227	KY095960
S260	*Pseudopoda interposita*	YJP	Yunnan, China	Yaojiaping Protection Station	2587m	KY096228	
S262	*Pseudopoda interposita*	YJP	Yunnan, China	Yaojiaping Protection Station	2587m	KY096229	
S264	*Pseudopoda interposita*	YJP	Yunnan, China	Yaojiaping Protection Station	2587m	KY096230	
S267	*Pseudopoda interposita*	YJP	Yunnan, China	Yaojiaping Protection Station	2569m	KY096231	
S269	*Pseudopoda interposita*	YJP	Yunnan, China	Yaojiaping Protection Station	2588m	KY096232	
S270	*Pseudopoda interposita*	YJP	Yunnan, China	Yaojiaping Protection Station	2588m	KY096233	
S271	*Pseudopoda interposita*	YJP	Yunnan, China	Yaojiaping Protection Station	2569m	KY096234	
S272	*Pseudopoda interposita*	YJP	Yunnan, China	Yaojiaping Protection Station	2569m	KY096235	
S280	*Pseudopoda interposita*	YJP	Yunnan, China	Yaojiaping Protection Station	2509m	KY096236	
S284	*Pseudopoda interposita*	YJP	Yunnan, China	Yaojiaping Protection Station	2587m	KY096237	
S285	*Pseudopoda interposita*	YJP	Yunnan, China	Yaojiaping Protection Station	2587m	KY096238	
S287	*Pseudopoda interposita*	YJP	Yunnan, China	Yaojiaping Protection Station	2587m	KY096239	
S293	*Pseudopoda interposita*	YJP	Yunnan, China	Yaojiaping Protection Station	2649m	KY096240	
S299	*Pseudopoda interposita*	YJP	Yunnan, China	Yaojiaping Protection Station	2569m	KY096241	
S303	*Pseudopoda interposita*	YJP	Yunnan, China	Yaojiaping Protection Station	2509m	KY096242	
S348	*Pseudopoda interposita*	YJP	Yunnan, China	Yaojiaping Protection Station	2587m	KY096243	KY095961
S349	*Pseudopoda interposita*	YJP	Yunnan, China	Yaojiaping Protection Station	2587m	KY096244	KY095962
S352	*Pseudopoda interposita*	YJP	Yunnan, China	Yaojiaping Protection Station	2588m	KY096245	
S439	*Pseudopoda kunmingensis*	XS	Yunnan, China	Xishan Mountain	2045m	KY096246	
S444	*Pseudopoda kunmingensis*	XS	Yunnan, China	Xishan Mountain	2049m	KY096247	KY095963
S445	*Pseudopoda kunmingensis*	XS	Yunnan, China	Xishan Mountain	2154m	KY096248	
S464	*Pseudopoda kunmingensis*	XS	Yunnan, China	Xishan Mountain	2144m	KY096249	KY095964
S468	*Pseudopoda kunmingensis*	XS	Yunnan, China	Xishan Mountain	2144m	KY096250	
S469	*Pseudopoda kunmingensis*	XS	Yunnan, China	Xishan Mountain	2167m	KY096251	
S471	*Pseudopoda kunmingensis*	XS	Yunnan, China	Xishan Mountain	2138m	KY096252	
S492	*Pseudopoda kunmingensis*	XS	Yunnan, China	Xishan Mountain	2202m	KY096253	KY095965
S507	*Pseudopoda kunmingensis*	XS	Yunnan, China	Xishan Mountain	2025m	KY096254	
S761	*Pseudopoda kunmingensis*	XS	Yunnan, China	Xishan Mountain	2128m	KY096255	
S3	*Pseudopoda lushanensis*	LS	Jiangxi, China	Lushan Mountain		KY096256	KY095966
S845	*Pseudopoda lushanensis*	LS	Jiangxi, China	Lushan Mountain		KY096257	KY095967
S846	*Pseudopoda lushanensis*	LS	Jiangxi, China	Lushan Mountain		KY096258	
S847	*Pseudopoda lushanensis*	LS	Jiangxi, China	Lushan Mountain		KY096259	
S848	*Pseudopoda lushanensis*	LS	Jiangxi, China	Lushan Mountain		KY096260	
S849	*Pseudopoda lushanensis*	LS	Jiangxi, China	Lushan Mountain		KY096261	KY095968
S7	*Pseudopoda mediana*	WZS	Hainan, China	Wuzhishan Mountain	1602m	KY096262	
S837	*Pseudopoda mediana*	WZS	Hainan, China	Wuzhishan Mountain	1602m	KY096263	KY095969
S838	*Pseudopoda mediana*	WZS	Hainan, China	Wuzhishan Mountain	1602m	KY096264	KY095970
S839	*Pseudopoda mediana*	WZS	Hainan, China	Wuzhishan Mountain	1602m	KY096265	KY095971
S840	*Pseudopoda mediana*	WZS	Hainan, China	Wuzhishan Mountain	1602m	KY096266	
S17	*Pseudopoda namkhan*	XSBN	Yunnan, China	Xishuangbanna	706m	KY096273	KY095972
S404	*Pseudopoda namkhan*	LFS	Yunnan, China	Laifengshan Mountain	1906m	KY096272	
S405	*Pseudopoda namkhan*	LFS	Yunnan, China	Laifengshan Mountain	1905m	KY096267	KY095995
S427	*Pseudopoda namkhan*	LFS	Yunnan, China	Laifengshan Mountain	1803m	KY096268	
S517	*Pseudopoda namkhan*	LFS	Yunnan, China	Laifengshan Mountain	1925m	KY096269	
S619	*Pseudopoda namkhan*	LFS	Yunnan, China	Laifengshan Mountain	1912m	KY096270	KY095996

S745	*Pseudopoda namkhan*	XSBN	Yunnan, China	Xishuangbanna	706m	KY096271	KY095997
S746	*Pseudopoda namkhan*	XSBN	Yunnan, China	Xishuangbanna	706m	KY096274	KY095973
S747	*Pseudopoda namkhan*	XSBN	Yunnan, China	Xishuangbanna	706m	KY096275	KY095974
S1	*Pseudopoda namkhan*	TB	Yunnan, China	Taibao Park		KY096276	KY095975
S850	*Pseudopoda recta*	YMS	Taiwan, China	Yangmingshan Mountain		KY096372	KY095991
S851	*Pseudopoda recta*	YMS	Taiwan, China	Yangmingshan Mountain		KY096373	KY095992
S852	*Pseudopoda recta*	YMS	Taiwan, China	Yangmingshan Mountain		KY096374	KY095993
S853	*Pseudopoda recta*	GWS	Taiwan, China	Guanwushan Mountain		KY096368	
S854	*Pseudopoda recta*	GWS	Taiwan, China	Guanwushan Mountain		KY096369	KY095989
S855	*Pseudopoda recta*	GWS	Taiwan, China	Guanwushan Mountain		KY096370	KY095990
S856	*Pseudopoda recta*	GWS	Taiwan, China	Guanwushan Mountain		KY096371	
S524	*Pseudopoda rivicola*	CS	Yunnan, China	Cangshan Mountain	2523m	KY096277	
S525	*Pseudopoda rivicola*	CS	Yunnan, China	Cangshan Mountain	2554m	KY096278	KY095976
S526	*Pseudopoda rivicola*	CS	Yunnan, China	Cangshan Mountain	2554m	KY096279	
S527	*Pseudopoda rivicola*	CS	Yunnan, China	Cangshan Mountain	2554m	KY096280	
S530	*Pseudopoda rivicola*	CS	Yunnan, China	Cangshan Mountain	2540m	KY096281	
S531	*Pseudopoda rivicola*	CS	Yunnan, China	Cangshan Mountain	2540m	KY096282	
S549	*Pseudopoda rivicola*	CS	Yunnan, China	Cangshan Mountain	2523m	KY096283	
S555	*Pseudopoda rivicola*	CS	Yunnan, China	Cangshan Mountain	2489m	KY096284	
S558	*Pseudopoda rivicola*	CS	Yunnan, China	Cangshan Mountain	2469m	KY096285	
S620	*Pseudopoda rivicola*	CS	Yunnan, China	Cangshan Mountain	2523m	KY096286	KY095977
S627	*Pseudopoda rivicola*	CS	Yunnan, China	Cangshan Mountain	2508m	KY096287	
S692	*Pseudopoda rivicola*	CS	Yunnan, China	Cangshan Mountain	2519m	KY096288	KY095978
S434	*Pseudopoda roganda*	XS	Yunnan, China	Xishan Mountain	2004m	KY096289	KY095979
S436	*Pseudopoda roganda*	XS	Yunnan, China	Xishan Mountain	2009m	KY096290	
S437	*Pseudopoda roganda*	XS	Yunnan, China	Xishan Mountain	1988m	KY096291	
S440	*Pseudopoda roganda*	XS	Yunnan, China	Xishan Mountain	2075m	KY096292	
S441	*Pseudopoda roganda*	XS	Yunnan, China	Xishan Mountain	2074m	KY096293	
S442	*Pseudopoda roganda*	XS	Yunnan, China	Xishan Mountain	2165m	KY096294	
S454	*Pseudopoda roganda*	XS	Yunnan, China	Xishan Mountain	2057m	KY096295	
S456	*Pseudopoda roganda*	XS	Yunnan, China	Xishan Mountain	2025m	KY096296	
S458	*Pseudopoda roganda*	XS	Yunnan, China	Xishan Mountain	1944m	KY096297	
S460	*Pseudopoda roganda*	XS	Yunnan, China	Xishan Mountain	1988m	KY096298	
S467	*Pseudopoda roganda*	XS	Yunnan, China	Xishan Mountain	2144m	KY096299	
S485	*Pseudopoda roganda*	XS	Yunnan, China	Xishan Mountain	1967m	KY096300	
S487	*Pseudopoda roganda*	XS	Yunnan, China	Xishan Mountain	2075m	KY096301	
S505	*Pseudopoda roganda*	XS	Yunnan, China	Xishan Mountain	2009m	KY096302	
S508	*Pseudopoda roganda*	XS	Yunnan, China	Xishan Mountain	2017m	KY096303	
S511	*Pseudopoda roganda*	XS	Yunnan, China	Xishan Mountain	2206m	KY096304	KY095980
S513	*Pseudopoda roganda*	XS	Yunnan, China	Xishan Mountain	2244m	KY096305	
S885	*Pseudopoda roganda*	XS	Yunnan, China	Xishan Mountain	2008m	KY096306	
S886	*Pseudopoda roganda*	XS	Yunnan, China	Xishan Mountain	2008m	KY096307	KY095981
S21	*Pseudopoda semiannulata*	LYS	Yunnan, China	Langyashan Mountain	2024m	KY096074	KY095934
S26	*Pseudopoda semiannulata*	LYS	Yunnan, China	Langyashan Mountain	2059m	KY096075	KY095935
S27	*Pseudopoda semiannulata*	LYS	Yunnan, China	Langyashan Mountain	2068m	KY096076	KY095936
S19	*Pseudopoda serrata*	LL	Taiwan, China	Loulan Forest Park		KY096553	KY096063
S169	*Pseudopoda signata*	CS	Yunnan, China	Cangshan Mountain	2645m	KY096463	KY096036
S171	*Pseudopoda signata*	CS	Yunnan, China	Cangshan Mountain	2645m	KY096464	
S172	*Pseudopoda signata*	CS	Yunnan, China	Cangshan Mountain	2645m	KY096465	
S540	*Pseudopoda signata*	CS	Yunnan, China	Cangshan Mountain	2469m	KY096466	
S552	*Pseudopoda signata*	CS	Yunnan, China	Cangshan Mountain	2524m	KY096467	KY096037

S563	*Pseudopoda signata*	CS	Yunnan, China	Cangshan Mountain	2489m	KY096468	
S572	*Pseudopoda signata*	ZXS	Yunnan, China	Zixishan Mountain	2479m	KY096470	
S576	*Pseudopoda signata*	ZXS	Yunnan, China	Zixishan Mountain	2469m	KY096471	KY096039
S578	*Pseudopoda signata*	ZXS	Yunnan, China	Zixishan Mountain	2498m	KY096472	
S579	*Pseudopoda signata*	ZXS	Yunnan, China	Zixishan Mountain	2527m	KY096473	
S581	*Pseudopoda signata*	ZXS	Yunnan, China	Zixishan Mountain	2499m	KY096474	
S582	*Pseudopoda signata*	ZXS	Yunnan, China	Zixishan Mountain	2474m	KY096475	
S586	*Pseudopoda signata*	ZXS	Yunnan, China	Zixishan Mountain	2480m	KY096476	
S600	*Pseudopoda signata*	ZXS	Yunnan, China	Zixishan Mountain	2465m	KY096477	KY096040
S603	*Pseudopoda signata*	ZXS	Yunnan, China	Zixishan Mountain	2461m	KY096478	
S608	*Pseudopoda signata*	ZXS	Yunnan, China	Zixishan Mountain	2474m	KY096479	KY096041
S612	*Pseudopoda signata*	ZXS	Yunnan, China	Zixishan Mountain	2476m	KY096480	
S630	*Pseudopoda signata*	CS	Yunnan, China	Cangshan Mountain	2510m	KY096469	KY096038
S726	*Pseudopoda signata*	ZXS	Yunnan, China	Zixishan Mountain	2465m	KY096481	
S356	*Pseudopoda sinapophysis*	LJPZ	Yunnan, China	Linjiapuzi Protection Station	2642m	KY096308	
S357	*Pseudopoda sinapophysis*	LJPZ	Yunnan, China	Linjiapuzi Protection Station	2626m	KY096309	
S358	*Pseudopoda sinapophysis*	LJPZ	Yunnan, China	Linjiapuzi Protection Station	2630m	KY096310	
S359	*Pseudopoda sinapophysis*	LJPZ	Yunnan, China	Linjiapuzi Protection Station	2630m	KY096311	
S360	*Pseudopoda sinapophysis*	LJPZ	Yunnan, China	Linjiapuzi Protection Station	2630m	KY096312	
S361	*Pseudopoda sinapophysis*	LJPZ	Yunnan, China	Linjiapuzi Protection Station	2630m	KY096313	KY095982
S362	*Pseudopoda sinapophysis*	LJPZ	Yunnan, China	Linjiapuzi Protection Station	2617m	KY096314	
S363	*Pseudopoda sinapophysis*	LJPZ	Yunnan, China	Linjiapuzi Protection Station	2598m	KY096315	
S364	*Pseudopoda sinapophysis*	LJPZ	Yunnan, China	Linjiapuzi Protection Station	2603m	KY096316	
S365	*Pseudopoda sinapophysis*	LJPZ	Yunnan, China	Linjiapuzi Protection Station	2607m	KY096317	
S366	*Pseudopoda sinapophysis*	LJPZ	Yunnan, China	Linjiapuzi Protection Station	2564m	KY096318	
S367	*Pseudopoda sinapophysis*	LJPZ	Yunnan, China	Linjiapuzi Protection Station	2494m	KY096319	
S368	*Pseudopoda sinapophysis*	LJPZ	Yunnan, China	Linjiapuzi Protection Station	2494m	KY096320	
S369	*Pseudopoda sinapophysis*	LJPZ	Yunnan, China	Linjiapuzi Protection Station	2494m	KY096321	
S370	*Pseudopoda sinapophysis*	LJPZ	Yunnan, China	Linjiapuzi Protection Station	2465m	KY096322	
S371	*Pseudopoda sinapophysis*	LJPZ	Yunnan, China	Linjiapuzi Protection Station	2452m	KY096323	
S372	*Pseudopoda sinapophysis*	LJPZ	Yunnan, China	Linjiapuzi Protection Station	2452m	KY096324	
S373	*Pseudopoda sinapophysis*	LJPZ	Yunnan, China	Linjiapuzi Protection Station	2441m	KY096325	
S380	*Pseudopoda sinapophysis*	LJPZ	Yunnan, China	Linjiapuzi Protection Station	2634m	KY096326	
S381	*Pseudopoda sinapophysis*	LJPZ	Yunnan, China	Linjiapuzi Protection Station	2642m	KY096327	
S382	*Pseudopoda sinapophysis*	LJPZ	Yunnan, China	Linjiapuzi Protection Station	2642m	KY096328	
S383	*Pseudopoda sinapophysis*	LJPZ	Yunnan, China	Linjiapuzi Protection Station	2642m	KY096329	
S385	*Pseudopoda sinapophysis*	LJPZ	Yunnan, China	Linjiapuzi Protection Station	2633m	KY096330	
S386	*Pseudopoda sinapophysis*	LJPZ	Yunnan, China	Linjiapuzi Protection Station	2630m	KY096331	
S387	*Pseudopoda sinapophysis*	LJPZ	Yunnan, China	Linjiapuzi Protection Station	2630m	KY096332	
S390	*Pseudopoda sinapophysis*	LJPZ	Yunnan, China	Linjiapuzi Protection Station	2633m	KY096333	
S391	*Pseudopoda sinapophysis*	LJPZ	Yunnan, China	Linjiapuzi Protection Station	2604m	KY096334	
S392	*Pseudopoda sinapophysis*	LJPZ	Yunnan, China	Linjiapuzi Protection Station	2628m	KY096335	
S393	*Pseudopoda sinapophysis*	LJPZ	Yunnan, China	Linjiapuzi Protection Station	2494m	KY096336	
S394	*Pseudopoda sinapophysis*	LJPZ	Yunnan, China	Linjiapuzi Protection Station	2459m	KY096337	
S395	*Pseudopoda sinapophysis*	LJPZ	Yunnan, China	Linjiapuzi Protection Station	2441m	KY096338	
S396	*Pseudopoda sinapophysis*	LJPZ	Yunnan, China	Linjiapuzi Protection Station	2441m	KY096339	
S397	*Pseudopoda sinapophysis*	LJPZ	Yunnan, China	Linjiapuzi Protection Station	2441m	KY096340	
S399	*Pseudopoda sinapophysis*	LJPZ	Yunnan, China	Linjiapuzi Protection Station	2408m	KY096341	
S400	*Pseudopoda sinapophysis*	LJPZ	Yunnan, China	Linjiapuzi Protection Station	2411m	KY096342	
S406	*Pseudopoda sinapophysis*	LJPZ	Yunnan, China	Linjiapuzi Protection Station	2642m	KY096343	
S407	*Pseudopoda sinapophysis*	LJPZ	Yunnan, China	Linjiapuzi Protection Station	2660m	KY096344	

S408	*Pseudopoda sinapophysis*	LJPZ	Yunnan, China	Linjiapuzi Protection Station	2617m	KY096345	
S409	*Pseudopoda sinapophysis*	LJPZ	Yunnan, China	Linjiapuzi Protection Station	2626m	KY096346	
S410	*Pseudopoda sinapophysis*	LJPZ	Yunnan, China	Linjiapuzi Protection Station	2632m	KY096347	
S411	*Pseudopoda sinapophysis*	LJPZ	Yunnan, China	Linjiapuzi Protection Station	2614m	KY096348	
S412	*Pseudopoda sinapophysis*	LJPZ	Yunnan, China	Linjiapuzi Protection Station	2633m	KY096349	
S413	*Pseudopoda sinapophysis*	LJPZ	Yunnan, China	Linjiapuzi Protection Station	2645m	KY096350	
S414	*Pseudopoda sinapophysis*	LJPZ	Yunnan, China	Linjiapuzi Protection Station	2630m	KY096351	
S415	*Pseudopoda sinapophysis*	LJPZ	Yunnan, China	Linjiapuzi Protection Station	2631m	KY096352	
S416	*Pseudopoda sinapophysis*	LJPZ	Yunnan, China	Linjiapuzi Protection Station	2598m	KY096353	
S417	*Pseudopoda sinapophysis*	LJPZ	Yunnan, China	Linjiapuzi Protection Station	2628m	KY096354	
S418	*Pseudopoda sinapophysis*	LJPZ	Yunnan, China	Linjiapuzi Protection Station	2628m	KY096355	
S419	*Pseudopoda sinapophysis*	LJPZ	Yunnan, China	Linjiapuzi Protection Station	2503m	KY096356	
S420	*Pseudopoda sinapophysis*	LJPZ	Yunnan, China	Linjiapuzi Protection Station	2494m	KY096357	KY095983
S421	*Pseudopoda sinapophysis*	LJPZ	Yunnan, China	Linjiapuzi Protection Station	2452m	KY096358	
S422	*Pseudopoda sinapophysis*	LJPZ	Yunnan, China	Linjiapuzi Protection Station	2380m	KY096359	
S423	*Pseudopoda sinapophysis*	LJPZ	Yunnan, China	Linjiapuzi Protection Station	2387m	KY096360	
S424	*Pseudopoda sinapophysis*	LJPZ	Yunnan, China	Linjiapuzi Protection Station	2387m	KY096361	
S425	*Pseudopoda sinapophysis*	LJPZ	Yunnan, China	Linjiapuzi Protection Station	2408m	KY096362	
S518	*Pseudopoda sinapophysis*	LJPZ	Yunnan, China	Linjiapuzi Protection Station	2642m	KY096363	
S520	*Pseudopoda sinapophysis*	LJPZ	Yunnan, China	Linjiapuzi Protection Station	2614m	KY096364	
S521	*Pseudopoda sinapophysis*	LJPZ	Yunnan, China	Linjiapuzi Protection Station	2604m	KY096365	KY095984
S522	*Pseudopoda sinapophysis*	LJPZ	Yunnan, China	Linjiapuzi Protection Station	2465m	KY096366	
S741	*Pseudopoda sp1*	YFS	Yunnan, China	Yunfengshan Mountain	2275m	KY096460	KY095985
S742	*Pseudopoda sp1*	YFS	Yunnan, China	Yunfengshan Mountain	2269m	KY096461	KY095986
S743	*Pseudopoda sp1*	YFS	Yunnan, China	Yunfengshan Mountain	2061m	KY096462	KY095987
S735	*Pseudopoda sp2*	ABM	Yunnan, China	Anti-British Monument in pianm	2080m	KY096367	KY095988
S736	*Pseudopoda sp2*	ABM	Yunnan, China	Anti-British Monument in pianm	2099m	KY096504	KY096033
S737	*Pseudopoda sp2*	ABM	Yunnan, China	Anti-British Monument in pianm	2104m	KY096505	KY096034
S738	*Pseudopoda sp2*	ABM	Yunnan, China	Anti-British Monument in pianm	2062m	KY096489	
S739	*Pseudopoda sp2*	ABM	Yunnan, China	Anti-British Monument in pianm	2058m	KY096490	
S10	*Pseudopoda sp3*	HS	Anhui, China	Huangshan Mountain		KY096491	KY096031
S862	*Pseudopoda sp3*	HS	Anhui, China	Huangshan Mountain		KY096492	
S863	*Pseudopoda sp3*	HS	Anhui, China	Huangshan Mountain		KY096493	
S864	*Pseudopoda sp3*	HS	Anhui, China	Huangshan Mountain		KY096494	
S865	*Pseudopoda sp3*	HS	Anhui, China	Huangshan Mountain		KY096495	
S866	*Pseudopoda sp3*	HS	Anhui, China	Huangshan Mountain		KY096496	
S867	*Pseudopoda sp3*	HS	Anhui, China	Huangshan Mountain		KY096497	
S868	*Pseudopoda sp3*	HS	Anhui, China	Huangshan Mountain		KY096498	
S857	*Pseudopoda sp4*	DYS	Fujian, China	Daiyunshan Mountain		KY096499	
S858	*Pseudopoda sp4*	DYS	Fujian, China	Daiyunshan Mountain		KY096500	
S4	*Pseudopoda sp5*	DYS	Fujian, China	Daiyunshan Mountain		KY096501	
S92	*Pseudopoda sp6*	YFS	Yunnan, China	Yunfengshan Mountain	2288m	KY096502	KY096032
S96	*Pseudopoda sp6*	YFS	Yunnan, China	Yunfengshan Mountain	2272m	KY096503	
S98	*Pseudopoda sp6*	YFS	Yunnan, China	Yunfengshan Mountain	2273m	KY096506	KY096035
S99	*Pseudopoda sp6*	YFS	Yunnan, China	Yunfengshan Mountain	2234m	KY096507	
S106	*Pseudopoda sp6*	YFS	Yunnan, China	Yunfengshan Mountain	2260m	KY096376	KY096022
S107	*Pseudopoda sp6*	YFS	Yunnan, China	Yunfengshan Mountain	2273m	KY096377	KY096023
S108	*Pseudopoda sp6*	YFS	Yunnan, China	Yunfengshan Mountain	2278m	KY096375	KY096021
S111	*Pseudopoda sp6*	YFS	Yunnan, China	Yunfengshan Mountain	2230m	KY096378	KY095994
S113	*Pseudopoda sp6*	YFS	Yunnan, China	Yunfengshan Mountain	2256m	KY096379	KY096042
S375	*Pseudopoda sp6*	LJPZ	Yunnan, China	Linjiapuzi Protection Station	2352m	KY096380	KY096043

S376	*Pseudopoda sp6*	LJPZ	Yunnan, China	Linjiapuzi Protection Station	2350m	KY096381	
S377	*Pseudopoda sp6*	LJPZ	Yunnan, China	Linjiapuzi Protection Station	2336m	KY096382	
S378	*Pseudopoda sp6*	LJPZ	Yunnan, China	Linjiapuzi Protection Station	2336m	KY096383	KY095998
S379	*Pseudopoda sp6*	LJPZ	Yunnan, China	Linjiapuzi Protection Station	2336m	KY096384	
S402	*Pseudopoda sp6*	LJPZ	Yunnan, China	Linjiapuzi Protection Station	2327m	KY096385	
S403	*Pseudopoda sp6*	LJPZ	Yunnan, China	Linjiapuzi Protection Station	2336m	KY096386	
S451	*Pseudopoda sp7*	ZXS	Yunnan, China	Zixishan Mountain	2505m	KY096387	KY095999
S601	*Pseudopoda sp7*	ZXS	Yunnan, China	Zixishan Mountain	2498m	KY096388	
S711	*Pseudopoda sp8*	YLS	Yunnan, China	Yuelingshan Mountain	2152m	KY096389	
S712	*Pseudopoda sp8*	YLS	Yunnan, China	Yuelingshan Mountain	2174m	KY096390	KY096000
S713	*Pseudopoda sp8*	YLS	Yunnan, China	Yuelingshan Mountain	2175m	KY096391	
S714	*Pseudopoda sp8*	YLS	Yunnan, China	Yuelingshan Mountain	2159m	KY096392	
S715	*Pseudopoda sp8*	YLS	Yunnan, China	Yuelingshan Mountain	2165m	KY096393	
S716	*Pseudopoda sp8*	YLS	Yunnan, China	Yuelingshan Mountain	2144m	KY096394	
S717	*Pseudopoda sp8*	YLS	Yunnan, China	Yuelingshan Mountain	2136m	KY096395	
S731	*Pseudopoda sp8*	YLS	Yunnan, China	Yuelingshan Mountain	2187m	KY096396	KY096001
S732	*Pseudopoda sp8*	YLS	Yunnan, China	Yuelingshan Mountain	2176m	KY096397	
S733	*Pseudopoda sp8*	YLS	Yunnan, China	Yuelingshan Mountain	2159m	KY096398	
S734	*Pseudopoda sp8*	YLS	Yunnan, China	Yuelingshan Mountain	2136m	KY096399	
S28	*Pseudopoda sp9*	LYS	Yunnan, China	Langyashan Mountain	2173m	KY096400	KY096002
S29	*Pseudopoda sp9*	LYS	Yunnan, China	Langyashan Mountain	2023m	KY096401	
S31	*Pseudopoda sp9*	LYS	Yunnan, China	Langyashan Mountain	2095m	KY096402	
S772	*Pseudopoda sp10*	HKBG	Yunnan, China	Haokang Botanical Garden		KY096403	
S254	*Pseudopoda sp11*	QSS	Yunnan, China	Qianshishan Mountain	2678m	KY096404	
S258	*Pseudopoda sp11*	QSS	Yunnan, China	Qianshishan Mountain	2486m	KY096405	
S309	*Pseudopoda sp11*	MLP	Yunnan, China	Maliping Village	2536m	KY096406	
S314	*Pseudopoda sp11*	MLP	Yunnan, China	Maliping Village	2537m	KY096407	
S315	*Pseudopoda sp11*	MLP	Yunnan, China	Maliping Village	2537m	KY096408	
S317	*Pseudopoda sp11*	MLP	Yunnan, China	Maliping Village	2537m	KY096409	
S320	*Pseudopoda sp11*	MLP	Yunnan, China	Maliping Village	2522m	KY096410	
S321	*Pseudopoda sp11*	MLP	Yunnan, China	Maliping Village	2522m	KY096411	
S323	*Pseudopoda sp11*	MLP	Yunnan, China	Maliping Village	2522m	KY096412	
S332	*Pseudopoda sp11*	MLP	Yunnan, China	Maliping Village	2541m	KY096413	KY096003
S333	*Pseudopoda sp11*	MLP	Yunnan, China	Maliping Village	2536m	KY096414	KY096004
S336	*Pseudopoda sp11*	MLP	Yunnan, China	Maliping Village	2537m	KY096415	KY096005
S339	*Pseudopoda sp11*	MLP	Yunnan, China	Maliping Village	2522m	KY096416	
S344	*Pseudopoda sp11*	MLP	Yunnan, China	Maliping Village	2481m	KY096417	
S388	*Pseudopoda sp11*	MLP	Yunnan, China	Maliping Village	2481m	KY096418	
S515	*Pseudopoda sp11*	MLP	Yunnan, China	Maliping Village	2537m	KY096419	
S516	*Pseudopoda sp11*	MLP	Yunnan, China	Maliping Village	2533m	KY096420	
S638	*Pseudopoda sp11*	QSS	Yunnan, China	Qianshishan Mountain	2698m	KY096421	
S641	*Pseudopoda sp11*	QSS	Yunnan, China	Qianshishan Mountain	2678m	KY096422	
S718	*Pseudopoda sp12*	YFS	Yunnan, China	Yunfengshan Mountain	2272m	KY096423	
S720	*Pseudopoda sp12*	YFS	Yunnan, China	Yunfengshan Mountain	2302m	KY096424	
S744	*Pseudopoda sp12*	LYS	Yunnan, China	Langyashan Mountain	2053m	KY096425	
S769	*Pseudopoda sp13*	PLD	Yunnan, China	Puladi Village	1400	KY096426	
S881	*Pseudopoda sp14*	KD	Yunnan, China	Kongdang Village	1762m	KY096427	
S882	*Pseudopoda sp14*	KD	Yunnan, China	Kongdang Village	1778m	KY096428	
S119	*Pseudopoda sp15*	YJP	Yunnan, China	Yaojiaping Protection Station	2581m	KY096429	
S120	*Pseudopoda sp15*	YJP	Yunnan, China	Yaojiaping Protection Station	2581m	KY096430	KY096006
S123	*Pseudopoda sp15*	YJP	Yunnan, China	Yaojiaping Protection Station	2550m	KY096431	KY096007

S124	*Pseudopoda sp15*	YJP	Yunnan, China	Yaojiaping Protection Station	2574m	KY096436	KY096011
S125	*Pseudopoda sp15*	YJP	Yunnan, China	Yaojiaping Protection Station	2574m	KY096432	
S126	*Pseudopoda sp15*	YJP	Yunnan, China	Yaojiaping Protection Station	2574m	KY096433	KY096008
S127	*Pseudopoda sp15*	YJP	Yunnan, China	Yaojiaping Protection Station	2574m	KY096434	KY096009
S130	*Pseudopoda sp15*	YJP	Yunnan, China	Yaojiaping Protection Station	2580m	KY096435	KY096010
S132	*Pseudopoda sp15*	YJP	Yunnan, China	Yaojiaping Protection Station	2578m	KY096437	KY096012
S135	*Pseudopoda sp15*	YJP	Yunnan, China	Yaojiaping Protection Station	2504m	KY096438	
S136	*Pseudopoda sp15*	YJP	Yunnan, China	Yaojiaping Protection Station	2504m	KY096439	
S137	*Pseudopoda sp15*	YJP	Yunnan, China	Yaojiaping Protection Station	2504m	KY096440	
S161	*Pseudopoda sp15*	YJP	Yunnan, China	Yaojiaping Protection Station	2538m	KY096441	
S162	*Pseudopoda sp15*	YJP	Yunnan, China	Yaojiaping Protection Station	2535m	KY096442	
S163	*Pseudopoda sp15*	YJP	Yunnan, China	Yaojiaping Protection Station	2504m	KY096443	
S183	*Pseudopoda sp15*	YJP	Yunnan, China	Yaojiaping Protection Station	2581m	KY096444	
S184	*Pseudopoda sp15*	YJP	Yunnan, China	Yaojiaping Protection Station	2586m	KY096445	
S185	*Pseudopoda sp15*	YJP	Yunnan, China	Yaojiaping Protection Station	2551m	KY096446	
S207	*Pseudopoda sp15*	YJP	Yunnan, China	Yaojiaping Protection Station	2555m	KY096447	
S274	*Pseudopoda sp15*	YJP	Yunnan, China	Yaojiaping Protection Station	2570m	KY096448	
S276	*Pseudopoda sp15*	YJP	Yunnan, China	Yaojiaping Protection Station	2525m	KY096449	
S277	*Pseudopoda sp15*	YJP	Yunnan, China	Yaojiaping Protection Station	2525m	KY096450	
S278	*Pseudopoda sp15*	YJP	Yunnan, China	Yaojiaping Protection Station	2509m	KY096451	KY096013
S279	*Pseudopoda sp15*	YJP	Yunnan, China	Yaojiaping Protection Station	2509m	KY096452	
S281	*Pseudopoda sp15*	YJP	Yunnan, China	Yaojiaping Protection Station	2509m	KY096453	
S282	*Pseudopoda sp15*	YJP	Yunnan, China	Yaojiaping Protection Station	2509m	KY096454	
S298	*Pseudopoda sp15*	YJP	Yunnan, China	Yaojiaping Protection Station	2569m	KY096455	KY096014
S301	*Pseudopoda sp15*	YJP	Yunnan, China	Yaojiaping Protection Station	2525m	KY096456	KY096015
S302	*Pseudopoda sp15*	YJP	Yunnan, China	Yaojiaping Protection Station	2525m	KY096457	KY096016
S305	*Pseudopoda sp15*	YJP	Yunnan, China	Yaojiaping Protection Station	2509m	KY096458	
S307	*Pseudopoda sp15*	YJP	Yunnan, China	Yaojiaping Protection Station	2509m	KY096459	KY096017
S354	*Pseudopoda sp15*	YJP	Yunnan, China	Yaojiaping Protection Station	2512m	KY096508	
S355	*Pseudopoda sp15*	YJP	Yunnan, China	Yaojiaping Protection Station	2509m	KY096509	KY096018
S499	*Pseudopoda sp16*	ZXS	Yunnan, China	Zixishan Mountain	2468m	KY096510	
S573	*Pseudopoda sp16*	ZXS	Yunnan, China	Zixishan Mountain	2469m	KY096511	KY096019
S574	*Pseudopoda sp16*	ZXS	Yunnan, China	Zixishan Mountain	2469m	KY096512	KY096020
S575	*Pseudopoda sp16*	ZXS	Yunnan, China	Zixishan Mountain	2469m	KY096513	
S577	*Pseudopoda sp16*	ZXS	Yunnan, China	Zixishan Mountain	2479m	KY096514	
S584	*Pseudopoda sp16*	ZXS	Yunnan, China	Zixishan Mountain	2480m	KY096515	KY096048
S588	*Pseudopoda sp16*	ZXS	Yunnan, China	Zixishan Mountain	2475m	KY096516	
S589	*Pseudopoda sp16*	ZXS	Yunnan, China	Zixishan Mountain	2475m	KY096517	
S591	*Pseudopoda sp16*	ZXS	Yunnan, China	Zixishan Mountain	2481m	KY096518	
S599	*Pseudopoda sp16*	ZXS	Yunnan, China	Zixishan Mountain	2465m	KY096519	
S609	*Pseudopoda sp16*	ZXS	Yunnan, China	Zixishan Mountain	2507m	KY096520	KY096049
S610	*Pseudopoda sp16*	ZXS	Yunnan, China	Zixishan Mountain	2475m	KY096521	KY096050
S611	*Pseudopoda sp16*	ZXS	Yunnan, China	Zixishan Mountain	2510m	KY096522	KY096051
S613	*Pseudopoda sp16*	ZXS	Yunnan, China	Zixishan Mountain	2486m	KY096523	KY096052
S614	*Pseudopoda sp16*	ZXS	Yunnan, China	Zixishan Mountain	2463m	KY096531	KY096056
S615	*Pseudopoda sp16*	ZXS	Yunnan, China	Zixishan Mountain	2464m	KY096532	
S616	*Pseudopoda sp16*	ZXS	Yunnan, China	Zixishan Mountain	2468m	KY096533	
S723	*Pseudopoda sp17*	LYST	Yunnan, China	Lingyuanguanyinsi Temple		KY096534	
S12	*Pseudopoda sp18*	HLT	Yunnan, China	Heilongtan Park		KY096535	KY096057
S32	*Pseudopoda sp18*	FLS	Yunnan, China	Feilaisi Temple	3452m	KY096524	KY096053
S35	*Pseudopoda sp18*	FLS	Yunnan, China	Feilaisi Temple	3443m	KY096525	

S36	*Pseudopoda sp18*	FLS	Yunnan, China	Feilaisi Temple	3458m	KY096526	
S47	*Pseudopoda sp18*	FLS	Yunnan, China	Feilaisi Temple	3445m	KY096527	
S67	*Pseudopoda sp18*	HLT	Yunnan, China	Heilongtan Park	2670m	KY096528	KY096054
S70	*Pseudopoda sp18*	HLT	Yunnan, China	Heilongtan Park	2658m	KY096529	
S71	*Pseudopoda sp18*	HLT	Yunnan, China	Heilongtan Park	2659m	KY096530	KY096055
S73	*Pseudopoda sp18*	HLT	Yunnan, China	Heilongtan Park	2658m	KY096536	KY096058
S74	*Pseudopoda sp18*	HLT	Yunnan, China	Heilongtan Park	2658m	KY096537	
S75	*Pseudopoda sp18*	HLT	Yunnan, China	Heilongtan Park	2658m	KY096538	
S76	*Pseudopoda sp18*	HLT	Yunnan, China	Heilongtan Park	2658m	KY096539	
S77	*Pseudopoda sp18*	HLT	Yunnan, China	Heilongtan Park	2650m	KY096540	KY096059
S78	*Pseudopoda sp18*	HLT	Yunnan, China	Heilongtan Park	2651m	KY096541	KY096060
S79	*Pseudopoda sp18*	HLT	Yunnan, China	Heilongtan Park	2644m	KY096542	
S82	*Pseudopoda sp18*	HLT	Yunnan, China	Heilongtan Park	2670m	KY096543	KY096061
S83	*Pseudopoda sp18*	HLT	Yunnan, China	Heilongtan Park	2658m	KY096544	
S84	*Pseudopoda sp18*	HLT	Yunnan, China	Heilongtan Park	2678m	KY096545	
S85	*Pseudopoda sp18*	HLT	Yunnan, China	Heilongtan Park	2667m	KY096546	
S86	*Pseudopoda sp18*	HLT	Yunnan, China	Heilongtan Park	2650m	KY096547	
S87	*Pseudopoda sp18*	HLT	Yunnan, China	Heilongtan Park	2630m	KY096548	
S88	*Pseudopoda sp18*	HLT	Yunnan, China	Heilongtan Park	2442m	KY096549	
S89	*Pseudopoda sp18*	HLT	Yunnan, China	Heilongtan Park	2442m	KY096550	KY096062
S22	*Pseudopoda sp19*	LYS	Yunnan, China	Langyashan Mountain	2061m	KY096551	
S24	*Pseudopoda sp19*	LYS	Yunnan, China	Langyashan Mountain	2021m	KY096552	
S719	*Pseudopoda sp19*	YFS	Yunnan, China	Yunfengshan Mountain	2250m	KY096482	KY096024
S721	*Pseudopoda sp19*	YFS	Yunnan, China	Yunfengshan Mountain	2263m	KY096483	KY096025
S722	*Pseudopoda sp19*	YFS	Yunnan, China	Yunfengshan Mountain	1985m	KY096484	KY096026
S430	*Pseudopoda spiculata*	XS	Yunnan, China	Xishan Mountain	2185m	KY096554	
S432	*Pseudopoda spiculata*	XS	Yunnan, China	Xishan Mountain	2112m	KY096555	KY096064
S443	*Pseudopoda spiculata*	XS	Yunnan, China	Xishan Mountain	2219m	KY096556	
S447	*Pseudopoda spiculata*	XS	Yunnan, China	Xishan Mountain	2244m	KY096557	
S448	*Pseudopoda spiculata*	XS	Yunnan, China	Xishan Mountain	2210m	KY096558	
S449	*Pseudopoda spiculata*	XS	Yunnan, China	Xishan Mountain	2227m	KY096559	
S450	*Pseudopoda spiculata*	XS	Yunnan, China	Xishan Mountain	2270m	KY096560	
S463	*Pseudopoda spiculata*	XS	Yunnan, China	Xishan Mountain	2219m	KY096561	
S465	*Pseudopoda spiculata*	XS	Yunnan, China	Xishan Mountain	2144m	KY096562	
S466	*Pseudopoda spiculata*	XS	Yunnan, China	Xishan Mountain	2144m	KY096563	
S472	*Pseudopoda spiculata*	XS	Yunnan, China	Xishan Mountain	2168m	KY096564	
S474	*Pseudopoda spiculata*	XS	Yunnan, China	Xishan Mountain	2225m	KY096565	
S483	*Pseudopoda spiculata*	XS	Yunnan, China	Xishan Mountain	2008m	KY096566	
S486	*Pseudopoda spiculata*	XS	Yunnan, China	Xishan Mountain	2049m	KY096567	
S488	*Pseudopoda spiculata*	XS	Yunnan, China	Xishan Mountain	2144m	KY096568	
S489	*Pseudopoda spiculata*	XS	Yunnan, China	Xishan Mountain	2151m	KY096569	KY096065
S491	*Pseudopoda spiculata*	XS	Yunnan, China	Xishan Mountain	2114m	KY096570	
S493	*Pseudopoda spiculata*	XS	Yunnan, China	Xishan Mountain	2220m	KY096571	
S494	*Pseudopoda spiculata*	XS	Yunnan, China	Xishan Mountain	2200m	KY096572	
S500	*Pseudopoda spiculata*	XS	Yunnan, China	Xishan Mountain	2121m	KY096573	
S502	*Pseudopoda spiculata*	XS	Yunnan, China	Xishan Mountain	2057m	KY096574	
S509	*Pseudopoda spiculata*	XS	Yunnan, China	Xishan Mountain	2217m	KY096575	
S510	*Pseudopoda spiculata*	XS	Yunnan, China	Xishan Mountain	2144m	KY096576	KY096066
S15	*Pseudopoda tiantangensis*	TTZ	Hubei, China	Tiantangzhai National Forest Park		KY096577	KY096067
S832	*Pseudopoda tiantangensis*	TTZ	Hubei, China	Tiantangzhai National Forest Park		KY096578	KY096068
S833	*Pseudopoda tiantangensis*	TTZ	Hubei, China	Tiantangzhai National Forest Park		KY096579	

S834	*Pseudopoda tiantangensis*	TTZ	Hubei, China	Tiantangzhai National Forest Park		KY096580	
S835	*Pseudopoda tiantangensis*	TTZ	Hubei, China	Tiantangzhai National Forest Park		KY096581	
S836	*Pseudopoda tiantangensis*	TTZ	Hubei, China	Tiantangzhai National Forest Park		KY096582	KY096069
S140	*Pseudopoda yunnanensis ev*	EWS	Yunnan, China	Erwushan Mountain	2384m	KY096583	
S147	*Pseudopoda yunnanensis ev*	EWS	Yunnan, China	Erwushan Mountain	2377m	KY096584	
S767	*Pseudopoda yunnanensis ev*	EWS	Yunnan, China	Erwushan Mountain	2377m	KY096585	KY096044
S768	*Pseudopoda yunnanensis ev*	EWS	Yunnan, China	Erwushan Mountain	2376m	KY096586	KY096045
S215	*Pseudopoda yunnanensis qs*	QSS	Yunnan, China	Qianshishan Mountain	2682m	KY096587	
S218	*Pseudopoda yunnanensis qs*	QSS	Yunnan, China	Qianshishan Mountain	2740m	KY096588	
S219	*Pseudopoda yunnanensis qs*	QSS	Yunnan, China	Qianshishan Mountain	2730m	KY096589	
S220	*Pseudopoda yunnanensis qs*	QSS	Yunnan, China	Qianshishan Mountain	2726m	KY096590	KY096046
S221	*Pseudopoda yunnanensis qs*	QSS	Yunnan, China	Qianshishan Mountain	2729m	KY096591	
S223	*Pseudopoda yunnanensis qs*	QSS	Yunnan, China	Qianshishan Mountain	2647m	KY096592	
S227	*Pseudopoda yunnanensis qs*	QSS	Yunnan, China	Qianshishan Mountain	2678m	KY096593	
S229	*Pseudopoda yunnanensis qs*	QSS	Yunnan, China	Qianshishan Mountain	2678m	KY096594	
S639	*Pseudopoda yunnanensis qs*	QSS	Yunnan, China	Qianshishan Mountain	2682m	KY096595	KY096047
S640	*Pseudopoda yunnanensis qs*	QSS	Yunnan, China	Qianshishan Mountain	2693m	KY096596	
S644	*Pseudopoda yunnanensis wj*	WFS	Yunnan, China	Wufengshan Mountain	3654m	KY096597	KY096070
S645	*Pseudopoda yunnanensis wj*	WFS	Yunnan, China	Wufengshan Mountain	3654m	KY096598	
S646	*Pseudopoda yunnanensis wj*	WFS	Yunnan, China	Wufengshan Mountain	3662m	KY096599	
S647	*Pseudopoda yunnanensis wj*	WFS	Yunnan, China	Wufengshan Mountain	3662m	KY096600	
S648	*Pseudopoda yunnanensis wj*	WFS	Yunnan, China	Wufengshan Mountain	3662m	KY096601	
S649	*Pseudopoda yunnanensis wj*	WFS	Yunnan, China	Wufengshan Mountain	3651m	KY096602	
S650	*Pseudopoda yunnanensis wj*	WFS	Yunnan, China	Wufengshan Mountain	3627m	KY096603	
S651	*Pseudopoda yunnanensis wj*	WFS	Yunnan, China	Wufengshan Mountain	3619m	KY096604	
S652	*Pseudopoda yunnanensis wj*	WFS	Yunnan, China	Wufengshan Mountain	3600m	KY096605	
S653	*Pseudopoda yunnanensis wj*	WFS	Yunnan, China	Wufengshan Mountain	3615m	KY096606	
S654	*Pseudopoda yunnanensis wj*	WFS	Yunnan, China	Wufengshan Mountain	3615m	KY096607	
S655	*Pseudopoda yunnanensis wj*	WFS	Yunnan, China	Wufengshan Mountain	3599m	KY096608	
S656	*Pseudopoda yunnanensis wj*	WFS	Yunnan, China	Wufengshan Mountain	3596m	KY096609	
S657	*Pseudopoda yunnanensis wj*	WFS	Yunnan, China	Wufengshan Mountain	3596m	KY096610	
S658	*Pseudopoda yunnanensis wj*	WFS	Yunnan, China	Wufengshan Mountain	3569m	KY096611	
S659	*Pseudopoda yunnanensis wj*	WFS	Yunnan, China	Wufengshan Mountain	3560m	KY096612	
S660	*Pseudopoda yunnanensis wj*	WFS	Yunnan, China	Wufengshan Mountain	3563m	KY096613	
S661	*Pseudopoda yunnanensis wj*	WFS	Yunnan, China	Wufengshan Mountain	3558m	KY096614	KY096071
S668	*Pseudopoda yunnanensis wj*	WFS	Yunnan, China	Wufengshan Mountain	3656m	KY096615	
S669	*Pseudopoda yunnanensis wj*	WFS	Yunnan, China	Wufengshan Mountain	3656m	KY096616	
S670	*Pseudopoda yunnanensis wj*	WFS	Yunnan, China	Wufengshan Mountain	3654m	KY096617	
S671	*Pseudopoda yunnanensis wj*	WFS	Yunnan, China	Wufengshan Mountain	3654m	KY096618	
S672	*Pseudopoda yunnanensis wj*	WFS	Yunnan, China	Wufengshan Mountain	3654m	KY096619	
S673	*Pseudopoda yunnanensis wj*	WFS	Yunnan, China	Wufengshan Mountain	3662m	KY096620	
S674	*Pseudopoda yunnanensis wj*	WFS	Yunnan, China	Wufengshan Mountain	3662m	KY096621	
S675	*Pseudopoda yunnanensis wj*	WFS	Yunnan, China	Wufengshan Mountain	3662m	KY096622	
S676	*Pseudopoda yunnanensis wj*	WFS	Yunnan, China	Wufengshan Mountain	3662m	KY096623	
S677	*Pseudopoda yunnanensis wj*	WFS	Yunnan, China	Wufengshan Mountain	3631m	KY096624	
S678	*Pseudopoda yunnanensis wj*	WFS	Yunnan, China	Wufengshan Mountain	3629m	KY096625	
S679	*Pseudopoda yunnanensis wj*	WFS	Yunnan, China	Wufengshan Mountain	3602m	KY096626	
S680	*Pseudopoda yunnanensis wj*	WFS	Yunnan, China	Wufengshan Mountain	3602m	KY096627	KY096072
S681	*Pseudopoda yunnanensis wj*	WFS	Yunnan, China	Wufengshan Mountain	3599m	KY096628	
S682	*Pseudopoda yunnanensis wj*	WFS	Yunnan, China	Wufengshan Mountain	3585m	KY096629	
S683	*Pseudopoda yunnanensis wj*	WFS	Yunnan, China	Wufengshan Mountain	3583m	KY096630	

S684	*Pseudopoda yunnanensis wj*	WFS	Yunnan, China	Wufengshan Mountain	3584m	KY096631	
S685	*Pseudopoda yunnanensis wj*	WFS	Yunnan, China	Wufengshan Mountain	3580m	KY096632	
S686	*Pseudopoda yunnanensis wj*	WFS	Yunnan, China	Wufengshan Mountain	3578m	KY096633	
S687	*Pseudopoda yunnanensis wj*	WFS	Yunnan, China	Wufengshan Mountain	3515m	KY096634	
S688	*Pseudopoda yunnanensis wj*	WFS	Yunnan, China	Wufengshan Mountain	3521m	KY096635	
S689	*Pseudopoda yunnanensis wj*	WFS	Yunnan, China	Wufengshan Mountain	3524m	KY096636	
S690	*Pseudopoda yunnanensis wj*	WFS	Yunnan, China	Wufengshan Mountain	3528m	KY096637	
S691	*Pseudopoda yunnanensis wj*	WFS	Yunnan, China	Wufengshan Mountain	3482m	KY096638	
S694	*Pseudopoda yunnanensis wj*	WFS	Yunnan, China	Wufengshan Mountain	3662m	KY096639	KY096073
S695	*Pseudopoda yunnanensis wj*	WFS	Yunnan, China	Wufengshan Mountain	3662m	KY096640	
S696	*Pseudopoda yunnanensis wj*	WFS	Yunnan, China	Wufengshan Mountain	3602m	KY096641	
S697	*Pseudopoda yunnanensis wj*	WFS	Yunnan, China	Wufengshan Mountain	3599m	KY096642	
S698	*Pseudopoda yunnanensis wj*	WFS	Yunnan, China	Wufengshan Mountain	3534m	KY096643	
S261	*Sinopoda anguina*	YJP	Yunnan, China	Yaojiaping Protection Station	2588m	KY096645	
S457	*Sinopoda pengi*	XS	Yunnan, China	Xishan Mountain	2025m	KY096644	
gb	*Pseudopoda confusa*	BTM	Luang Nam Tha, Laos	Ban Tavan Mai		KJ408771	
gb	*Pseudopoda prompta*	JSM	Uttarakhand, India	Joshimath		KJ408772	
gb	*Pseudopoda namkhan*		Luang Prabang, Laos	Ban En Savan, ThatSe		GQ855821	

Table S2. Descriptive statistics for intraspecific and interspecific K2P (Kimura 2-parameter) distances, number of individuals and comparisons for each focal *Pseudopoda* species based on COI, ITS2 and COI+ITS2 datasets.

		COI		ITS2		COI+ITS2	
		Intraspec. stat.	Interspec. stat.	Intraspec. stat.	Interspec. stat.	Intraspec. stat.	Interspec. stat.
P. bibulba	Mean	0.046486364	0.166375887	.00167	.04944	.03128	.12034
	SE	0.000946379	0.000172402	.000252	.000757	.003233	.000820
	Median	0.047	0.161	.00300	.04200	.04500	.12000
	SD	0.037138596	0.037285076	.001512	.025977	.019399	.028161
	Minimum	0	0.076	0.000	.017	0.000	.068
	Maximum	0.087	0.277	.003	.172	.049	.201
	No.ind/No.com	56/1540	56/46772	36	1179	36	1179
P. bicruris	Mean	0.006333333	0.222945518	0.005333333	0.073790754	0.006666667	0.152863747
	SE	0.001358103	0.000666027	0.001452966	0.001038994	0.000333333	0.001331309
	Median	0.007	0.218	0.005	0.074	0.007	0.152
	SD	0.00332666	0.031774447	0.002516611	0.021063667	0.00057735	0.026989807
	Minimum	0	0.122	0.003	0.011	0.006	0.071
	Maximum	0.01	0.315	0.008	0.126	0.007	0.213
	No.ind/No.com	4/6	4/2276	3	411	3	411
P. cangschana	Mean	0.001185374	0.19434468	0	0.055854015	0.001333333	0.136238443
	SE	6.15193E-05	0.000215967	0	0.001466637	0.000666667	0.001522109
	Median	0	0.194	0	0.046	0.002	0.133
	SD	0.002109671	0.034605951	0	0.029733337	0.001154701	0.030857921
	Minimum	0	0.086	0	0.008	0	0.056
	Maximum	0.011	0.345	0	0.194	0.002	0.219
	No.ind/No.com	49/1176	49/25676	3	411	3	411
P. confusa	Mean	0.037	0.198274081		0.047028777		0.140244604
	SE		0.000697661		0.001269902		0.001689599
	Median	0.037	0.198		0.049		0.138
	SD		0.023576404		0.014971927		0.019920073
	Minimum	0.037	0.149		0.02		0.107
	Maximum	0.037	0.296		0.093		0.194
	No.ind/No.com	2/1	2/1142		139		139
P. daliensis	Mean	0	0.171136504	0.003333333	0.044924574	0.001333333	0.118323601
	SE	0	0.000453512	0.001666667	0.001254473	0.000666667	0.001243916
	Median	0	0.172	0.005	0.039	0.002	0.116
	SD	0	0.030490011	0.002886751	0.025432103	0.001154701	0.025218071
	Minimum	0	0.079	0	0.005	0	0.05
	Maximum	0	0.254	0.005	0.165	0.002	0.183
	No.ind/No.com	8/28	8/4520	3	411	3	411
P. digitata	Mean	0	0.181134886	0	0.048007353	0	0.125830882
	SE	0	0.000640871	0	0.00109722	0	0.000996936
	Median	0	0.177	0	0.042	0	0.122

	SD	0	0.030574324	0	0.025591348	0	0.023252337
	Minimum	0	0.101	0	0.014	0	0.068
	Maximum	0	0.255	0	0.165	0	0.192
	No.ind/No.com	4/6	4/2276	6	544	6	544
P. gibberosa	Mean	0.002967742	0.177063326	0	0.050141119	0.004666667	0.122841849
	SE	0.000208177	0.000209834	0	0.001195574	0.002333333	0.001179063
	Median	0.002	0.172	0	0.045	0.007	0.119
	SD	0.004489095	0.027199187	0	0.024238038	0.004041452	0.023903297
	Minimum	0	0.112	0	0.014	0	0.068
	Maximum	0.014	0.259	0	0.155	0.007	0.2
	No.ind/No.com	31/465	31/16802	3	411	3	411
P. interposita	Mean	0.001715789	0.156145841	0.0015	0.058012868	.00250	0.121612132
	SE	0.000175072	0.000412076	0.00067082	0.00110702	.000671	0.001153262
	Median	0	0.164	0.0015	0.052	.00250	0.119
	SD	0.002413205	0.043336617	0.001643168	0.025819921	.001643	0.026898457
	Minimum	0	0.061	0	0.023	.000000	0.05
	Maximum	0.008	0.256	0.003	0.175	.005000	0.197
	No.ind/No.com	20/190	20/11060	6	544	6	544
P. kunmingensis	Mean	0.001777778	0.178339787	0	0.043399027		0.120374696
	SE	0.00036082	0.000381125	0	0.00123827		0.0012065
	Median	0	0.177	0	0.036		0.12
	SD	0.002420452	0.028597056	0	0.025103617		0.024459538
	Minimum	0	0.08	0	0.005		0.05
	Maximum	0.005	0.259	0	0.158		0.198
	No.ind/No.com	10/45	10/5630	3	411		411
P. lushanensis	Mean	0.005133333	0.200594944	0.003666667	0.061430657	0.005333333	0.138717762
	SE	0.000827312	0.000437398	0.000666667	0.00104943	0.000333333	0.001096916
	Median	0.007	0.203	0.003	0.059	0.005	0.14
	SD	0.003204164	0.025511964	0.001154701	0.021275245	0.00057735	0.022237919
	Minimum	0	0.116	0.003	0.029	0.005	0.089
	Maximum	0.007	0.274	0.005	0.159	0.006	0.197
	No.ind/No.com	6/15	6/3402	3	411	3	411
P. mediana	Mean	0.003	0.21599331	0.002	0.091121655	0.002666667	0.162248175
	SE	0.000632456	0.000538101	0.001	0.000999679	0.000666667	0.000892151
	Median	0.003	0.214	0.003	0.09	0.002	0.161
	SD	0.002	0.028676285	0.001732051	0.020266632	0.001154701	0.018086694
	Minimum	0	0.153	0	0.059	0.002	0.125
	Maximum	0.005	0.309	0.003	0.182	0.004	0.215
	No.ind/No.com	5/10	5/2840	3	411	3	411
P. namkhan	Mean	0.048377778	0.232415885	0	.07057	0.022714286	0.158497315
	SE	0.002137281	0.000511878	0	.000587	0.002754341	0.000826523
	Median	0.052	0.231	0	.07000	0.03	0.158
	SD	0.014337314	0.040246739	0	.017907	0.012621976	0.025219099
	Minimum	0	0.061	0	.011	0	0.071

	Maximum	0.066	0.347	0	.136	0.033	0.232
	No.ind/No.com	11/45	11/6182	21	931	21	931
P. prompta	Mean		0.172898601				
	SE		0.001133208				
	Median		0.167				
	SD		0.027102391				
	Minimum		0.117				
	Maximum		0.237				
	No.ind/No.com	1	1/572				
P. recta	Mean	0.026428571	0.223693337	0.0033	0.132328889	0.0177	0.183780741
	SE	0.003516337	0.000346992	0.000746101	0.000773903	0.003130673	0.000791866
	Median	0.034	0.224	0.005	0.136	0.0225	0.184
	SD	0.01611388	0.021841193	0.002359378	0.020106578	0.009900056	0.020573295
	Minimum	0	0.093	0	0.05	0.004	0.076
	Maximum	0.045	0.303	0.005	0.183	0.029	0.245
	No.ind/No.com	7/21	7/3962	10	675	10	675
P. rivicola	Mean	0.000924242	0.182065954	0	0.054532847	0.000666667	0.132128954
	SE	0.000131887	0.000561425	0	0.001396989	0.000333333	0.001594807
	Median	0	0.184	0	0.045	0.001	0.132
	SD	0.001071456	0.046064256	0	0.02832134	0.00057735	0.032331747
	Minimum	0	0.086	0	0.008	0	0.056
	Maximum	0.003	0.307	0	0.186	0.001	0.2
	No.ind/No.com	12/66	12/6732	3	411	3	411
P. roganda	Mean	0.000397661	0.22284277	0	0.05389781	0.000666667	0.149077859
	SE	6.12222E-05	0.000269867	0	0.001245555	0.000333333	0.001224339
	Median	0	0.223	0	0.045	0.001	0.146
	SD	0.000800585	0.027687374	0	0.025251308	0.00057735	0.02482119
	Minimum	0	0.152	0	0.023	0	0.104
	Maximum	0.002	0.32	0	0.174	0.001	0.228
	No.ind/No.com	19/171	19/10526	3	411	3	411
P. semiannulata	Mean	0.004666667	0.243382456	0.001	0.083065693	0.003	0.175043796
	SE	0.001452966	0.000894917	0.001	0.001383875	0.001154701	0.001278749
	Median	0.047	0.228	0	0.075	0.003	0.171
	SD	0.002516611	0.037006743	0.001732051	0.028055486	0.002	0.025924243
	Minimum	0.002	0.174	0	0.039	0.001	0.124
	Maximum	0.007	0.347	0.003	0.24	0.005	0.245
	No.ind/No.com	3/3	3/1710	3/3	411	3	411
P. serrata	Mean		0.206910839		0.160007194		0.185824818
	SE		0.000991292		0.002511168		0.002310646
	Median		0.201		0.165		0.189
	SD		0.023708255		0.029606231		0.027045413
	Minimum		0.093		0.05		0.076
	Maximum		0.269		0.24		0.233
	No.ind/No.com	1	1/572		139		139

	No.ind/No.com	19/171	19/10526	15	804	15	804
P. sinapophysis	Mean	0.000594389	0.165949977	0	0.065437956	0	0.127090024
	SE	2.56869E-05	0.000235457	0	0.00121311	0	0.001415781
	Median	0	0.167	0	0.063	0	0.126
	SD	0.001062521	0.041003255	0	0.024593533	0	0.028702327
	Minimum	0	0.066	0	0.023	0	0.05
	Maximum	0.005	0.248	0	0.169	0	0.202
	No.ind/No.com	59/1711	59/30326	3	411	3	411
P. sp1	Mean	0	0.195980117	0.002	0.047085158	0.000666667	0.130041363
	SE	0	0.000623894	0.001	0.001139199	0.000333333	0.001017438
	Median	0	0.193	0.003	0.042	0.001	0.125
	SD	0	0.025799349	0.001732051	0.023095138	0.00057735	0.020626664
	Minimum	0	0.143	0	0.011	0	0.099
	Maximum	0	0.296	0.003	0.158	0.001	0.199
	No.ind/No.com	3/3	3/1710	3	411	3	411
P. sp2	Mean	0	0.198655282	0	0.055562044	0	0.135002433
	SE	0	0.00042287	0	0.000922815	0	0.000913833
	Median	0	0.198	0	0.052	0	0.134
	SD	0	0.02253546	0	0.018708363	0	0.01852625
	Minimum	0	0.131	0	0.029	0	0.086
	Maximum	0	0.278	0	0.152	0	0.193
	No.ind/No.com	5/10	5/2840	3	411	3	411
P. sp3	Mean	0.003107143	0.192642257	0.003	0.055413043	0.003	0.130826087
	SE	0.000389476	0.000337809		0.001356237		0.001241528
	Median	0.0025	0.193	0.003	0.052	0.003	0.131
	SD	0.002060911	0.022711223		0.022531506		0.020625814
	Minimum	0	0.122	0.003	0.023	0.003	0.079
	Maximum	0.008	0.289	0.003	0.156	0.003	0.192
	No.ind/No.com	8/28	8/4520	1	276	1	276
P. sp4	Mean	0	0.178370403	0	0.045202899	0	0.118268116
	SE		0.000623784		0.001170761		0.001086655
	Median	0	0.18	0	0.042	0	0.118
	SD	0	0.021079839		0.019450136		0.018052868
	Minimum	0	0.119	0	0.02	0	0.075
	Maximum	0	0.246	0	0.138	0	0.171
	No.ind/No.com	2/1	2/1142	1	276	1	276
P. sp5	Mean		0.187744755		0.047223022		0.129057554
	SE		0.001111585		0.001660034		0.002148287
	Median		0.187		0.042		0.126
	SD		0.026585247		0.019571513		0.025327927
	Minimum		0.12		0.023		0.078
	Maximum		0.283		0.128		0.209
	No.ind/No.com	1	1/572		139		139
P. sp6	Mean	0.012675	0.233414385	0	0.058231343	0.0078	0.152378109

	SE	0.00103145	0.000332485	0	0.000974813	0.0017021	0.00084661
	Median	0.021	0.233	0	0.053	0.013	0.15
	SD	0.011298964	0.031387673	0	0.027640707	0.006592203	0.024005527
	Minimum	0	0.139	0	0.017	0	0.083
	Maximum	0.028	0.349	0	0.195	0.013	0.224
	No.ind/No.com	16/120	16/8912	15	804	15	804
P. sp7	Mean	0	0.160607706	0	0.04623913	0	0.1125
	SE		0.000867806		0.001426856		0.001453182
	Median	0	0.166	0	0.042	0	0.114
	SD	0	0.029326179		0.023704715		0.024142079
	Minimum	0	0.067	0	0.003	0	0.041
	Maximum	0	0.235	0	0.165	0	0.193
	No.ind/No.com	2/1	2/1142	1	276	1	276
P. sp8	Mean	0.008436364	0.161998706	0	0.038644928	0.009	.10895
	SE	0.000979852	0.000330396		0.001474809		.001421
	Median	0.015	0.164	0	0.032	0.009	.10600
	SD	0.007266778	0.025977577		0.024501369		.023615
	Minimum	0	0.079	0	0.008	0.009	.050
	Maximum	0.015	0.235	0	0.149	0.009	.174
	No.ind/No.com	11/55	11/6182	1	276	1	276
P. sp9	Mean	0.009666667	0.22614386	0.002	0.053323601	0.006666667	0.14689781
	SE	0.003333333	0.000861832	0.001	0.001251232	0.002333333	0.001280447
	Median	0.013	0.226	0.003	0.048	0.009	0.145
	SD	0.005773503	0.035638591	0.001732051	0.025366386	0.004041452	0.025958678
	Minimum	0.003	0.139	0	0.02	0.002	0.083
	Maximum	0.013	0.32	0.003	0.187	0.009	0.221
	No.ind/No.com	3/3	3/1710	3	411	3	411
P. sp10	Mean		0.221318182		0.078129496		0.157928058
	SE		0.001278872		0.001927224		0.001993516
	Median		0.214		0.075		0.158
	SD		0.030586162		0.022721641		0.023503204
	Minimum		0.14		0.029		0.096
	Maximum		0.306		0.148		0.21
	No.ind/No.com	1	1/572		139		139
P. sp11	Mean	0.001666667	0.200667775	0	0.063303704	0.0004	0.147611852
	SE	0.000197508	0.000422509	0	0.00108927	0.000163299	0.001227127
	Median	0	0.205	0	0.055	0	0.144
	SD	0.002582748	0.043347842	0	0.028300076	0.000516398	0.031881697
	Minimum	0	0.094	0	0.035	0	0.072
	Maximum	0.012	0.343	0	0.175	0.001	0.226
	No.ind/No.com	19/171	19/10526	10	675	10	675
P. sp12	Mean	0.01	0.176752632	0	0.062978102	0.006	0.129306569
	SE	0.003605551	0.000730384	0	0.000898052	0.002081666	0.0009635
	Median	0.012	0.173	0	0.062	0.007	0.126

	SD	0.006244998	0.030202926	0	0.018206324	0.003605551	0.019533172
	Minimum	0.003	0.118	0	0.029	0.002	0.086
	Maximum	0.015	0.278	0	0.137	0.009	0.185
	No.ind/No.com	3/3	3/1710	3	411	3	411
P. sp13	Mean		0.237506993		0.063834532		0.158223022
	SE		0.001950016		0.001338871		0.001985902
	Median		0.239		0.063		0.158
	SD		0.046637608		0.015785059		0.023413437
	Minimum		0.068		0.025		0.087
	Maximum		0.349		0.133		0.201
	No.ind/No.com	1	1/572		139		139
P. sp14	Mean	0	0.220302102	0	0.087210145	0	0.161083333
	SE		0.000725045		0.001352896		0.001026545
	Median	0	0.22	0	0.0825	0	0.1595
	SD		0.024501816		0.022475992		0.017054254
	Minimum	0	0.168	0	0.049	0	0.129
	Maximum	0	0.284	0	0.187	0	0.202
	No.ind/No.com	2/1	2/1142	1	276	1	276
P. sp15	Mean	0.002176136	0.167839113	0.015266667	0.071416667	0.008866667	0.131493781
	SE	0.000112675	0.000313955	0.00429004	0.000816454	0.001812304	0.0008688
	Median	0.002	0.171	0.011	0.067	0.009	0.133
	SD	0.002589066	0.041910289	0.016615254	0.023150475	0.007019022	0.024634741
	Minimum	0	0.061	0	0.026	0	0.051
	Maximum	0.008	0.245	0.039	0.183	0.019	0.206
	No.ind/No.com	33/528	33/17820	15	804	15	804
P. sp16	Mean	0.001845588	0.200012802	0	0.05210219	0.001333333	0.139484185
	SE	0.000118487	0.000334835	0	0.001217978	0.000333333	0.00111025
	Median	0.002	0.1975	0	0.045	0.001	0.132
	SD	0.001381779	0.032553152	0	0.024692238	0.00057735	0.022508245
	Minimum	0	0.142	0	0.023	0.001	0.105
	Maximum	0.005	0.301	0	0.17	0.002	0.199
	No.ind/No.com	17/136	17/9452	3	411	3	411
P. sp17	Mean		0.18327972		0.053352518		0.130071942
	SE		0.001983034		0.002397864		0.002921837
	Median		0.178		0.052		0.128
	SD		0.047427283		0.028270398		0.034447953
	Minimum		0.1		0.017		0.064
	Maximum		0.303		0.16		0.194
	No.ind/No.com	1	1/572		139		139
P. sp18	Mean	0.018636364	0.161451542	0.004	0.06480597	0.0222	0.129636816
	SE	0.001317812	0.000373649	0.000755929	0.000902542	0.003996665	0.001252329
	Median	0.007	0.164	0.005	0.064	0.034	0.1265
	SD	0.020961089	0.04202516	0.0029277	0.025591477	0.015479018	0.035509667
	Minimum	0	0.056	0	0.029	0	0.053

	Maximum	0.058	0.317	0.008	0.161	0.035	0.209
	No.ind/No.com	23/253	23/12650	15	804	15	804
P. sp19	Mean	0.0065	0.187274296	0.006666667	0.056477941	0.007	0.131277574
	SE	0.001648231	0.000559527	0.001725624	0.000906294	0.00167332	0.000737728
	Median	0.01	0.181	0.0065	0.052	0.009	0.13
	SD	0.005212165	0.029818101	0.004226898	0.021138225	0.00409878	0.017206635
	Minimum	0	0.136	0	0.02	0	0.093
	Maximum	0.011	0.283	0.011	0.169	0.01	0.184
	No.ind/No.com	5/10	5/2840	6	544	6	544
P. spiculata	Mean	0.001521739	0.160314229	0	0.047416058	0.000666667	0.115175309
	SE	7.22847E-05	0.000199032	0	0.001187266	0.000333333	0.001075435
	Median	0.002	0.158	0	0.042	0.001	0.113
	SD	0.001149759	0.022385584	0	0.024069607	0.00057735	0.02164272
	Minimum	0	0.067	0	0.003	0	0.072
	Maximum	0.004	0.239	0	0.17	0.001	0.194
	No.ind/No.com	23/253	23/12650	3	411	3	411
P. tiantangensis	Mean	0.009333333	0.171519694	0.002	0.046481752	0.007	0.118298765
	SE	0.002039296	0.000418855	0.001	0.001061239	0.00305505	0.001023322
	Median	0.015	0.167	0.003	0.042	0.009	0.113
	SD	0.007898161	0.024430407	0.001732051	0.021514636	0.005291503	0.020593956
	Minimum	0	0.116	0	0.017	0	0.081
	Maximum	0.016	0.255	0.003	0.147	0.011	0.178
	No.ind/No.com	6/15	6/3402	3	411	3	411
P. yunanensis	Mean	0.029414208	0.16409439	0.007	0.060276515	.03768	.12720
	SE	0.000859171	0.000246317	0.00103382	0.00086375	.004171	.001131
	Median	0	0.162	0.008	0.056	.04750	.12500
	SD	0.036754041	0.043530561	0.005470459	0.028068556	.022071	.036746
	Minimum	0	0.056	0	0.02	0.000	.046
	Maximum	0.118	0.32	0.014	0.173	.065	.212
	No.ind/No.com	61/1830	61/31232	28	1056	28	1056
Total	Mean	0.015928941	0.179770877	.00312	.06228	.01662	.13658
	SE	0.000305507	7.46568E-05	.000391	.000226	.001196	.000231
	Median	0.002	0.178	0.00000	.05600	.00900	.13500
	SD	0.029129097	0.042716104	.006082	.031158	.018492	.031871
	Minimum	0	0.056	0.000	.003	0.000	.041
	Maximum	0.118	0.349	.039	.240	.065	.245
	No.ind/No.com	573/9091	573/327374	242	18976	239	18962

Table S3. Comparison of species delineation metrics from Geneious using data from COI, ITS2 and COI+ITS2.

			COI			
Species	P_{ID}(Strict)	P_{ID}(Liberal)	P_{RD}(Randomly Distinct)	Rosenberg's P_{AB} statistic	Monophyletic?	Closest Species
P. bibulba	0.87	0.96	<0.05	NAN	YES	*P. signata*
P. bicruris	0.82	0.97	0.99	1.00E-04	YES	*P. sp13*
P. cangschana	0.97	0.99	<0.05	1.70E-24	YES	*P. rivicola*
P. confusa	0.47	0.84	0.69	7.60E-04	YES	*P. sp10*
P. daliensis	0.92	0.99	<0.05	2.60E-06	YES	*P. kunmingensis*
P. digitata	0.85	0.98	<0.05	1.10E-06	YES	*P. gibberosa*
P. gibberosa	0.96	0.99	<0.05	1.10E-06	YES	*P. digitata*
P. interposita	0.96	0.99	<0.05	1.90E-16	YES	*P. sp15*
P. kunmingensis	0.94	0.99	<0.05	2.60E-06	YES	*P. sp8*
P. lushanensis	0.88	0.97	<0.05	<0.05	YES	*P. sp5*
P. mediana	0.9	0.97	0.62	0.02	YES	*P. sp14*
P. namkhan	0.81	0.94	<0.05	1.00E-04	YES	*P. bicruris*
P. prompta	0.00E+00	0.96	NA	1.35E-03	YES	*P. sp1*
P. recta	0.79	0.92	1	0.04	YES	*P. serrata*
P. rivicola	0.96	0.99	<0.05	4.70E-10	YES	*P. sp11*
P. roganda	0.98	1	<0.05	1.20E-18	YES	*P. sp8*
P. semiannulata	0.77	0.99	<0.05	NAN	YES	*P. prompta*
P. serrata	0.00E+00	0.96	NA	0.04	YES	*P. recta*
P. signata	0.91	0.97	<0.05	6.40E-23	YES	*P. yunnanensis*
P. sinapophysis	0.96	0.99	<0.05	5.40E-35	YES	*P. interposita*
P.sp1	0.78	1	<0.05	6.40E-06	YES	*P. prompta*
P.sp2	0.91	0.98	<0.05	0.01	YES	*P. sp12*
P.sp3	0.92	0.99	<0.05	5.10E-06	YES	*P. sp4*
P.sp4	0.58	0.97	<0.05	0.01	YES	*P. sp5*
P.sp5	0.00E+00	0.96	NA	<0.05	YES	*P. sp4*
P.sp6	0.94	0.98	<0.05	1.10E-04	YES	*P. sp9*
P.sp7	0.58	0.96	<0.05	2.70E-04	YES	*P. spiculata*
P.sp8	0.89	0.97	<0.05	2.00E-09	YES	*P. kunmingensis*
P.sp9	0.73	0.95	<0.05	1.10E-04	YES	*P. sp6*
P.sp10	0.00E+00	0.96	NA	0.01	YES	*P. sp13*
P.sp11	0.96	0.99	<0.05	4.70E-10	YES	*P. rivicola*
P.sp12	0.73	0.95	<0.05	0.01	YES	*P. sp2*
P.sp13	0.00E+00	0.96	NA	0.01	YES	*P. bicruris*
P.sp14	0.58	0.97	<0.05	0.02	YES	*P. mediana*
P. sp15	0.94	0.98	<0.05	1.90E-16	YES	*P. interposita*
P. sp16	0.97	0.99	<0.05	2.20E-23	YES	*P. sinapophysis*
P. sp17	0.00E+00	0.96	NA	3.62E-03	YES	*P. sp18*
P. sp18	0.92	0.97	<0.05	3.62E-03	YES	*P. yunnanensis*

P. sp19	0.9	0.97	0.12	1.20E-04	YES	*P. sp12*
P. spiculata	0.94	0.98	<0.05	2.70E-04	YES	*P. sp7*
P. tiantangensis	0.86	0.97	<0.05	9.00E-08	YES	*P. sp7*
P. yunnanensis	0.88	0.97	NA	NA	NO	*P. signata*

ITS2

Species	P_{ID}(Strict)	P_{ID}(Liberal)	P_{RD}(Randomly Distinct)	Rosenberg's P_{AB} statistic	Monophyletic?	Closest Species
P. bibulba	0.81	0.94	<0.05	4.80E-09	YES	*P. sp17*
P. bicruris	0.58	0.83	<0.05	2.98E-03	YES	*P. namkhan*
P. cangschana	0.63	0.87	<0.05	0.02	YES	*P. rivicola*
P. confusa	0.00E+00	0.96	NA	0.01	YES	*P. sp1*
P. daliensis	0.53	0.78	<0.05	0.01	YES	*P. kunmingensis*
P. digitata	0.71	0.92	<0.05	0.01	YES	*P. gibberosa*
P. gibberosa	0.67	0.9	<0.05	0.01	YES	*P. digitata*
P. interposita	0.74	0.94	<0.05	2.30E-04	YES	*P. sinapophysis*
P. kunmingensis	0.65	0.89	<0.05	1.21E-03	YES	*P. daliensis*
P. lushanensis	0.68	0.91	<0.05	6.40E-06	YES	*P. sp8*
P. mediana	0.75	0.97	<0.05	6.10E-05	YES	*P. sp5*
P. namkhan	0.83	0.93	<0.05	1.10E-05	YES	*P. bicruris*
P. recta	0.78	0.95	NA	NA	NO	*P. serrata*
P. rivicola	0.63	0.87	<0.05	0.02	YES	*P. cangschana*
P. roganda	0.71	0.94	<0.05	1.10E-04	YES	*P. digitata*
P. semiannulata	0.72	0.95	0.92	3.40E-08	YES	*P. sp19*
P. serrata	0.00E+00	0.96	NA	0.33	YES	*P. recta*
P. signata	0.75	0.94	<0.05	5.10E-05	YES	*P. yunnanensis*
P. sinapophysis	0.71	0.94	<0.05	2.98E-03	YES	*P. interposita*
P. sp1	0.63	0.87	<0.05	6.10E-05	YES	*P. sp8*
P. sp2	0.71	0.94	<0.05	0.02	YES	*P. sp12*
P. sp3	0.49	0.86	<0.05	0.11	YES	*P. sp4*
P. sp4	0.53	0.91	<0.05	0.11	YES	*P. sp3*
P. sp5	0.00E+00	0.96	NA	1.16E-03	YES	*P. tiantangensis*
P. sp6	0.8	0.95	<0.05	2.98E-03	YES	*P. sp9*
P. sp7	0.45	0.81	<0.05	<0.05	YES	*P. spiculata*
P. sp8	0.52	0.9	<0.05	3.10E-04	YES	*P. kunmingensis*
P. sp9	0.67	0.9	<0.05	2.98E-03	YES	*P. sp6*
P. sp10	0.00E+00	0.96	NA	0.01	YES	*P. namkhan*
P. sp11	0.86	0.97	<0.05	6.60E-08	YES	*P. sp16*
P. sp12	0.71	0.94	<0.05	0.02	YES	*P. sp2*
P. sp13	0.00E+00	0.96	NA	0.01	YES	*P. confusa*
P. sp14	0.57	0.96	<0.05	1.40E-06	YES	*P. sp19*
P. sp15	0.62	0.88	<0.05	2.98E-03	YES	*P. sinapophysis*
P. sp16	0.73	0.96	<0.05	3.50E-06	YES	*P. sp11*
P. sp17	0.00E+00	0.96	NA	4.76E-03	YES	*P. bibulba*
P. sp18	0.77	0.94	<0.05	2.70E-06	YES	*P. sp17*

P. sp19	0.72	0.93	<0.05	1.10E-09	YES	*P. roganda*
P. spiculata	0.54	0.8	<0.05	<0.05	YES	*P. sp7*
P. tiantangensis	0.67	0.9	<0.05	4.60E-06	YES	*P. sp5*
P. yunnanensis	0.76	0.91	<0.05	5.10E-05	YES	*P. signata*
			COI+ITS2			
Species	P_{ID}(Strict)	P_{ID}(Liberal)	P_{RD}(Randomly Distinct)	Rosenberg's P_{AB} statistic	Monophyletic?	Closest Species
P. bibulba	0.81	0.94	<0.05	4.80E-09	YES	*P. signata*
P. bicruris	0.74	0.96	<0.05	1.85E-03	YES	*P. namkhan*
P. cangschana	0.77	0.98	<0.05	1.21E-03	YES	*P. rivicola*
P. confusa	0.00E+00	0.96	NA	0.01	YES	*P. sp13*
P. daliensis	0.76	0.98	<0.05	0.02	YES	*P. kunmingensis*
P. digitata	0.86	0.98	<0.05	0.01	YES	*P. gibberosa*
P. gibberosa	0.75	0.97	<0.05	0.01	YES	*P. digitata*
P. interposita	0.82	0.97	<0.05	2.30E-04	YES	*P. sinapophysis*
P. kunmingensis	0.76	0.98	<0.05	0.02	YES	*P. sp8*
P. lushanensis	0.75	0.97	<0.05	0.17	YES	*P. sp5*
P. mediana	0.78	0.99	<0.05	6.10E-05	YES	*P. sp19*
P. namkhan	0.83	0.93	<0.05	1.85E-03	YES	*P. bicruris*
P. recta	0.8	0.95	<0.05	0.07	YES	*P. serrata*
P. rivicola	0.77	0.99	<0.05	0.01	YES	*P. cangschana*
P. roganda	0.78	1	<0.05	3.50E-06	YES	*P. sp8*
P. semiannulata	0.77	0.99	<0.05	9.80E-08	YES	*P. sp19*
P. serrata	0.00E+00	0.96	NA	0.07	YES	*P. recta*
P. signata	0.79	0.95	<0.05	5.10E-05	YES	*P. yunnanensis*
P. sinapophysis	0.77	0.99	<0.05	2.98E-03	YES	*P. interposita*
P. sp1	0.78	1	<0.05	1.10E-04	YES	*P. sp8*
P. sp2	0.78	1	<0.05	0.02	YES	*P. sp12*
P. sp3	0.57	0.96	<0.05	0.11	YES	*P. sp4*
P. sp4	0.58	0.98	<0.05	0.11	YES	*P. sp3*
P. sp5	0.00E+00	0.96	NA	0.17	YES	*P. sp4*
P. sp6	0.87	0.97	<0.05	2.98E-03	YES	*P. sp9*
P. sp7	0.58	0.97	<0.05	<0.05	YES	*P. spiculata*
P. sp8	0.51	0.89	0.07	0.01	YES	*P. kunmingensis*
P. sp9	0.74	0.96	<0.05	2.98E-03	YES	*P. sp6*
P. sp10	0.00E+00	0.96	NA	0.01	YES	*P. sp13*
P. sp11	0.91	0.98	<0.05	0.01	YES	*P. rivicola*
P. sp12	0.75	0.97	0.97	0.02	YES	*P. sp2*
P. sp13	0.00E+00	0.96	NA	0.01	YES	*P. sp10*
P. sp14	0.59	0.98	0.16	1.40E-06	YES	*P. sp19*
P. sp15	0.83	0.96	<0.05	2.98E-03	YES	*P. sinapophysis*
P. sp16	0.78	1	<0.05	2.30E-04	YES	*P. interposita*
P. sp17	0.00E+00	0.96	NA	<0.05	YES	*P. sp18*
P. sp18	0.76	0.94	<0.05	<0.05	YES	*P. signata*

P. sp19	0.84	0.97	0.06	2.40E-09	YES	*P. sp8*
P. spiculata	0.76	0.98	<0.05	<0.05	YES	*P. sp7*
P. tiantangensis	0.75	0.97	<0.05	0.01	YES	*P. sp7*
P. yunnanensis	0.63	0.87	<0.05	5.10E-05	YES	*P. signata*

Table S4. Results of the Automatic Barcode Gap Discovery (ABGD) analyses using data from COI, ITS2 and COI+ITS2.

Results of the Automatic Barcode Gap Discovery (ABGD) analyses

COI

Subst.model	Pmin/Pmax	X	Partition	Prior intraspecific divergence (P)							
				0.0010	0.0017	0.0028	0.0046	0.0077	0.0129	0.0215	0.0359
JC	0.001/0.1	1.5	Initial	154	48	48	48	48	48	48	48
			Recursive	154	86	71	68	59	53	49	48
K2P	0.001/0.1	1.5	Initial	154	48	48	48	48	48	48	48
			Recursive	154	86	71	68	59	53	49	48
Simple	0.001/0.1	1.5	Initial	272	67	67	67	67	67	67	67
			Recursive	272	93	93	85	80	72	70	67
JC	0.001/0.1	1	Initial	154	94	94	48	48	48	48	48
			Recursive	154	102	96	68	59	55	49	48
K2P	0.001/0.1	1	Initial	154	48	48	48	48	48	48	48
			Recursive	154	86	72	68	59	55	49	48
Simple	0.001/0.1	1	Initial	272	67	67	67	67	67	67	67
			Recursive	272	97	96	86	81	73	70	68
				0.0001	0.0002	0.0005	0.0013	0.0029	0.0068	0.0159	0.0369
JC	0.0001/0.2	1.5	Initial	154	154	154	154	48	48	48	48
			Recursive	154	154	154	154	71	59	50	48
K2P	0.0001/0.2	1.5	Initial	154	154	154	154	48	48	48	48
			Recursive	154	154	154	154	71	59	50	48
Simple	0.0001/0.2	1.5	Initial	272	272	272	272	67	67	67	67
			Recursive	272	272	272	272	93	80	71	67
JC	0.0001/0.2	1	Initial	154	154	154	94	48	48	48	48
			Recursive	154	154	154	154	96	59	53	48
K2P	0.0001/0.2	1	Initial	154	154	154	154	72	48	48	48
			Recursive	154	154	154	154	72	59	53	48
Simple	0.0001/0.2	1	Initial	272	272	272	272	67	67	67	67
			Recursive	272	272	272	272	96	81	71	68

ITS2

Subst.model	Pmin/Pmax	X	Partition	Prior intraspecific divergence (P)						
				0.0010	0.0017	0.0028	0.0046	0.0077	0.0129	0.0215
JC	0.001/0.1	1	Initial	65	65	1				
			Recursive	65	65	1				
K2P	0.001/0.1	1	Initial	65	65	1				
			Recursive	65	65	1				
Simple	0.001/0.1	1	Initial	73	73	1				
			Recursive	73	73	1				
JC	0.001/0.1	0.5	Initial	65	65	31	31	31	31	1
			Recursive	65	65	46	46	37	34	1
K2P	0.001/0.1	0.5	Initial	65	65	31	31	31	31	1
			Recursive	65	65	46	46	39	34	1

Simple	0.001/0.1	0.5	Initial	73	73	58	58	38	29	1	
			Recursive	73	73	58	58	25	25	1	
				0.0001	0.0002	0.0005	0.0013	0.0029	0.0068	0.0159	
JC	0.0001/0.2	1	Initial	65	65	65	65	1			
			Recursive	65	65	65	65	1			
K2P	0.0001/0.2	1	Initial	65	65	65	65	1			
			Recursive	65	65	65	65	1			
Simple	0.0001/0.2	1	Initial	73	73	73	73	1			
			Recursive	73	73	73	73	1			
JC	0.0001/0.2	0.5	Initial	65	65	65	65	31	31	1	
			Recursive	65	65	65	65	46	37	1	
K2P	0.0001/0.2	0.5	Initial	65	65	65	65	31	31	1	
			Recursive	65	65	65	65	46	39	1	
Simple	0.0001/0.2	0.5	Initial	73	73	73	73	58	25	25	
			Recursive	73	73	73	73	58	38	27	

				COI+ITS2							
Subst.model	Pmin/Pmax	X	Partition	Prior intraspecific divergence (P)							
				0.0010	0.0017	0.0028	0.0046	0.0077	0.0129	0.0215	0.0359
JC	0.001/0.1	1	Initial	44	44	44	44	44	44	44	1
			Recursive	55	54	54	50	49	46	44	1
K2P	0.001/0.1	1	Initial	44	44	44	44	44	44	44	1
			Recursive	55	54	54	50	49	46	44	1
Simple	0.001/0.1	1	Initial	63	63	63	63	63	1		
			Recursive	66	66	65	65	64	1		
JC	0.001/0.1	0.5	Initial	44	44	44	44	44	44	44	32
			Recursive	58	57	56	51	50	47	45	38
K2P	0.001/0.1	0.5	Initial	44	44	44	44	44	44	44	32
			Recursive	58	57	56	51	50	47	45	38
Simple	0.001/0.1	0.5	Initial	63	63	63	63	63	51	47	1
			Recursive	66	66	65	65	64	53	49	1
				0.0001	0.0002	0.0005	0.0013	0.0029	0.0068	0.0159	0.0369
JC	0.0001/0.2	1	Initial	44	44	44	44	44	44	44	
			Recursive	55	55	55	54	54	49	46	
K2P	0.0001/0.2	1	Initial	44	44	44	44	44	44	44	
			Recursive	55	55	55	54	54	49	46	
Simple	0.0001/0.2	1	Initial	63	63	63	63	63	63	1	
			Recursive	66	66	66	66	65	64	1	
JC	0.0001/0.2	0.5	Initial	44	44	44	44	44	44	44	32
			Recursive	58	58	58	57	56	50	47	38
K2P	0.0001/0.2	0.5	Initial	44	44	44	44	44	44	44	32
			Recursive	58	58	58	57	56	50	47	38
Simple	0.0001/0.2	0.5	Initial	63	63	63	63	63	63	51	1
			Recursive	66	66	66	66	65	64	52	1
